# The burden of metabolic risk factors in North Africa and the Middle East, 1990–2019: findings from the Global Burden of Disease Study

**DOI:** 10.1016/j.eclinm.2023.102022

**Published:** 2023-06-02

**Authors:** Mohammad-Reza Malekpour, Mohsen Abbasi-Kangevari, Seyyed-Hadi Ghamari, Javad Khanali, Mahsa Heidari-Foroozan, Sahar Saeedi Moghaddam, Mohammadreza Azangou-Khyavy, Sahba Rezazadeh-Khadem, Negar Rezaei, Parnian Shobeiri, Zahra Esfahani, Nazila Rezaei, GBD 2019 North Africa and the Middle East MRF Collaborators, Mohammad-Reza Malekpour, Mohsen Abbasi-Kangevari, Seyyed-Hadi Ghamari, Javad Khanali, Mahsa Heidari-Foroozan, Sahar Saeedi Moghaddam, Mohammadreza Azangou-Khyavy, Sahba Rezazadeh-Khadem, Negar Rezaei, Parnian Shobeiri, Zahra Esfahani, Nazila Rezaei, Amirali Aali, Sherief Abd-Elsalam, Meriem Abdoun, Abdorrahim Absalan, Eman Abu-Gharbieh, Niveen ME. Abu-Rmeileh, Ahmed Abu-Zaid, Ali Ahmadi, Sepideh Ahmadi, Ayman Ahmed, Tarik Ahmed Rashid, Marjan Ajami, Mostafa Akbarzadeh-Khiavi, Hanadi Al Hamad, Tariq A. Alalwan, Khalid F. Alhabib, Yousef Alimohamadi, Vahid Alipour, Syed Mohamed Aljunid, Mahmoud A. Alomari, Saleh A. Alqahatni, Rajaa M. Al-Raddadi, Javad Javad Aminian Dehkordi, Mehrdad Amir-Behghadami, Sohrab Amiri, Davood Anvari, Jalal Arabloo, Judie Arulappan, Ashokan Arumugam, Zahra Aryan, Mohammad Athar, Seyyed Shamsadin Athari, Abolfazl Avan, Sina Azadnajafabad, Samad Azari, Hosein Azizi, Nayereh Baghcheghi, Nader Bagheri, Sara Bagherieh, Ovidiu Constantin Baltatu, Akshaya Srikanth Bhagavathula, Vijayalakshmi S. Bhojaraja, Souad Bouaoud, Muhammad Hammad Butt, Luciana Aparecida Campos, Abdulaal Chitheer, Reza Darvishi Cheshmeh Soltani, Aso Mohammad Darwesh, Shirin Djalalinia, Milad Dodangeh, Maysaa El Sayed Zaki, Iffat Elbarazi, Muhammed Elhadi, Waseem El-Huneidi, Rana Ezzeddini, Mohammad Fareed, Hossein Farrokhpour, Ali Fatehizadeh, Yaseen Galali, Amir Ghaderi, Mansour Ghafourifard, Mohammad Ghasemi Nour, Ahmad Ghashghaee, Maryam Gholamalizadeh, Pouya Goleij, Mohamad Golitaleb, Parham Habibzadeh, Nima Hafezi-Nejad, Rabih Halwani, Hamidreza Hasani, Maryam Hashemian, Amr Hassan, Soheil Hassanipour, Hadi Hassankhani, Kamal Hezam, Reza Homayounfar, Seyed Kianoosh Hosseini, Kaveh Hosseini, Mehdi Hosseinzadeh, Soodabeh Hoveidamanesh, Jalil Jaafari, Haitham Jahrami, Elham Jamshidi, Tahereh Javaheri, Sathish Kumar Jayapal, Ali Kabir, Amirali Karimi, Neda Kaydi, Mohammad Keykhaei, Yousef Saleh Khader, Morteza Abdullatif Khafaie, Moien A.B. Khan, Kashif Ullah Khan, Yusra H. Khan, Moawiah Mohammad Khatatbeh, Farzad Kompani, Hamid Reza Koohestani, Mohammed Kuddus, Savita Lasrado, Sang-woong Lee, Soleiman Mahjoub, Ata Mahmoodpoor, Elham Mahmoudi, Elaheh Malakan Rad, Narges Malih, Ahmad Azam Malik, Tauqeer Hussain Mallhi, Yosef Manla, Borhan Mansouri, Mohammad Ali Mansournia, Parham Mardi, Abdoljalal Marjani, Sahar Masoudi, Entezar Mehrabi Nasab, Ritesh G. Menezes, Vildan Mevsim, Yousef Mohammad, Mokhtar Mohammadi, Esmaeil Mohammadi, Noushin Mohammadifard, Arif Mohammed, Sara Momtazmanesh, Fateme Montazeri, Maryam Moradi, Maziar Moradi-Lakeh, Negar Morovatdar, Christopher J.L. Murray, Zuhair S. Natto, Seyed Aria Nejadghaderi, Ali Nowroozi, Morteza Oladnabi, Ahmed Omar Bali, Emad Omer, Hamidreza Pazoki Toroudi, Raffaele Pezzani, Ashkan Pourabhari Langroudi, Sima Rafiei, Mehran Rahimi, Vafa Rahimi-Movaghar, Shayan Rahmani, Amir Masoud Rahmani, Vahid Rahmanian, Chythra R. Rao, Sina Rashedi, Mohammad-Mahdi Rashidi, Reza Rawassizadeh, Elrashdy Moustafa Mohamed Redwan, Malihe Rezaee, Maryam Rezaei, Seyed Mohammad Riahi, Gholamreza Roshandel, Aly Saad, Maha Mohamed Saber-Ayad, Siamak Sabour, Leila Sabzmakan, Basema Saddik, Erfan Sadeghi, Saeid Sadeghian, Amirhossein Sahebkar, Morteza Saki, Saina Salahi, Sarvenaz Salahi, Amir Salek Farrokhi, Marwa Rashad Salem, Hamideh Salimzadeh, Abdallah M. Samy, Nizal Sarrafzadegan, Brijesh Sathian, Melika Shafeghat, Syed Mahboob Shah, Jaffer Shah, Ataollah Shahbandi, Fariba Shahraki-Sanavi, Mehran Shams-Beyranvand, Mohd Shanawaz, Kiomars Sharafi, Javad Sharifi-Rad, Jeevan K. Shetty, Zahra Shokri Varniab, Seyed Afshin Shorofi, Soraya Siabani, Mohammad Sadegh Soltani-Zangbar, Seidamir Pasha Tabaeian, Seyed-Amir Tabatabaeizadeh, Mohammad Tabish, Majid Taheri, Yasaman Taheri Abkenar, Moslem Taheri Soodejani, Amir Taherkhani, Arash Tehrani-Banihashemi, Mohamad-Hani Temsah, Bereket M. Tigabu, Alireza Vakilian, Siavash Vaziri, Bay Vo, Fereshteh Yazdanpanah, Arzu Yigit, Vahit Yiğit, Mazyar Zahir, Burhan Abdullah Zaman, Maryam Zamanian, Moein Zangiabadian, Iman Zare, Zahra Zareshahrabadi, Ali H. Mokdad, Mohsen Naghavi, Bagher Larijani, Farshad Farzadfar, Ali H. Mokdad, Mohsen Naghavi, Bagher Larijani, Farshad Farzadfar

**Affiliations:** a Non-Communicable Diseases Research Center, Endocrinology and Metabolism Population Sciences Institute, Tehran University of Medical Sciences, Tehran, Iran; b Kiel Institute for the World Economy, Kiel, Germany; c Institute for Health Metrics and Evaluation, University of Washington, Seattle, WA, USA; d Department of Health Metrics Sciences, School of Medicine, University of Washington, Seattle, WA, USA; e Endocrinology and Metabolism Research Center, Endocrinology and Metabolism Clinical Sciences Institute, Tehran University of Medical Sciences, Tehran, Iran

**Keywords:** Global burden of disease, Metabolic risk factors, High systolic blood pressure, High fasting plasma glucose, High body-mass index, High-LDL, North Africa and the Middle East

## Abstract

**Background:**

The objective of this study is to investigate the trends of exposure and burden attributable to the four main metabolic risk factors, including high systolic blood pressure (SBP), high fasting plasma glucose (FPG), high body-mass index (BMI), and high low-density lipoproteins cholesterol (LDL) in North Africa and the Middle East from 1990 to 2019.

**Methods:**

The data were retrieved from Global Burden of Disease Study 2019. Summary exposure value (SEV) was used for risk factor exposure. Burden attributable to each risk factor was incorporated in the population attributable fraction to estimate the total attributable deaths and disability-adjusted life-years (DALYs).

**Findings:**

While age-standardized death rate (ASDR) attributable to high-LDL and high-SBP decreased by 26.5% (18.6–35.2) and 23.4% (15.9–31.5) over 1990–2019, respectively, high-BMI with 5.1% (−9.0–25.9) and high-FPG with 21.4% (7.0–37.4) change, grew in ASDR. Moreover, age-standardized DALY rate attributed to high-LDL and high-SBP declined by 30.2% (20.9–39.0) and 25.2% (16.8–33.9), respectively. The attributable age-standardized DALY rate of high-BMI with 8.3% (−6.5–28.8) and high-FPG with 27.0% (14.3–40.8) increase, had a growing trend. Age-standardized SEVs of high-FPG, high-BMI, high-SBP, and high-LDL increased by 92.4% (82.8–103.3), 76.0% (58.9–99.3), 10.4% (3.8–18.0), and 5.5% (4.3–7.1), respectively.

**Interpretation:**

The burden attributed to high-SBP and high-LDL decreased during the 1990–2019 period in the region, while the attributable burden of high-FPG and high-BMI increased. Alarmingly, exposure to all four risk factors increased in the past three decades. There has been significant heterogeneity among the countries in the region regarding the trends of exposure and attributable burden. Urgent action is required at the individual, community, and national levels in terms of introducing effective strategies for prevention and treatment that account for local and socioeconomic factors.

**Funding:**

10.13039/100000865Bill & Melinda Gates Foundation.

**Research in context**

**Evidence before this study**

We searched databases PubMed, Web of Science, and Scopus in February 2022 using search terms (“metabolic risk factor∗” OR “high systolic blood pressure” OR “high fasting plasma glucose” OR “high fasting blood sugar” OR “high body-mass index” OR “high BMI” OR “high low-density lipoproteins” OR “high LDL”) AND (“North Africa” OR “Middle East”) AND (“death” OR “mortality” OR “DALY” OR “morbidity” OR “burden of disease” OR “epidemiology”). We found evidence on the prevalence of the risk factors from internationally-supported national-wide surveys such as STEPs (data is available for Iran, Iraq, Lebanon, Jordan, Kuwait, Algeria, Palestine, Egypt, Turkey, Qatar, Sudan, and Saudi Arabia), national health survey projects (e.g., TURDEP, TAHINA, Trabzon studies), subnational population surveys (e.g., Isfahan healthy heart program, the Tehran lipid and glucose study, Abu Dhabi cardiovascular screening program), and cohort studies (e.g., Golestan, Pars, PERSIAN, and PURE studies). However, there was a paucity of published literature that explored the prevalence, exposure, and burden of metabolic risk factors with a holistic view on North Africa and the Middle East (NAME), a region with poor metabolic health and diverse socioeconomic settings. There is only a review article by Azizi et al, published in 2019 that applied the results of the Global Burden of Disease Study (GBD) 2017 study, combined with other published data from the United Nations, the World Health Organization, the International Diabetes Federation, and Non-communicable Diseases Risk Factor Collaboration to provide an overview of the metabolic health in the region. There is no report from GBD 2019 to investigate the current status and trend of exposure and burden of metabolic risk factors, focusing on the NAME.

**Added value of this study**

This article provides detailed insight into the latest status as well as three-decade trends of the burden attributable to metabolic risk factors in all 21 countries of NAME. Using the GBD framework, we showed that while age-standardized death rate attributable to high low-density lipoproteins and high systolic blood pressure had a decreasing pattern, high body-mass index and high fasting plasma glucose were increasing over 1990–2019. In the same period, exposure to all four risk factors increased. However, there has been significant heterogeneity among the countries of the region in the trends of exposure and attributable burden.

**Implications of all the available evidence**

The increasing trend of age-standardized exposure to metabolic risk factors drives the epidemic of non-communicable diseases in NAME. Vigorous preventions program with comprehensive surveillance and monitoring, as well as joint collaborations between countries of the region, are urgently needed to improve metabolic health in this region.

## Introduction

Metabolic risk factors (MRF) lead to almost one-third of all global deaths and more than 460 million disability-adjusted life years (DALYs).^1^ Despite the previous efforts, exposure to MRF not only increased from 1990 to 2019 but also experienced a steeper growth trend from 2010 to 2019 comparing the 1990–2010 period.^1^ High systolic blood pressure (SBP), high fasting plasma glucose (FPG), high body-mass index (BMI), and high low-density lipoproteins cholesterol (LDL) together make up the main part of metabolic risk factors, accounting for more than a quarter of global DALYs. In addition, each of the mentioned risk factors was among the top ten risks by attributable DALYs in 2019.^2^

Attaining valid estimations of metabolic risk factors would be essential yet challenging for a region like North Africa and the Middle East (NAME) with its conspicuous features. North Africa and the Middle East had the highest rate of age-standardized DALYs attributable to MRF among all Global Burden of Diseases Study (GBD) super-regions in 2019.^1^ Economic changes, especially the oil industry's growth in recent decades, have increased health-related expenditure and, consequently, led to improved life expectancy. Furthermore, unhealthy modern lifestyle with low physical activity and diets rich in fats has escalated the burden of non-communicable diseases.^3^ This heterogeneous region comprises 21 countries with diverse cultures and social contexts. The socio-demographic index (SDI) varies among these countries, from 0.88 for the United Arab Emirates (UAE) to 0.34 for Afghanistan in 2019.^4^ The index is a summary measure that combines information on the economy, education, and fertility rate of countries in the GBD studies and is shown to be closely tied to health outcomes. Moreover, some countries of the region experience the detrimental effect of social conflicts and wars, which have led to the militarization and regionalization of health care in some countries of the region, complicating the rebuilding of previously robust national healthcare systems.^5^

There is still a paucity of comparable, consistent, and regularly updated quantification of MRF exposure and burden in the NAME region. Published literature from different iterations of the GBD study has explored the global burden of risk factors, however, none of them are focused on MRF in the NAME region. There are also national and subnational cross-sectional studies mainly on the prevalence of a single metabolic risk factor in a country of the region. Therefore, it is not evident whether previous efforts to mitigate MRF exposure and burden have been efficacious in downsloping its trend. The objective of this study is to investigate the status and trend of exposure and burden attributable to the four main MRF, including high-SBP, high-FPG, high-BMI, and high-LDL in North Africa and the Middle East from 1990 to 2019 using the GBD 2019 results. We drafted this manuscript as part of the GBD Collaborator Network under the guidance of the GBD protocol.

## Methods

### Overview

We obtained the data in this study from GBD 2019 public datasets available from https://ghdx.healthdata.org/gbd-results-tool (accessed on April, 2021). The 21 countries of the NAME super-region of GBD were investigated in this study, including Afghanistan, Algeria, Bahrain, Egypt, Iran, Iraq, Jordan, Kuwait, Lebanon, Libya, Morocco, Oman, Palestine, Qatar, Saudi Arabia, Sudan, Syria, Tunisia, Turkey, the UAE, and Yemen.

The detailed methods and standardized data collection protocol have been published previously.^1^ A brief overview of the GBD 2019 methods is presented here. In the GBD 2019, the six-step framework of comparative risk assessment (CRA) was used to estimate the burden attributable to MRF.^6^ The six steps of CRA are (1) inclusion of the risk–outcome pairs into the analysis ; (2) relative risk estimation as a function of exposure; (3) exposure levels and distributions estimation; (4) computation of the theoretical minimum risk exposure level (TMREL) as the counterfactual level of exposure; (5) calculation of population attributable fractions (PAF) and attributable burden; (6) and estimation of the mediating effects of various risk factors on each other to determine the burden of a combination of risk factors.

In total, 26, 41, 157, and 20 data sources were used to analyze high-SBP, high-FPG, high-BMI, and high-LDL, respectively. Of the included data sources, 107 were obtained through surveys and 60 were provided by the government. Besides, Turkey had the most available data sources with 49, followed by Iran with 24. In contrast, Afghanistan, Libya, and Syria with 3 data sources each, had the lowest available data sources (Supplementary Table S1).

### Summary exposure value

The summary exposure value (SEV) is the risk-weighted prevalence of exposure which is defined as below for continuous risks:

$$SEV=\frac{\int_{x=1}^{u}RR\left(x\right)P\left(x\right)dx-1}{{RR}_{\max}-1}$$

where *RR*(*x*) is level *x* of exposure relative risk, *RR*_*max*_ is the relative risk at the 99th percentile of the global distribution of exposure, and *P*(*x*) is the density of exposure at level *x*. SEV ranges from 0 to 100, where the value 0 means there is no excess risk for the entire population, and the value 100 means everyone in the population is exposed to the maximum risk.^1^

### Theoretical minimum risk exposure level

High levels of risk factors exposure were determined via TMREL, which was defined by the GBD 2019 as the exposure level with minimum risk at the population level. Accordingly, 110–115 mmHg, 4.8–5.4 mmol/l, 0.7–1.3 mmol/l, and 20–25 kg/m^2^ for adults and above normal weight for children, were considered as counterfactual levels of exposure for SBP, FPG, LDL, and BMI, respectively.^1^

### Population attributable fraction

Burden attributable to a single risk factor, directly or indirectly, was incorporated in the PAF to estimate the total attributable death and DALYs to that particular risk factor. PAF measures the proportional reduction in the cause-specific burden that would occur if exposure to the risk factor was at TMREL.

### Socio-demographic index

The SDI measure was calculated based on the educational attainment of those aged 15 years or older, lag distributed income per capita, and the total fertility rate among females under 25 years. The SDI ranged from 0 to 1, where higher values indicate higher levels of development. According to SDI-quintiles, countries were divided into five groups: low, low-middle, middle, high-middle, and high SDI.^7^

### Statistical analysis

To better compare the trends and appreciate the compound effect, annualized rate of change (ARC) for the periods 1990–2019, 1990–2010, and 2010–19 as below:

$$ARC=\sqrt[{n}]{\frac{E}{B}}-1$$

where E is the estimate at the end of the period, B is the estimate at the beginning of the period, and n is the period length. Gender disparity ratio (GDR) was calculated by dividing the measured values of females by that of males. To account for change in the population structure, age-standardized death and DALY rates were computed by direct standardization to the global age structure and expressed as the number per 100,000 population. The 95% uncertainty interval (UI) was calculated for each metric using the 25th and 975th ranked draws of the uncertainty distribution by taking 1000 samples from the posterior distribution. Data visualizations were performed using Python programming language, version 3.6, and Tableau Desktop, version 2020.1, an interactive data visualization software.

### Role of the funding source

The funders of the study had no role in study design, data collection, data analysis, data interpretation, or the writing of the report.

## Results

### Overview

High-SBP with 803,631 (95% uncertainty interval [UI]: 687,099–923,807) all-ages deaths had the highest number of attributable deaths in 2019, followed by high-BMI (538,448 [369,917–712,329]), high-FPG (516,784 [389,901–674,873]), and high-LDL (401,217 [309,136–506,066]). The age-standardized death rate (ASDR) attributable to MRF decreased from 458.8 (419.4–499.2) to 373.9 (332.0–414.8) during the 1990–2019 period. Age-standardized DALY rate attributable to MRF declined from 10,159.5 (9296.4–11,024.3) to 8412.8 (7425.6–9400.7). Afghanistan had the highest ASDR attributable to MRF with 590.4 (472.8–698.4), followed by Egypt (534.5 [423.7–657.7]) and Oman (511.9 [462.9–565.3]).

### High systolic blood pressure

**Fig. 1:**
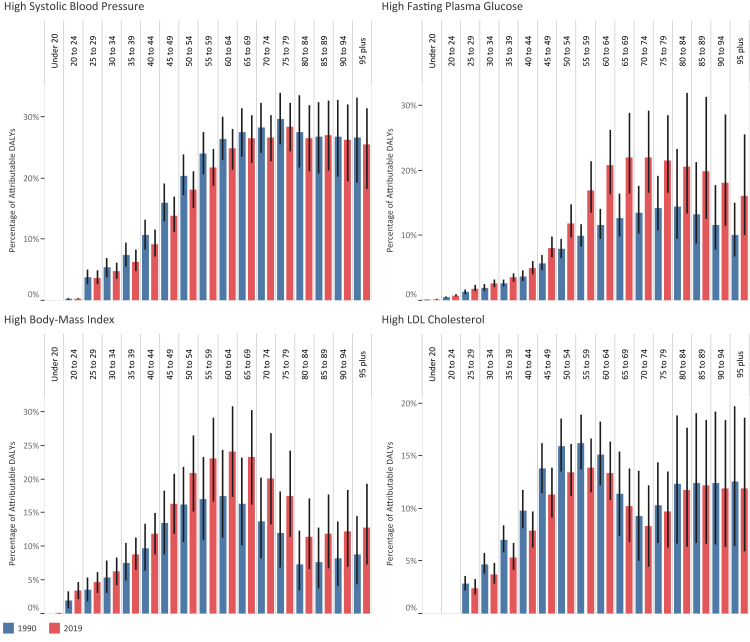
The percentage of DALYs attributable to high-SBP, high-FPG, high-BMI, and high-LDL in 1990 and 2019 by age group.

**Fig. 2:**
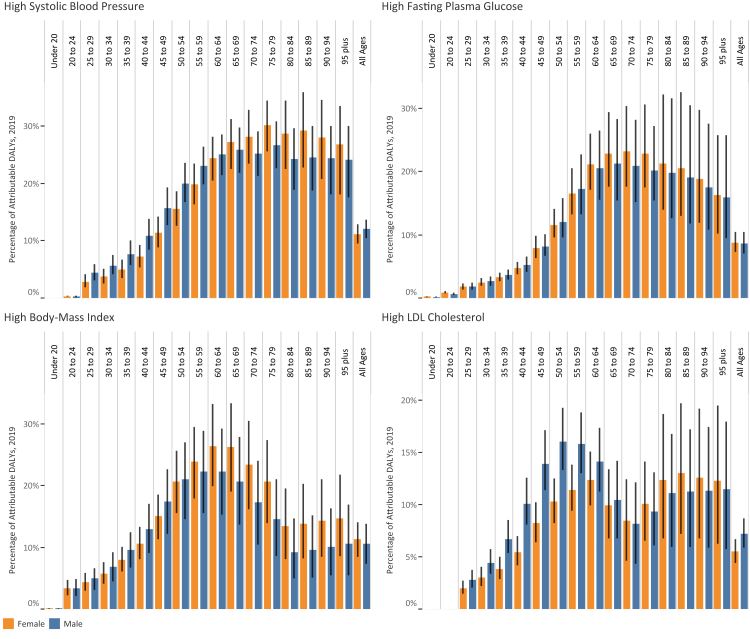
The percentage of DALYs attributable to high-SBP, high-FPG, high-BMI, and high-LDL in genders by age group.

**Fig. 3:**
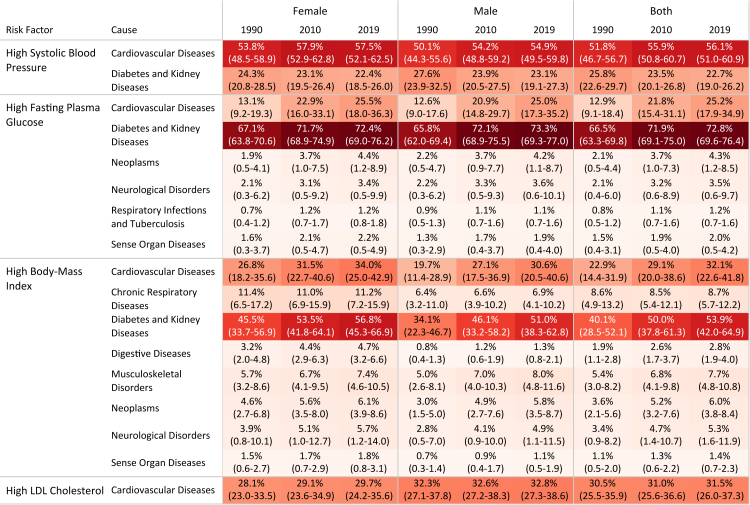
The percentage of age-standardized DALY rate of Level 2 causes attributable to high-SBP, high-FPG, high-BMI, and high-LDL in 1990, 2010, and 2019 by gender.

**Fig. 4:**
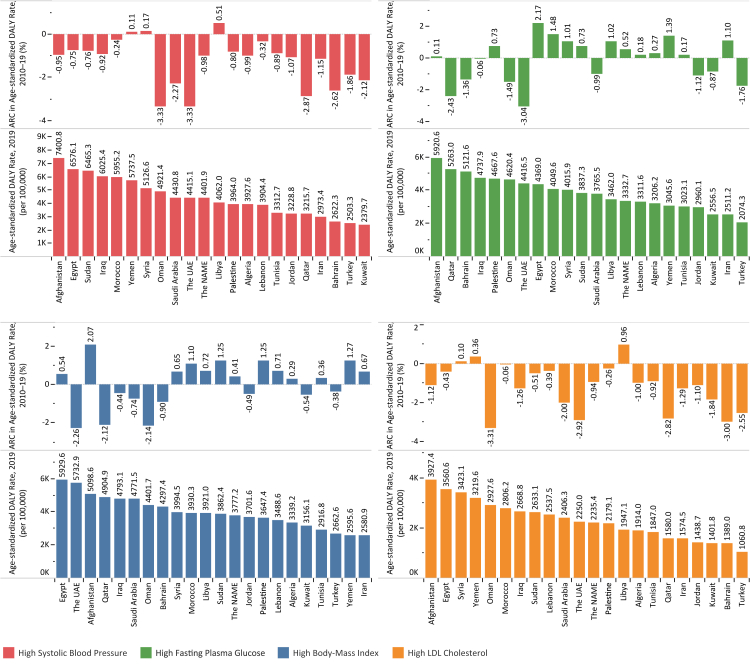
Age-standardized DALY rate in 2019 and ARC of age-standardized DALY rate in 2010–2019 period for high-SBP, high-FPG, high-BMI, and high-LDL among countries of the North Africa and the Middle East region.

**Fig. 5:**
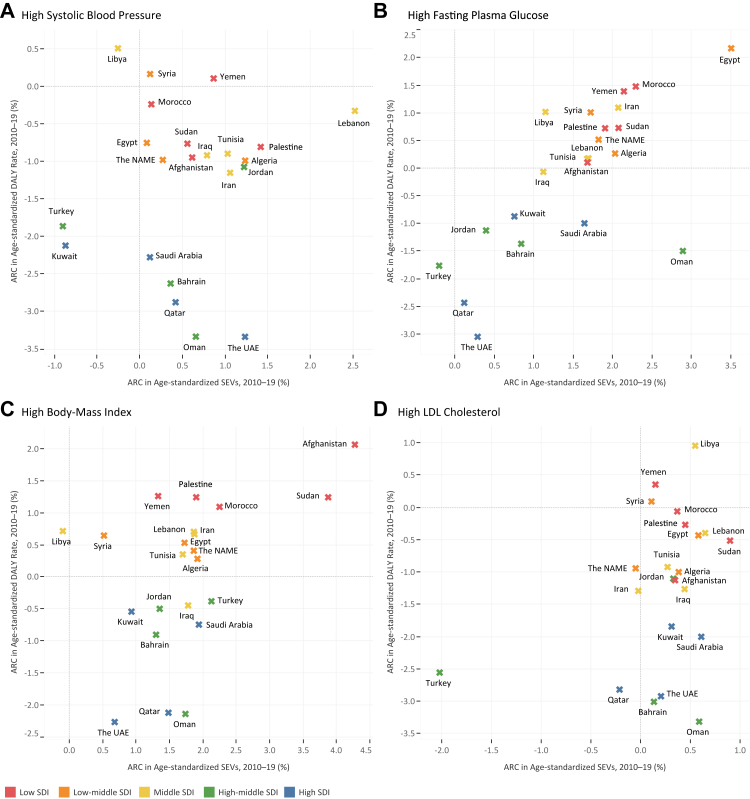
ARC in age-standardized DALY rate compared with age-standardized SEVs during 2010–2019 period for (A) high-SBP, (B) high-FPG, (C) high-BMI, and (D) high-LDL among countries of the North Africa and the Middle East region colored by SDI quintile.

**Fig. 6:**
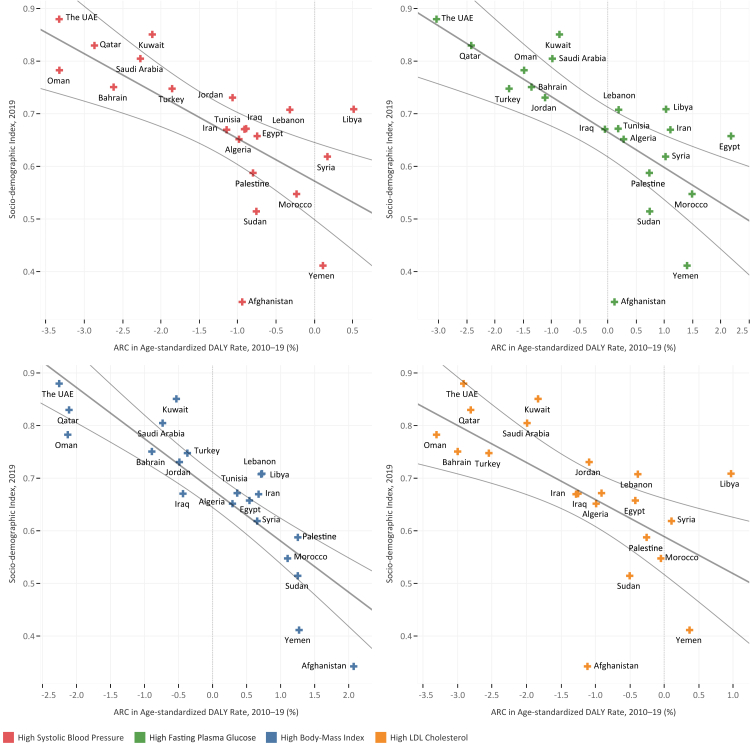
Socio-demographic Index (SDI) in 2019 versus ARC of age-standardized DALY rate in 2010–2019 period for high-SBP, high-FPG, high-BMI, and high-LDL among countries of the North Africa and the Middle East region.

ASDR attributable to high-SBP decreased by 23.4% (15.9–31.5) during the 1990–2019 period, from 286.5 (246.7–323.4) to 219.4 (185.6–252.5) at the annual rate of −0.9%. Age-standardized DALY rate attributable to high-SBP declined by 25.2% (16.8–33.9), from 5887.9 (5221.2–6582.3) to 4401.9 (3785.9–5042.8) (Supplementary Table S2). The percentage of DALYs attributable to high-SBP had an upward trend up to the age group of 60–64 and then reached a relative plateau in both 1990 and 2019 (Fig. 1 and Supplementary Table S3). The percentage of DALYs attributable to high-SBP was higher in males up to the same 60–64 age group in 2019 (Fig. 2 and Supplementary Table S3). High-SBP exerted most of its burden via cardiovascular diseases with attribution to 56.1% (51.0–60.9) of age-standardized DALY rate of cardiovascular diseases in 2019 (Fig. 3 and Supplementary Table S4). Afghanistan (7400.8 [5554.9–9271.6]), Egypt (6576.1 [4975.9–8308.1]), and Sudan (6465.3 [5167.5–8161.9]) suffered the most age-standardized DALY rates attributable to high-SBP, while Kuwait (2379.7 [1967.1–2861.6]), Turkey (2503.3 [2014.3–3054.9]), and Bahrain (2622.3 [2110.9–3205.0]) had the lowest rates (Fig. 4 and Supplementary Table S2). Over the 1990–2019, age-standardized SEV of high-SBP grew by 10.4% (3.8–18.0), from 26.16 (24.0–28.2) to 28.89 (26.9–30.9) (Supplementary Table S5). Except for Libya, Syria, and Yemen, the growth of age-standardized DALY rate attributable to high-SBP in all countries stopped with a negative ARC in the last decade, which was inversely associated with SDI (Fig. 5, Fig. 6 and Supplementary Table S6).

### High fasting plasma glucose

ASDR attributable to high-FPG increased by 21.4% (7.0–37.4), from 118.5 (91.8–162.2) to 143.8 (105.0–195.5). Nonetheless, the annual rate of growth declined from 0.9% for 1990–2010 interval to 0.1% for 2010–19 interval. Age-standardized DALY rate attributable to high-FPG grew by 27.0% (14.3–40.8), from 2624.2 (2154.4–3264.2) to 3332.7 (2650.1–4117.9) (Supplementary Table S2). The all-ages percentage of DALYs attributable to high-FPG was approximately equal for both genders in 2019 (Fig. 2 and Supplementary Table S3). Diabetes and kidney diseases with 72.8% (69.6–76.4) of its age-standardized DALY rate attributed to high-FPG in 2019 was the most affected cause by high-FPG (Fig. 3 and Supplementary Table S4). Afghanistan (5920.6 [4418.6–7702.5]), Qatar (5263.0 [4206.3–6505.2]), and Bahrain (5121.6 [4202.4–6178.2]) had the highest rates of age-standardized DALYs attributable to high-FPG in 2019, whereas Turkey (2074.3 [1625.1–2697.5]), Iran (2511.2 [2017.4–3108.7]), and Kuwait (2556.5 [2005.3–3226.5]) suffered the least in this regard (Fig. 4 and Supplementary Table S2). Age-standardized SEV of high-FPG in the region increased from 7.9 (7.0–8.9) in 1990 to 15.2 (13.7–16.8) in 2019, equivalent to 92.4% (82.8–103.3) growth. ARC in age-standardized DALY rate attributable to high-FPG was negative for all high and high-middle SDI countries (Figs. 5B and 6e and Supplementary Table S6).

### High body-mass index

ASDR attributable to high-BMI rose by 5.1% (−9.0–25.9), from 127.1 (79.2–181.3) to 133.6 (90.0–179.0) with escalation of the annual rate from 0.1% for 1990–2010 to 0.3% for 2010–19. Likewise, age-standardized DALY rate attributable to high-BMI grew by 8.3% (−6.5–28.8), from 3488.8 (2262.5–4798.5) in 1990 to 3777.2 (2692.6–4943.3) in 2019 (Supplementary Table S2). The percentage of DALYs attributable to high-BMI had the same pattern among age groups in both 1990 and 2019, with an increasing trend up to 60–64 age group followed by a decreasing trend in older age groups (Fig. 1 and Supplementary Table S3). In 2019, 53.9% (42.0–64.9) of diabetes and kidney diseases and 32.1% (22.6–41.8) of cardiovascular diseases age-standardized DALY rate was attributed to high-BMI (Fig. 3 and Supplementary Table S4). Egypt (5929.6 [3973.1–8107.8]), the UAE (5732.9 [4111.7–7383.3]), and Afghanistan (5098.6 [3230.9–7244.7]) had the highest age-standardized DALY rates attributable to high-BMI in 2019, while Iran (2580.9 [1845.1–3337.3]), Yemen (2595.6 [1422.7–4079.9]), and Turkey (2662.6 [1804.0–3599.4]) had the lowest rates (Fig. 4 and Supplementary Table S2). From 1990 to 2019, age-standardized SEV of high-BMI increased by 76.0% (58.9–99.3) in the region, from 18.9 (14.3–24.9) to 33.3 (26.4–41.5). The attributable age-standardized DALY rate had been declining in all countries of high and high-middle SDI-quintiles, with ARC negatively associated with SDI (Figs. 5C and 6 and Supplementary Table S6).

### High low-density lipoproteins cholesterol

ASDR attributable to high-LDL decreased by 26.5% (18.6–35.2), from 140.5 (107.6–178.2) to 103.3 (75.3–135.6) at the annual rate of −1.1%. Age-standardized DALY rate attributable to high-LDL also shrank by 30.2% (20.9–39.0), from 3201.1 (2628.8–3822.8) to 2235.4 (1767.8–2767.8) (Supplementary Table S2). The percentage of DALYs attributable to high-LDL had a relative peak at the 55–59 age group (Fig. 1 and Supplementary Table S3). The percentage of DALYs attributable to high-LDL was significantly higher in males from 35 to 39 to 50–54 age groups in 2019 (Fig. 2 and Supplementary Table S3). In 2019, 31.5% (26.0–37.3) of age-standardized DALY rate of cardiovascular diseases was attributed to high-LDL (Fig. 3 and Supplementary Table S4). The leading three countries in terms of age-standardized DALY rates attributable to high-LDL in 2019, were Afghanistan (3927.4 [2902.5–5166.0]), Egypt (3560.6 [2583.8–4814.1]), and Syria (3423.1 [2447.0–4651.2]); conversely, Turkey (1060.8 [782.3–1418.2]), Bahrain (1389.0 [1031.0–1802.7]), and Kuwait (1401.8 [1112.8–1775.6]) were the last three countries in this regard (Fig. 4 and Supplementary Table S2). Age-standardized SEV of high-LDL grew by 5.5% (4.3–7.1) during the 1990–2019 period, from 33.1 (30.1–36.4) to 35.0 (32.0–38.2). Oman (−3.31%), Bahrain (−3.00%), and the UAE (−2.92%) had the fastest annual rate of decline in age-standardized DALY rate attributable to high-LDL over the last decade (Fig. 6 and Supplementary Table S6).

### Gender disparity ratio

**Fig. 7:**
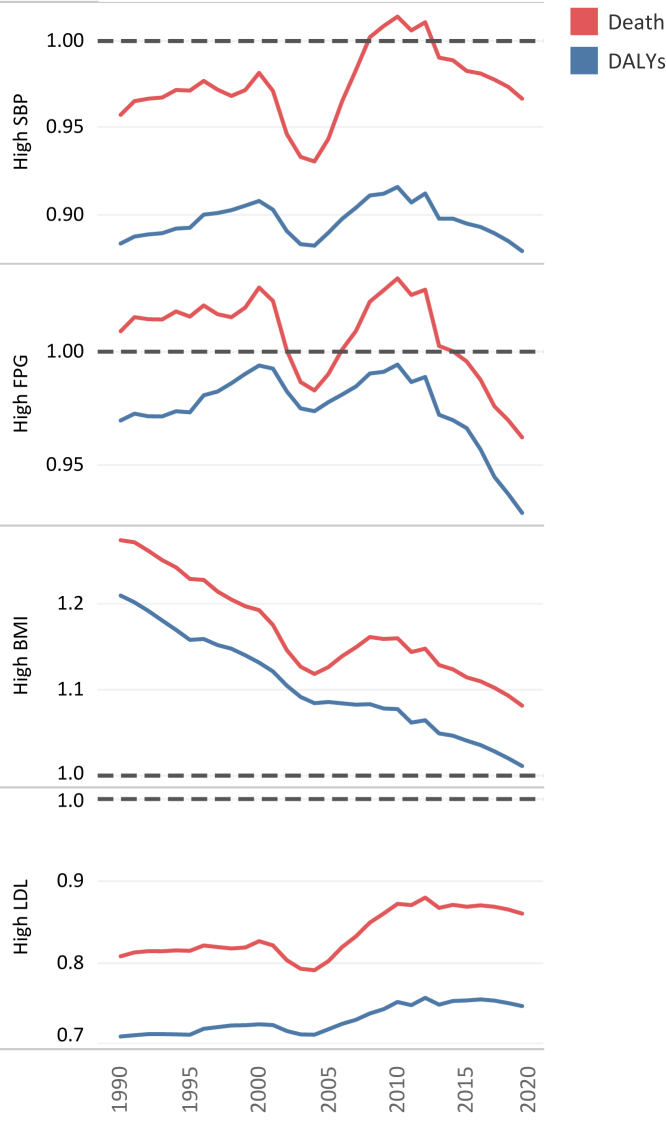
Age-standardized GDR of attributable death and DALY rates trend from 1990 to 2019 for high-SBP, high-FPG, high-BMI, and high-LDL in the North Africa and the Middle East region.

Except for high-BMI with a regressing trend to equality, despite some fluctuations, there were no definite trends in age-standardized GDR of death and DALY rates attributable to high-SBP, high-FPG, and high-LDL. Notably, age-standardized GDR of attributable death rate compared to DALY rate had been consistently deviated toward females in all four risk factors (Fig. 7). However, age-standardized GDR of attributable death and DALY rates were considerably heterogeneous in 2019 among countries in different SDI-quintiles and risk factors (Supplementary Table S7). In 2019, the highest deviation toward females in ASDR for all four risk factors was seen in Qatar with 1.66, 1.62, 1.57, and 1.51 for high-BMI, high-SBP, high-LDL, and high-FPG, respectively. In contrast, Kuwait with 0.42 for high-LDL, and Lebanon with 0.58, 0.60, and 0.73 for high-SBP, high-FPG, and high-BMI, respectively, had the most deviation of ASDR toward males.

## Discussion

Despite the increase in SEVs of high-SBP and high-LDL, death and DALY rates attributable to high-SBP and high-LDL decreased. On the contrary, SEVs of high-FPG and high-BMI and the attributed death and DALY rates moved in the same direction. There was significant diversity among the countries in the region regarding the trend of exposure and attributable burden. There was a negative association between SDI and ARC in age-standardized DALY rate attributable to all four risk factors.

The region experienced a higher prevalence of hypertension than the rest of the world.^3^ About a quarter of total deaths were attributed to high-SBP in the region. Factors contributing to high-SBP have shown unfavorable trends in the past decades. Favourably, there has been decoupling between high-SBP and mortality during the study period. There is evidence that antihypertensive medications result in decreased cardiovascular incidence and mortality,^8^ which could also be reflected in the results of this study. Only three countries, including Libya, Syria, and Yemen, could not stop the growth of DALY rate attributable to high-SBP in the past decade. All three mentioned countries have experienced significant conflicts and war, which have also taken a devastating toll on their healthcare systems infrastructure. Furthermore, not all countries in the region have national policies for high-SBP. Most action plans to address high-SBP focus on enhancing access to antihypertensive treatment and lowering salt consumption. Many countries are gradually reducing the salt content of their highly consumed food, especially bread.^9^ Negligence of high-SBP as a serious health risk factor by the general public is so devastating that, irrespective of their economic development, Yemen and Saudi Arabia had the same low levels of patients' compliance to treatment.^10^^,^^11^

Despite the increase in the death attributable to high-FPG, its annual rate of growth significantly declined in 2010–2019 compared with 1990–2010. Alarmingly, the region has one of the highest prevalence of diabetes in the world.^12^ There is propitious evidence that adherence to antidiabetic medications could reduce the risk of death among patients with diabetes.^13^ However, reports on the quality of care in diabetes are scarce from developing countries.^14^ Afghanistan, with the highest rate of DALYs attributable to high-FPG in 2019, still has no action plan for diabetes response. Yemen ranked among the countries with the highest growth of DALY rate attributable to high-FPG in the last decade, which could be a result of political instability and war.^15^ On the other hand, Qatar, one of the most prosperous countries in the region in reducing the age-standardized DALY rate, has managed to redesign its diabetes prevention via reinforcing lifestyle advice, education, and counseling to patients with diabetes and those at risk.^16^ Turkey, Iran, and Kuwait had the lowest DALY rate due to high-FPG. Turkey, the only country with declining exposure to high-FPG since 2010, started implementing a healthcare system reform program in 2003, which has improved accessibility to the healthcare system and early diagnosis of diabetes.^17^ Iran piloted its first program for the prevention and control of diabetes in 1992 and widely launched it in 2004.^18^ Although the previous efforts have aimed to identify high-risk individuals and improve the quality of diabetes care, the prevalence of diabetes in Iran remains higher than the global average, which calls for prevention strategies and risk factor elimination.^19^ Kuwait has also established a diabetes resource center aiming to provide education to patients as well as to train educators and nutritionists.^20^ There is limited data on the extent of implementation and efficacy of diabetes response programs and guidelines in the region. Nevertheless, evidence suggests that the management of diabetes is suboptimal, as less than half of patients reach target levels in glycemic control.^21^ Until the implementation of improved screening and diabetes care throughout the region, diabetes continues to take a heavy toll, especially considering that some countries in the region face war, political instability, climate change, forced migration, and social instability, which disrupt the delivery of health care and social services.

Worldwide, the prevalence of overweight has outweighed that of underweight in the past decades,^22^ making high-BMI an explicit public health concern. There was a regressing trend in the gender equality for high-BMI in the region during the study period. While females had higher BMI than males in 1975, the gap between genders in the region shrank or even reversed during the following four decades.^23^ However, the gender gap in burden attributable to high-FPG has been widening over the last decade. Given that obesity is a significant driver of type 2 diabetes, the steeper decreasing trend of high-BMI in females can potentially widen the current gender gap in high-FPG. Nevertheless, the prevalence of obesity remains critically high among females in the region. In this sense, cultural factors have traditionally played a significant role.^24^ The prevalence of obesity in high-income countries in the region may be partially attributed to cultural ideals which equate plumpness with beauty, health, and affluence.^25^ Other significant cultural factors contributing to obesity may include certain eating habits such as sharing plates, as well as traditional wide clothing worn by many women, which could hide body fat and thus reduce the motivation to lose weight.^26^ Among Egypt, the UAE, and Afghanistan, which had the highest DALY rates attributable to high-BMI, Afghanistan had no documented national action plan for obesity and high-BMI. In its national nutrition strategy for 2017–2021, the UAE aimed to reduce morbidity and mortality rates due to non-communicable diseases via promoting a healthy diet and physical activity.^27^ While Iran had one of the lowest DALY rates attributable to high-BMI, it has implemented no national policy against obesity so far. Nevertheless, a pilot intervention to promote a healthy lifestyle and targeted education had satisfying results on dietary behaviors and the prevalence of obesity.^28^ In 2010, Turkey introduced a national program to improve knowledge and healthy habits to address the high-BMI burden. It revolved around physical activity, primary care, counseling, modeling healthy living, and structural environmental changes.^29^

Even after the witnessed reduction, the DALYs attributable to high-LDL in the region are still considerably higher than the rest of the world,^3^ possibly due to poor control of dyslipidemia via optimal doses of statins or combination therapies.^30^ In the meantime, we witnessed a decoupling between high-LDL exposure and mortality and morbidity in this study. It could be hypothesised that the reduced mortality is due to increasing use of lipid-lowering medications during the study period.^31^ Statins effectively lower LDL cholesterol concentrations and large multicentre statin trials have conclusively shown that such a reduction decreases both morbidity and mortality and that the reduction in risk is proportional to the absolute reduction in LDL cholesterol. It has been reported that despite treatment with statins, about two-thirds of patients in UAE, Saudi Arabia, Lebanon, and Jordan still had uncontrolled LDL concentrations.^32^ Furthermore, this figure reached 70% in Egypt.^30^ Only Turkey, Qatar, and Iran managed to halt SEV growth of high-LDL in the last decade. The favorable decline of exposure to high-LDL in Iran could be attributable to the widespread prescription of statins by general practitioners^3^ and increased public awareness about dyslipidemia.^33^ Widespread utilization of statins along with increasing number of eligible patients for statin therapy call for an increase in healthcare resources.^34^ This is particularly worrisome, considering the concerning reports from the region indicating poor coverage of lipid-lowering medications among known cases of dyslipidemia.^35^

In developing countries, one of the main challenges of health systems in evidence-based decision-making is the lack of reliable and updated information. It is also noteworthy that despite the favorable trend of some countries in reducing the attributable burden of studied risk factors in the region, there was significant heterogeneity between them. The most notable of these differences were three countries involved in the war, including Syria, Yemen, and Libya, where the age-standardized DALY rate attributable to all four risk factors had a growing trend during the last decade. In contrast, the wealthy Arab States of the Persian Gulf, including Bahrain, Kuwait, Oman, Qatar, Saudi Arabia, and the UAE, had a decreasing trend in age-standardized DALY rate attributable to all four risk factors over the last decade. Such cases clearly show the devastating effects of political and economic instabilities on the health of societies and underscore the necessity of multilateral regional and international cooperation to establish stability in the region. Thus, addressing the MRF calls for joint comprehensive multidisciplinary action plans aiming at bridging the inequality gaps in the region by improving lifestyle, early detection, and healthcare access as well as political and economic stability across countries.

In the past three decades, the burdens attributable to high-LDL and high-SBP decreased; however, they increased for high-BMI and high-FPG. Concerningly, there were increases in exposure to all studied metabolic risk factors in the region. The witnessed decrease in the burden of high-LDL and high-SBP has been attributable to the effectiveness of strategies targeting these risk factors.^36^^,^^37^ In this sense, the development of new treatments for obesity could provide a glimpse of hope in reducing mortality related to high-BMI.^38^

MRF are often interconnected through common pathways that trigger diseases. The global syndemic framework, as emphasized in The Lancet Commission Report, highlights the epidemic synergy between metabolic disease, cardiovascular disease, disability, cancer, and premature death, which share a common pathomechanistic pathway and underlying societal drivers. Previously, MRF were considered individual entities, but consolidating them into a single global syndemic framework serves to address the combined challenges and stresses the importance of prioritizing upstream solutions to mitigate the overall metabolic milieu of the individual.^39^ Addressing the conspicuous burden of MRF requires holistic views and deep understanding of their modifiable and unmodifiable underlying sources. A range of evidence-based strategies for improved diet quality, physical activity, screening, pharmacological interventions, and access to care are needed. In this sense, policymakers should focus on the upstream determinants of health rather than targeting the individual metabolic entity, particularly as these issues may arise from a range of factors that vary by geography, socioeconomic status, and gender.

This study presents estimates for the burden of four MRF, including high-SBP, high-FPG, high-BMI, and high-LDL, based on the data of the GBD 2019. While several metabolic variables were included in this study, other metrics related to the definition of metabolic syndrome, such as waist circumference, post-prandial plasma glucose or glycated hemoglobin, triglycerides and HDL were not included in the dataset. The study provides the opportunity to investigate the current situation and trends of health metrics and measures in the region during the past three decades. Nevertheless, the overall quality of the GBD estimates fundamentally relies on the accuracy of data sources used in the modeling. Although there are nationally representative surveys among the countries in the region, such as the WHO STEPwise Approach to NCD Risk Factor Surveillance (STEPS) or the National and Subnational Burden of Diseases, Injuries, and Risk Factors (NASBOD) Study, the reliability of registry data among countries with limited resources in the region could be questionable. Moreover, countries were heterogeneous in the number of included data sources, with resource-constraint countries having fewer data sources. Thus, the GBD includes various modeling processes to overcome this limitation and presents metrics with 95% UIs.

The burden attributed to high-SBP and high-LDL decreased during the 1990–2019 period in the region, while the attributable burden of high-FPG and high-BMI increased. Alarmingly, exposure to all four risk factors increased in the past three decades. There has been significant heterogeneity among the countries in the region regarding the trends of exposure and attributable burden. The increasing trend of all-ages morbidity and mortality attributable to metabolic risk factors indicates an ongoing epidemiological transition from communicable to non-communicable diseases in NAME. To this end, urgent action is required at the individual, community, and national levels in terms of introducing effective strategies for prevention and treatment that account for local and socioeconomic factors. Vigorous preventions program with comprehensive surveillance and monitoring, as well as joint collaborations between countries of the region, are urgently needed to improve metabolic health.

## Contributors

Please see the appendix file for more detailed information about individual author contributions to the research, divided into the following categories: providing data or critical feedback on data sources; developing methods or computational machinery; providing critical feedback on methods or results; drafting the manuscript or revising it critically for important intellectual content; and management of the overall research enterprise. Members of the core research team for this topic area had full access to the underlying data used to generate estimates presented in this article. All other authors had access to and reviewed estimates as part of the research evaluation process, which includes additional stages of formal review.

## Data sharing statement

This study follows the Guidelines for Accurate and Transparent Health Estimates Reporting. All data sources used in this analysis are found on the Global Health Data Exchange (http://ghdx.healthdata.org/gbd-2019/data-input-sources), and related code is available at http://ghdx.healthdata.org/gbd-2019/code. Additional results from this study and the larger GBD 2019 analysis can be explored using our data visualization tools at https://vizhub.healthdata.org/gbd-compare.

## Declaration of interests

Z Aryan acknowledges support for attending meetings and/or travel from the American Heart Association Travel Grant. A Hassan acknowledges support from consulting fees from Novartis, Sanofi Genzyme, Biologix, Merck, Hikma Pharma, Janssen, Inspire Pharma, Future Pharma, Elixir pharma; payment or honoraria for lectures, presentations, speakers bureaus, manuscript writing or educational events from Novartis, Allergan, Merck, Biologix, Janssen, Roche, Sanofi Genzyme, Bayer, Hikma Pharma, Al Andalus, Chemipharm, Lundbeck, Inspire Pharma, Future Pharma and Habib Scientific Office, and Everpharma; support for attending meetings and/or travel from Novartis, Allergan, Merck, Biologix, Roche, Sanofi Genzyme, Bayer, Hikma Pharma, Chemipharm, and Al Andalus and Clavita pharm; all outside the submitted work. All other authors had no competing interest to declare.

## Appendix A Supplementary data

Supplementary Tables S2–S7

Affiliations

Contributions

PubMed_Table

Caption for Supplementary material
