# Supplementary material for: The burden of metabolic risk factors in North Africa and the Middle East, 1990–2019: findings from the Global Burden of Disease Study
Source: eClinicalMedicine. 2023 Jun 2;60:102022. doi: 10.1016/j.eclinm.2023.102022 (PMC10242634; doi:10.1016/j.eclinm.2023.102022)
Supplement: Supplementary Tables S2–S7 [file mmc2.pdf]

Supplementary Table 2

Risk factor	Measure	Year	The NAME	Afghanistan	Algeria	Bahrain	Egypt	Iran	Iraq	Jordan	Kuwait	Lebanon	Libya
High Systolic Blood Pressure	Death Rate	1990	286.51 (246.73-323.37)	405.00 (319.87-490.75)	393.00 (315.83-472.60)	332.42 (273.24-394.55)	309.71 (262.26-358.72)	244.79 (209.54-279.57)	331.99 (275.15-396.07)	276.37 (231.66-323.60)	182.77 (156.19-206.30)	259.43 (209.18-317.50)	200.50 (160.45-243.25)
		2010	238.51 (205.99-268.92)	363.79 (281.47-454.28)	256.37 (206.79-304.00)	191.68 (157.33-228.76)	335.97 (284.76-387.97)	173.99 (148.96-196.47)	311.49 (249.06-376.94)	187.75 (159.23-214.03)	138.63 (119.66-154.75)	204.52 (154.34-240.14)	181.82 (155.79-206.73)
		2019	219.41 (185.61-252.54)	341.80 (262.19-421.64)	235.45 (185.57-286.97)	147.77 (115.60-184.64)	314.94 (238.39-403.48)	157.77 (135.35-179.10)	296.57 (238.44-350.01)	168.49 (137.79-199.52)	115.25 (92.91-138.66)	191.32 (138.58-228.38)	188.73 (146.12-232.31)
	DALY Rate	1990	5,887.94 (5,221.21-6,582.29)	9,186.51 (7,100.08-11,432.12)	6,958.67 (5,663.23-8,314.93)	6,431.56 (5,370.19-7,560.64)	6,314.85 (5,392.76-7,203.58)	4,800.22 (4,200.47-5,423.47)	7,130.70 (5,973.39-8,436.63)	5,417.26 (4,592.26-6,301.38)	3,748.24 (3,277.46-4,207.51)	5,295.50 (4,364.89-6,438.28)	4,212.26 (3,440.24-5,106.86)
		2010	4,808.89 (4,276.12-5,361.24)	8,061.58 (6,163.15-10,331.32)	4,294.65 (3,600.02-5,035.84)	3,330.95 (2,820.71-3,894.79)	7,037.59 (6,071.49-7,975.89)	3,299.30 (2,949.68-3,645.97)	6,545.79 (5,241.14-7,970.70)	3,557.55 (3,095.11-4,014.80)	2,885.46 (2,584.24-3,167.06)	4,020.15 (3,207.53-4,642.88)	3,880.79 (3,483.16-4,355.29)
		2019	4,401.89 (3,785.93-5,042.84)	7,400.80 (5,554.91-9,271.56)	3,927.64 (3,132.49-4,789.98)	2,622.29 (2,110.86-3,205.01)	6,576.09 (4,975.86-8,308.12)	2,973.39 (2,652.25-3,280.15)	6,025.44 (4,796.31-7,308.90)	3,228.75 (2,707.60-3,804.52)	2,379.70 (1,967.15-2,861.56)	3,904.36 (2,956.72-4,605.25)	4,061.98 (3,223.62-5,040.70)
High Fasting Plasma Glucose	Death Rate	1990	118.49 (91.84-162.16)	183.29 (132.26-259.34)	145.92 (100.02-217.50)	309.84 (225.98-418.76)	108.08 (81.86-154.51)	91.37 (68.37-127.32)	178.78 (136.85-236.21)	187.89 (150.57-245.48)	129.18 (96.68-172.51)	138.16 (94.00-203.86)	111.73 (77.58-164.34)
		2010	141.93 (104.95-194.19)	246.56 (177.55-340.06)	161.48 (107.38-239.17)	282.89 (234.92-338.94)	163.36 (119.41-227.72)	101.80 (74.07-145.12)	206.74 (149.40-275.98)	145.26 (116.41-182.11)	100.63 (77.62-129.04)	147.77 (95.55-206.38)	123.56 (90.58-172.39)
		2019	143.83 (105.00-195.54)	245.47 (174.13-335.05)	156.69 (104.10-226.29)	233.64 (185.85-286.26)	193.70 (127.29-273.85)	109.54 (80.75-150.69)	206.00 (152.78-266.93)	127.64 (99.48-162.46)	86.91 (64.98-114.92)	140.72 (92.59-197.38)	131.09 (90.90-187.90)
	DALY Rate	1990	2,624.17 (2,154.43-3,264.22)	4,314.30 (3,209.10-5,668.91)	2,798.12 (2,115.15-3,800.29)	6,261.77 (4,891.49-7,982.59)	2,311.51 (1,894.29-2,922.03)	1,988.06 (1,601.44-2,516.03)	4,212.40 (3,364.83-5,303.99)	3,973.00 (3,269.12-4,986.94)	3,043.66 (2,430.85-3,770.02)	2,928.58 (2,190.04-3,918.11)	2,532.90 (1,913.83-3,400.88)
		2010	3,180.34 (2,564.96-3,943.35)	5,862.93 (4,450.70-7,687.30)	3,129.04 (2,381.11-4,116.83)	5,794.30 (4,973.47-6,720.59)	3,600.40 (2,835.41-4,550.78)	2,275.79 (1,826.58-2,847.25)	4,763.79 (3,644.07-6,151.66)	3,276.10 (2,739.91-3,919.23)	2,764.69 (2,243.27-3,357.74)	3,257.65 (2,400.26-4,220.40)	3,159.15 (2,487.46-3,961.21)
		2019	3,332.73 (2,650.14-4,117.87)	5,920.63 (4,418.61-7,702.53)	3,206.25 (2,476.21-4,144.79)	5,121.64 (4,202.36-6,178.17)	4,369.04 (3,084.35-5,760.58)	2,511.20 (2,017.38-3,108.71)	4,737.92 (3,650.18-5,900.26)	2,960.09 (2,379.65-3,643.72)	2,556.53 (2,005.34-3,226.50)	3,311.63 (2,448.91-4,266.77)	3,462.02 (2,627.86-4,581.71)
High Body-Mass Index	Death Rate	1990	127.14 (79.24-181.31)	132.74 (63.89-217.10)	131.80 (75.35-203.19)	211.59 (136.09-293.01)	173.76 (107.28-248.55)	89.20 (52.92-131.73)	194.46 (119.31-272.09)	178.39 (114.87-244.36)	122.15 (81.34-161.83)	128.15 (75.90-185.67)	106.38 (67.50-150.13)
		2010	129.56 (86.69-174.76)	145.02 (77.54-227.08)	124.19 (78.21-176.07)	186.17 (124.67-245.39)	209.00 (137.64-280.82)	87.01 (58.15-119.19)	173.82 (105.23-250.12)	144.87 (99.86-190.46)	102.19 (69.71-133.57)	117.27 (72.45-163.41)	119.43 (82.07-156.36)
		2019	133.59 (90.04-179.03)	177.28 (109.10-256.14)	125.19 (80.29-177.33)	161.71 (107.07-219.51)	217.70 (139.98-307.80)	91.72 (63.92-122.14)	172.01 (111.15-237.23)	137.12 (94.26-183.36)	93.59 (62.39-125.36)	120.60 (77.64-166.42)	127.00 (83.09-174.56)
	DALY Rate	1990	3,488.84 (2,262.50-4,798.46)	3,923.72 (1,946.79-6,345.43)	3,481.36 (2,158.88-5,095.66)	5,453.51 (3,688.71-7,355.30)	4,648.23 (2,981.32-6,435.55)	2,419.58 (1,497.57-3,414.66)	5,512.65 (3,548.13-7,546.62)	4,595.67 (3,077.04-6,088.89)	3,581.48 (2,514.78-4,602.40)	3,499.35 (2,176.73-5,023.44)	3,132.16 (2,088.79-4,318.24)
		2010	3,639.48 (2,547.89-4,768.69)	4,240.20 (2,347.27-6,524.12)	3,254.17 (2,219.07-4,450.55)	4,661.13 (3,275.85-6,018.30)	5,650.72 (3,858.81-7,324.49)	2,430.19 (1,699.18-3,194.78)	4,987.60 (3,269.05-6,981.13)	3,870.19 (2,750.14-4,863.03)	3,313.05 (2,415.14-4,163.38)	3,273.75 (2,151.56-4,456.37)	3,675.79 (2,617.68-4,683.38)
		2019	3,777.18 (2,692.63-4,943.28)	5,098.61 (3,230.86-7,244.71)	3,339.17 (2,312.75-4,478.52)	4,297.40 (2,997.09-5,612.61)	5,929.58 (3,973.10-8,107.81)	2,580.92 (1,845.13-3,337.35)	4,793.11 (3,232.66-6,505.33)	3,701.63 (2,637.00-4,819.42)	3,156.08 (2,248.53-4,046.22)	3,488.60 (2,344.55-4,641.18)	3,920.96 (2,681.32-5,191.76)
High LDL Cholesterol	Death Rate	1990	140.51 (107.63-178.22)	192.07 (140.06-250.48)	165.91 (113.87-228.45)	222.45 (168.51-280.92)	170.76 (126.69-219.27)	145.54 (109.31-188.81)	146.68 (109.67-191.03)	122.79 (90.00-163.48)	98.67 (77.15-123.75)	168.35 (125.89-216.78)	81.33 (56.94-113.86)
		2010	111.48 (83.43-143.04)	172.44 (125.68-235.65)	113.02 (75.10-155.05)	102.48 (72.24-136.01)	165.04 (124.49-210.59)	88.11 (64.80-115.64)	134.74 (95.84-182.39)	76.55 (56.65-101.72)	69.45 (54.53-87.67)	128.65 (88.81-166.36)	78.51 (57.85-104.09)
		2019	103.30 (75.32-135.56)	160.23 (116.09-211.53)	105.28 (67.09-149.03)	76.59 (50.60-106.10)	159.60 (111.27-220.26)	79.86 (58.17-105.14)	126.29 (89.61-169.18)	68.94 (49.25-95.26)	59.84 (44.14-79.44)	120.59 (80.71-156.38)	84.36 (58.69-117.73)
	DALY Rate	1990	3,201.08 (2,628.85-3,822.83)	4,947.80 (3,651.43-6,468.07)	3,265.44 (2,429.31-4,293.63)	4,582.30 (3,714.99-5,486.79)	3,826.30 (3,103.43-4,725.73)	3,106.58 (2,528.43-3,789.11)	3,372.76 (2,653.41-4,220.17)	2,664.63 (2,109.52-3,337.76)	2,292.02 (1,934.68-2,684.93)	3,727.72 (2,991.30-4,545.03)	1,878.22 (1,414.51-2,511.50)
		2010	2,433.09 (1,987.76-2,917.64)	4,347.00 (3,238.59-5,780.43)	2,094.39 (1,557.58-2,727.91)	1,827.82 (1,436.33-2,262.59)	3,700.47 (2,967.13-4,467.89)	1,769.16 (1,432.16-2,147.46)	2,991.01 (2,198.60-3,899.24)	1,588.95 (1,297.43-1,975.75)	1,656.53 (1,410.96-1,951.23)	2,628.52 (1,983.27-3,210.97)	1,786.46 (1,449.16-2,308.17)
		2019	2,235.44 (1,767.81-2,767.81)	3,927.38 (2,902.51-5,165.97)	1,913.98 (1,362.60-2,580.77)	1,388.97 (1,030.97-1,802.72)	3,560.60 (2,583.81-4,814.10)	1,574.46 (1,268.65-1,922.44)	2,668.82 (1,966.51-3,511.53)	1,438.72 (1,109.08-1,845.95)	1,401.78 (1,112.85-1,775.58)	2,537.48 (1,828.55-3,159.21)	1,947.06 (1,443.96-2,622.98)

Supplementary Table 2

Risk factor	Measure	Year	Morocco	Oman	Palestine	Qatar	Saudi Arabia	Sudan	Syria	The UAE	Tunisia	Turkey	Yemen
High Systolic Blood Pressure	Death Rate	1990	339.09 (290.15-384.19)	287.49 (215.20-371.19)	277.42 (216.76-343.37)	310.20 (245.23-372.16)	233.19 (184.00-287.93)	378.35 (308.70-454.67)	309.98 (239.36-379.49)	381.64 (317.13-461.82)	210.75 (173.41-251.81)	227.16 (189.37-261.90)	320.82 (250.15-401.62)
		2010	298.73 (241.05-363.71)	331.25 (289.00-373.71)	223.68 (191.42-253.28)	245.89 (198.01-294.91)	253.81 (218.78-289.51)	331.03 (255.85-406.64)	266.85 (208.21-329.29)	317.43 (259.68-376.00)	191.33 (142.18-244.44)	158.44 (133.00-183.23)	277.01 (219.10-344.21)
		2019	307.72 (240.01-359.26)	274.45 (225.06-326.83)	211.60 (174.54-252.94)	206.71 (155.46-261.91)	209.61 (167.63-250.73)	317.98 (259.92-391.57)	265.64 (195.69-340.18)	215.26 (168.17-266.16)	175.52 (128.07-227.82)	133.60 (104.79-165.29)	280.08 (216.95-353.98)
	DALY Rate	1990	6,962.00 (6,017.25-7,928.41)	5,912.22 (4,456.83-7,656.70)	5,499.91 (4,290.15-6,812.75)	5,707.93 (4,698.82-6,810.11)	4,907.21 (3,876.33-6,131.36)	8,206.84 (6,668.87-9,926.50)	6,561.11 (5,182.54-8,043.41)	7,550.86 (6,332.89-9,072.38)	4,013.30 (3,322.98-4,753.19)	4,667.00 (3,957.08-5,360.97)	6,804.89 (5,235.46-8,710.56)
		2010	6,083.74 (4,889.60-7,443.90)	6,675.34 (5,958.43-7,436.20)	4,263.02 (3,686.56-4,754.87)	4,180.25 (3,460.81-4,940.96)	5,449.85 (4,772.42-6,157.17)	6,925.49 (5,292.95-8,658.11)	5,050.78 (4,005.42-6,143.09)	5,990.06 (4,943.52-7,005.66)	3,591.73 (2,696.00-4,555.20)	2,964.07 (2,581.02-3,404.69)	5,682.44 (4,434.46-7,127.37)
		2019	5,955.25 (4,659.10-7,044.06)	4,921.40 (4,161.25-5,715.35)	3,963.97 (3,318.58-4,648.75)	3,215.68 (2,498.00-4,061.58)	4,430.78 (3,521.40-5,282.85)	6,465.28 (5,167.47-8,161.93)	5,126.62 (3,761.21-6,668.83)	4,415.11 (3,446.46-5,592.66)	3,312.73 (2,472.38-4,317.64)	2,503.26 (2,014.27-3,054.89)	5,737.53 (4,412.14-7,387.13)
High Fasting Plasma Glucose	Death Rate	1990	100.26 (74.62-141.92)	178.08 (126.76-252.49)	200.45 (141.68-274.75)	324.74 (248.18-426.26)	154.27 (113.13-211.02)	122.33 (83.09-182.55)	143.67 (101.33-214.96)	274.84 (214.85-361.26)	115.58 (77.57-174.03)	109.73 (89.20-136.72)	91.84 (66.58-125.33)
		2010	161.67 (109.63-237.22)	259.03 (193.59-347.88)	204.42 (159.08-260.22)	323.27 (262.25-398.09)	173.71 (136.00-223.32)	165.87 (109.38-246.95)	189.18 (119.71-287.11)	269.19 (210.85-339.51)	137.76 (87.02-201.13)	107.23 (80.68-143.09)	125.06 (86.24-184.04)
		2019	187.15 (128.45-266.80)	232.96 (169.06-315.27)	216.83 (168.32-286.17)	266.41 (200.56-345.47)	148.29 (112.06-194.94)	172.54 (120.65-252.16)	197.04 (122.77-295.75)	179.81 (134.63-230.47)	131.82 (84.52-193.66)	88.58 (63.57-124.55)	138.27 (92.18-205.05)
	DALY Rate	1990	2,244.02 (1,773.67-2,896.05)	3,857.71 (2,897.54-5,160.15)	4,163.64 (3,091.55-5,454.76)	6,355.16 (5,097.32-7,994.86)	3,388.18 (2,589.98-4,421.44)	2,619.53 (1,949.72-3,536.12)	3,110.57 (2,379.98-4,199.84)	5,606.20 (4,484.39-7,171.96)	2,311.07 (1,760.94-3,060.18)	2,583.67 (2,153.35-3,051.14)	2,071.79 (1,569.68-2,749.24)
		2010	3,547.02 (2,639.70-4,772.42)	5,289.86 (4,236.28-6,690.63)	4,373.08 (3,568.26-5,337.65)	6,566.25 (5,449.27-7,841.13)	4,118.76 (3,351.30-5,023.03)	3,593.21 (2,589.89-4,909.34)	3,667.34 (2,686.28-4,982.10)	5,833.24 (4,735.98-7,151.64)	2,976.16 (2,166.45-3,870.70)	2,433.29 (1,969.20-3,026.56)	2,688.65 (1,989.42-3,726.52)
		2019	4,049.56 (3,046.61-5,338.12)	4,620.40 (3,618.47-5,820.13)	4,667.61 (3,783.93-5,803.84)	5,263.04 (4,206.33-6,505.17)	3,765.48 (2,970.80-4,712.30)	3,837.30 (2,905.38-5,098.16)	4,015.89 (2,858.59-5,556.49)	4,416.55 (3,464.78-5,546.02)	3,023.13 (2,197.82-3,972.13)	2,074.26 (1,625.08-2,697.53)	3,045.57 (2,152.01-4,267.22)
High Body-Mass Index	Death Rate	1990	107.09 (62.37-161.45)	114.05 (59.98-182.32)	122.16 (63.49-197.33)	229.42 (146.98-313.14)	121.56 (73.55-178.67)	94.07 (47.20-152.96)	141.86 (80.88-214.42)	224.55 (151.96-304.59)	89.59 (52.86-133.83)	127.59 (79.46-179.54)	64.31 (24.53-120.13)
		2010	127.82 (77.17-186.53)	207.50 (140.58-275.30)	114.75 (66.19-168.60)	252.00 (174.39-328.35)	177.94 (122.29-231.58)	118.37 (68.19-174.69)	138.02 (84.36-198.17)	280.45 (195.51-363.37)	101.20 (60.35-151.76)	99.25 (63.95-137.51)	79.58 (39.32-129.16)
		2019	145.30 (89.56-207.79)	177.53 (119.00-237.42)	131.57 (80.88-190.90)	209.62 (137.12-285.72)	160.71 (109.22-212.06)	136.02 (82.69-197.59)	143.80 (85.57-216.72)	203.03 (138.40-274.58)	101.68 (60.27-153.55)	95.18 (59.46-136.74)	88.64 (45.87-143.46)
	DALY Rate	1990	2,978.64 (1,810.24-4,339.53)	3,177.47 (1,788.46-4,868.13)	3,419.11 (1,948.04-5,287.67)	5,514.94 (3,644.22-7,352.24)	3,486.02 (2,198.78-4,998.60)	2,760.80 (1,444.99-4,319.65)	4,102.09 (2,441.39-6,045.96)	5,877.80 (4,059.13-7,909.40)	2,450.44 (1,547.96-3,540.63)	3,470.25 (2,224.29-4,783.75)	1,839.85 (730.39-3,329.78)
		2010	3,562.54 (2,235.65-5,061.28)	5,345.37 (3,706.28-6,909.80)	3,262.10 (2,078.87-4,511.74)	5,946.26 (4,265.26-7,576.69)	5,101.59 (3,600.76-6,468.37)	3,454.64 (2,113.19-4,993.56)	3,768.26 (2,438.65-5,274.67)	7,043.48 (5,199.40-8,937.18)	2,824.99 (1,822.48-4,050.59)	2,754.51 (1,847.67-3,675.65)	2,317.76 (1,232.69-3,625.23)
		2019	3,930.33 (2,543.03-5,491.46)	4,401.68 (3,087.83-5,712.61)	3,647.36 (2,444.55-4,973.12)	4,904.93 (3,498.19-6,432.22)	4,771.54 (3,389.16-6,141.76)	3,862.43 (2,483.09-5,500.04)	3,994.50 (2,515.44-5,694.32)	5,732.92 (4,111.71-7,383.33)	2,916.79 (1,907.41-4,177.20)	2,662.62 (1,803.98-3,599.41)	2,595.64 (1,422.71-4,079.92)
High LDL Cholesterol	Death Rate	1990	143.02 (106.19-183.85)	215.42 (154.27-289.10)	146.77 (103.95-203.02)	167.81 (114.90-223.38)	99.25 (67.91-136.90)	142.23 (97.82-192.77)	183.08 (135.65-241.78)	156.44 (111.80-214.74)	109.85 (78.74-145.76)	109.49 (84.45-140.22)	161.19 (111.93-215.59)
		2010	127.56 (89.96-174.11)	191.05 (144.54-240.05)	107.43 (81.06-139.75)	119.29 (81.14-163.40)	122.15 (95.29-155.09)	119.37 (79.12-166.72)	170.38 (121.51-226.51)	148.44 (107.16-202.52)	102.65 (65.85-142.85)	67.22 (47.79-90.79)	138.31 (95.45-189.93)
		2019	135.35 (93.48-180.40)	163.89 (115.65-216.30)	107.97 (77.87-144.63)	102.07 (61.43-148.28)	102.99 (73.19-136.88)	118.01 (79.84-161.73)	167.54 (112.52-231.34)	103.08 (71.43-143.17)	94.29 (61.55-134.54)	53.34 (35.62-76.29)	142.72 (99.97-196.32)
	DALY Rate	1990	3,213.52 (2,516.15-3,936.70)	4,655.64 (3,433.03-6,016.60)	3,178.25 (2,375.10-4,138.45)	3,263.80 (2,441.17-4,198.31)	2,244.31 (1,588.10-2,973.36)	3,419.05 (2,474.54-4,565.90)	4,243.11 (3,268.50-5,375.89)	3,227.60 (2,426.83-4,261.65)	2,225.12 (1,719.34-2,815.84)	2,572.63 (2,097.50-3,106.08)	3,857.75 (2,723.23-5,118.72)
		2010	2,820.38 (2,068.42-3,725.70)	3,964.21 (3,256.96-4,818.33)	2,231.43 (1,817.03-2,735.27)	2,042.97 (1,497.85-2,694.03)	2,885.44 (2,362.76-3,463.67)	2,756.83 (1,870.78-3,754.85)	3,393.32 (2,640.05-4,273.04)	2,937.20 (2,227.38-3,844.29)	2,006.74 (1,390.93-2,710.77)	1,338.64 (1,053.22-1,667.77)	3,117.18 (2,218.97-4,166.19)
		2019	2,806.21 (2,010.30-3,595.89)	2,927.65 (2,253.54-3,705.08)	2,179.07 (1,696.39-2,806.35)	1,579.98 (1,069.87-2,162.70)	2,406.26 (1,815.69-3,037.56)	2,633.10 (1,788.48-3,623.92)	3,423.14 (2,446.97-4,651.23)	2,249.97 (1,645.74-3,008.72)	1,846.97 (1,270.37-2,541.34)	1,060.78 (782.26-1,418.21)	3,219.57 (2,322.08-4,394.93)

Supplementary Table 3

Risk factor	Measure	Year	Sex	Under 20	20 to 24	25 to 29	30 to 34	35 to 39	40 to 44	45 to 49	50 to 54	55 to 59	60 to 64	65 to 69	70 to 74	75 to 79	80 to 84	85 to 89	90 to 94	95 plus
High Systolic Blood Pressure	Deaths	1990	Male	0.01% (0.01-0.02)	0.29% (0.18-0.48)	6.49% (4.37-8.82)	9.78% (6.79-12.89)	13.34% (9.88-17.32)	17.82% (13.56-22.08)	23.74% (19.03-28.67)	27.42% (22.90-31.85)	30.40% (26.07-34.89)	30.95% (26.65-35.34)	31.05% (25.86-35.57)	30.67% (25.38-35.35)	31.78% (26.93-36.82)	28.76% (22.20-35.48)	28.05% (21.29-34.55)	28.06% (20.90-35.17)	28.08% (19.88-35.38)
			Female	0.02% (0.01-0.03)	0.62% (0.35-0.92)	6.89% (4.39-9.73)	9.80% (6.74-12.92)	12.56% (9.23-16.15)	17.34% (13.21-21.71)	24.94% (20.25-29.54)	29.75% (25.01-34.50)	33.11% (28.39-37.75)	36.08% (30.99-41.23)	36.87% (31.06-42.25)	37.63% (31.15-43.62)	37.96% (32.04-43.93)	35.11% (27.34-43.01)	34.03% (26.18-41.48)	33.98% (24.99-41.73)	33.34% (23.58-42.20)
			Both	0.02% (0.01-0.02)	0.41% (0.26-0.57)	6.65% (4.66-8.86)	9.79% (7.09-12.37)	13.02% (9.92-16.08)	17.63% (13.95-21.28)	24.21% (20.07-28.75)	28.32% (24.17-32.21)	31.42% (27.56-35.35)	32.93% (28.94-36.92)	33.41% (28.78-37.92)	33.71% (28.64-38.37)	34.74% (29.84-39.48)	32.07% (25.07-39.13)	31.32% (24.06-37.91)	31.37% (23.58-38.33)	31.30% (22.42-38.90)
		2019	Male	0.04% (0.02-0.07)	0.36% (0.22-0.58)	7.51% (5.35-9.95)	10.32% (7.81-13.38)	13.91% (10.98-17.55)	18.52% (14.79-23.00)	24.63% (20.42-29.31)	28.67% (24.59-33.19)	31.19% (27.23-35.23)	32.27% (27.97-36.59)	32.46% (27.45-36.58)	30.79% (26.03-35.19)	31.93% (27.29-36.66)	29.02% (22.52-35.40)	29.00% (22.28-35.59)	28.74% (21.31-35.39)	28.31% (20.42-35.50)
			Female	0.05% (0.03-0.06)	0.70% (0.46-0.94)	7.90% (5.19-10.58)	10.54% (7.41-13.83)	13.46% (9.99-17.11)	17.34% (13.54-20.97)	23.64% (19.20-27.82)	28.13% (23.75-32.01)	32.21% (27.74-36.21)	35.94% (30.80-40.12)	37.45% (31.55-42.05)	36.73% (31.14-42.01)	37.65% (31.97-42.69)	35.01% (27.43-41.94)	34.86% (27.12-42.75)	33.81% (24.79-42.03)	32.46% (21.72-40.59)
			Both	0.04% (0.03-0.07)	0.47% (0.32-0.66)	7.65% (5.63-9.85)	10.40% (7.95-13.01)	13.75% (11.11-16.77)	18.08% (14.54-21.60)	24.25% (20.47-28.51)	28.47% (24.64-32.22)	31.58% (27.90-35.18)	33.71% (29.71-37.31)	34.55% (29.68-38.52)	33.39% (28.80-37.42)	34.65% (29.93-39.02)	32.03% (25.23-38.63)	32.01% (24.97-38.85)	31.19% (22.91-38.36)	30.44% (21.43-37.81)
	Disability-Adjusted Life-Years	1990	Male	0.01% (0.01-0.02)	0.22% (0.14-0.34)	4.25% (2.90-5.79)	6.16% (4.21-8.21)	8.61% (6.29-11.22)	12.10% (9.29-15.16)	17.32% (13.69-21.16)	21.34% (17.64-25.16)	24.66% (20.89-28.49)	25.89% (22.09-29.64)	26.31% (21.85-30.29)	26.32% (21.96-30.50)	27.51% (23.36-32.14)	24.94% (19.32-30.80)	24.21% (18.54-29.96)	24.15% (17.99-30.22)	24.13% (17.38-30.42)
			Female	0.02% (0.01-0.02)	0.32% (0.19-0.45)	3.29% (2.05-4.72)	4.54% (3.04-6.23)	6.15% (4.43-8.14)	8.93% (6.52-11.72)	14.37% (11.31-17.44)	19.15% (15.66-23.00)	23.00% (19.45-26.87)	27.14% (23.04-31.58)	28.90% (24.00-33.67)	30.66% (25.39-35.76)	31.76% (26.78-36.87)	29.74% (23.50-36.37)	28.82% (22.38-35.56)	28.66% (21.33-35.19)	28.12% (20.19-35.22)
			Both	0.01% (0.01-0.02)	0.26% (0.18-0.36)	3.78% (2.62-5.04)	5.36% (3.81-6.96)	7.40% (5.57-9.38)	10.61% (8.26-13.14)	15.96% (12.87-19.05)	20.37% (17.17-23.80)	23.97% (20.59-27.44)	26.40% (22.96-29.99)	27.42% (23.49-31.36)	28.28% (24.05-32.28)	29.58% (25.50-33.93)	27.47% (21.69-33.44)	26.75% (20.73-32.39)	26.70% (20.22-32.74)	26.59% (19.13-33.08)
		2019	Male	0.04% (0.02-0.06)	0.27% (0.17-0.41)	4.42% (3.13-5.86)	5.69% (4.16-7.45)	7.62% (5.74-10.08)	10.85% (8.39-13.83)	15.75% (12.76-19.28)	20.03% (16.70-23.58)	23.10% (19.80-26.43)	25.07% (21.46-28.63)	25.92% (21.89-29.71)	25.21% (21.26-29.11)	26.67% (22.69-30.82)	24.29% (18.86-29.63)	24.49% (18.83-30.03)	24.34% (18.06-30.07)	24.08% (17.50-30.06)
			Female	0.04% (0.03-0.05)	0.30% (0.21-0.42)	2.86% (1.84-4.15)	3.70% (2.49-5.10)	4.96% (3.53-6.67)	7.23% (5.36-9.20)	11.45% (8.91-14.23)	15.58% (12.60-18.64)	19.86% (16.41-23.48)	24.44% (20.49-28.14)	27.24% (22.49-31.26)	28.18% (23.49-32.88)	30.24% (25.63-34.52)	28.70% (22.53-34.43)	29.25% (22.74-35.89)	28.09% (20.75-34.54)	26.81% (18.11-33.49)
			Both	0.04% (0.03-0.05)	0.28% (0.20-0.39)	3.67% (2.63-4.85)	4.72% (3.47-6.19)	6.32% (4.78-8.23)	9.13% (7.11-11.51)	13.77% (11.13-16.92)	18.05% (15.04-21.07)	21.69% (18.66-24.78)	24.80% (21.33-27.99)	26.51% (22.48-30.18)	26.56% (22.77-30.19)	28.41% (24.40-32.29)	26.52% (21.04-31.88)	26.93% (21.17-32.58)	26.17% (19.44-32.03)	25.50% (18.20-31.43)
High Fasting Plasma Glucose	Deaths	1990	Male	0.07% (0.06-0.09)	0.30% (0.24-0.38)	1.46% (0.96-2.25)	2.32% (1.55-3.44)	3.27% (2.38-4.53)	4.55% (3.32-6.21)	6.57% (4.91-8.75)	8.44% (6.58-10.94)	10.10% (7.98-12.51)	11.32% (8.74-14.16)	12.30% (8.81-16.58)	13.35% (9.57-18.24)	14.26% (10.53-20.23)	13.44% (8.42-22.11)	12.46% (7.91-20.31)	11.57% (7.40-18.58)	10.74% (6.84-16.93)
			Female	0.11% (0.09-0.14)	1.04% (0.85-1.28)	2.11% (1.54-2.86)	3.03% (2.30-4.06)	3.82% (3.02-4.81)	5.20% (4.18-6.54)	7.87% (6.32-9.77)	10.46% (8.61-12.60)	12.86% (10.57-15.46)	14.87% (11.93-18.26)	15.67% (11.94-20.40)	15.87% (11.98-21.58)	16.20% (12.03-22.89)	17.38% (10.50-29.71)	15.83% (9.61-27.62)	13.20% (8.57-21.08)	11.20% (7.33-17.12)
			Both	0.09% (0.07-0.11)	0.57% (0.48-0.69)	1.72% (1.22-2.45)	2.61% (1.86-3.63)	3.50% (2.70-4.56)	4.80% (3.67-6.24)	7.08% (5.55-9.08)	9.23% (7.42-11.50)	11.13% (9.04-13.45)	12.69% (9.98-15.59)	13.66% (10.15-18.01)	14.45% (10.69-19.45)	15.19% (11.29-20.83)	15.50% (9.57-26.00)	14.30% (9.04-24.03)	12.48% (8.18-19.77)	11.02% (7.25-16.82)
		2019	Male	0.14% (0.10-0.18)	0.43% (0.34-0.55)	1.86% (1.20-2.80)	2.80% (1.94-4.04)	4.00% (2.92-5.34)	5.83% (4.25-7.79)	9.10% (6.88-12.08)	13.16% (9.96-18.45)	19.10% (13.82-26.15)	22.31% (15.92-29.82)	22.86% (15.32-32.05)	22.20% (15.20-31.24)	21.31% (15.75-29.91)	21.07% (12.48-35.21)	20.45% (11.89-34.42)	18.74% (11.07-31.04)	17.10% (9.84-28.61)
			Female	0.21% (0.17-0.27)	1.43% (1.14-1.77)	3.14% (2.28-4.28)	4.23% (3.16-5.72)	5.29% (4.15-6.66)	7.03% (5.46-9.01)	11.27% (8.60-14.74)	15.40% (12.26-19.40)	21.01% (16.05-27.08)	25.50% (19.65-32.60)	26.45% (19.08-35.26)	26.05% (18.59-35.56)	25.21% (18.36-34.88)	23.24% (14.28-37.45)	22.44% (13.35-36.84)	20.73% (12.30-33.85)	18.05% (10.82-29.90)
			Both	0.17% (0.13-0.21)	0.75% (0.62-0.90)	2.30% (1.59-3.27)	3.31% (2.36-4.61)	4.49% (3.43-5.78)	6.28% (4.78-8.21)	9.93% (7.70-13.00)	14.01% (10.88-18.53)	19.83% (15.02-26.11)	23.56% (17.54-30.56)	24.37% (16.87-33.65)	23.88% (16.81-32.90)	23.16% (17.06-31.82)	22.16% (13.40-36.00)	21.47% (12.76-35.32)	19.70% (11.72-32.10)	17.59% (10.29-29.23)
	Disability-Adjusted Life-Years	1990	Male	0.07% (0.06-0.09)	0.35% (0.29-0.44)	1.23% (0.88-1.74)	1.90% (1.40-2.61)	2.72% (2.12-3.53)	3.85% (3.00-4.97)	5.74% (4.55-7.31)	7.68% (6.16-9.61)	9.42% (7.65-11.45)	10.72% (8.60-13.23)	11.70% (8.82-15.35)	12.71% (9.48-17.01)	13.50% (10.23-18.53)	12.76% (8.47-20.28)	11.77% (7.83-18.82)	10.85% (7.26-16.82)	9.98% (6.53-15.29)
			Female	0.11% (0.09-0.13)	0.66% (0.54-0.80)	1.28% (0.98-1.65)	1.83% (1.43-2.32)	2.47% (2.00-3.01)	3.49% (2.88-4.25)	5.65% (4.61-6.88)	8.13% (6.87-9.64)	10.55% (8.84-12.46)	12.80% (10.64-15.34)	13.87% (11.02-17.55)	14.37% (11.17-19.10)	14.86% (11.34-20.66)	15.92% (10.07-26.17)	14.47% (9.15-24.18)	12.06% (7.95-18.52)	10.17% (6.86-15.01)
			Both	0.09% (0.07-0.11)	0.49% (0.41-0.59)	1.25% (0.95-1.69)	1.86% (1.43-2.44)	2.60% (2.08-3.20)	3.68% (2.98-4.58)	5.70% (4.63-7.01)	7.88% (6.56-9.45)	9.89% (8.27-11.75)	11.58% (9.51-14.03)	12.63% (9.75-16.39)	13.46% (10.30-17.62)	14.17% (10.77-19.10)	14.42% (9.47-23.28)	13.26% (8.71-21.32)	11.53% (7.80-17.68)	10.10% (6.75-14.97)
		2019	Male	0.15% (0.12-0.18)	0.63% (0.49-0.80)	1.78% (1.34-2.39)	2.63% (2.00-3.40)	3.68% (2.95-4.49)	5.28% (4.17-6.56)	8.19% (6.67-10.14)	12.00% (9.65-15.81)	17.25% (13.27-22.73)	20.48% (15.55-26.50)	21.31% (15.37-28.32)	20.95% (15.14-28.15)	20.21% (15.47-27.16)	19.78% (12.61-31.53)	19.08% (11.84-30.56)	17.45% (10.87-27.53)	15.88% (9.53-25.72)
			Female	0.20% (0.16-0.24)	0.89% (0.71-1.10)	1.77% (1.36-2.34)	2.46% (1.96-3.10)	3.30% (2.68-3.99)	4.68% (3.80-5.69)	7.85% (6.34-9.80)	11.60% (9.64-14.04)	16.56% (13.26-20.54)	21.13% (17.15-25.95)	22.89% (17.65-29.43)	23.19% (17.57-30.42)	22.89% (17.45-30.59)	21.27% (13.92-32.21)	20.58% (13.04-32.52)	18.78% (11.90-29.75)	16.25% (10.15-25.72)
			Both	0.17% (0.14-0.21)	0.75% (0.61-0.92)	1.78% (1.36-2.32)	2.54% (1.99-3.23)	3.49% (2.85-4.19)	5.00% (4.04-6.07)	8.03% (6.58-9.80)	11.82% (9.72-14.75)	16.95% (13.47-21.41)	20.76% (16.35-26.21)	22.02% (16.43-28.80)	21.97% (16.50-29.15)	21.51% (16.54-28.52)	20.53% (13.35-31.96)	19.85% (12.52-31.34)	18.10% (11.44-28.64)	16.07% (10.00-25.48)

Supplementary Table 3

Risk	Measure	Year	Sex	Under 20	20 to 24	25 to 29	30 to 34	35 to 39	40 to 44	45 to 49	50 to 54	55 to 59	60 to 64	65 to 69	70 to 74	75 to 79	80 to 84	85 to 89	90 to 94	95 plus
High Body-Mass Index	Deaths	1990	Male	0.01% (0.00-0.02)	2.07% (0.72-3.88)	4.57% (2.06-7.68)	7.39% (3.49-11.82)	10.17% (5.90-14.67)	12.44% (7.29-18.26)	15.78% (9.33-22.59)	17.04% (10.10-24.17)	16.74% (9.90-24.24)	15.93% (9.15-23.69)	14.15% (7.85-21.27)	11.29% (5.88-17.91)	9.57% (4.61-15.89)	5.58% (2.21-10.55)	5.93% (2.41-10.97)	6.38% (2.61-11.76)	6.80% (2.79-12.53)
			Female	0.02% (0.01-0.03)	4.52% (1.94-7.35)	7.09% (3.96-10.38)	10.50% (6.26-14.56)	13.55% (9.54-17.62)	16.79% (11.75-21.83)	21.98% (15.73-28.04)	25.85% (18.03-33.46)	26.31% (18.30-34.12)	26.66% (18.11-35.50)	24.50% (16.20-33.35)	20.26% (12.80-28.35)	16.97% (10.42-24.57)	9.75% (4.73-15.83)	10.14% (5.07-16.37)	10.65% (5.31-17.29)	11.03% (5.49-17.81)
			Both	0.01% (0.01-0.02)	2.96% (1.21-5.06)	5.58% (2.87-8.68)	8.66% (4.71-12.80)	11.57% (7.52-15.60)	14.15% (9.14-19.39)	18.22% (11.92-24.77)	20.46% (13.37-27.65)	20.32% (13.08-27.86)	20.06% (12.84-28.01)	18.34% (11.52-26.04)	15.21% (9.00-22.46)	13.11% (7.50-19.92)	7.76% (3.63-13.27)	8.23% (3.96-13.89)	8.76% (4.26-14.70)	9.39% (4.59-15.74)
		2019	Male	0.01% (0.01-0.02)	4.80% (2.71-6.96)	7.02% (4.01-9.91)	9.87% (5.71-13.93)	13.79% (9.67-18.21)	17.95% (12.41-23.83)	22.98% (15.88-30.18)	25.70% (17.65-33.29)	25.94% (17.66-33.94)	24.96% (16.76-33.13)	22.67% (14.74-30.76)	18.54% (11.15-26.18)	15.52% (8.94-22.71)	9.71% (5.08-15.61)	10.08% (5.25-16.26)	10.63% (5.50-17.31)	11.15% (5.83-18.03)
			Female	0.02% (0.01-0.03)	7.61% (4.68-10.26)	9.47% (6.00-12.57)	12.73% (8.11-16.46)	15.85% (12.08-19.59)	19.30% (14.80-23.86)	24.21% (18.51-29.60)	30.33% (22.77-37.03)	31.90% (23.85-39.07)	32.82% (24.32-41.02)	31.08% (22.15-39.19)	26.63% (18.22-34.75)	22.83% (15.48-30.65)	14.49% (8.47-21.32)	14.80% (8.70-21.83)	15.38% (8.97-22.55)	15.90% (9.14-23.64)
			Both	0.01% (0.01-0.02)	5.69% (3.48-7.95)	7.86% (4.75-10.56)	10.88% (6.60-14.61)	14.56% (10.71-18.45)	18.45% (13.47-23.63)	23.45% (17.06-29.53)	27.46% (19.91-34.55)	28.22% (20.05-35.75)	28.05% (19.81-36.12)	26.20% (18.12-34.23)	22.08% (14.47-29.80)	19.00% (12.30-26.50)	12.11% (6.78-18.46)	12.50% (7.04-19.00)	12.93% (7.14-19.76)	13.59% (7.52-20.66)
	Disability-Adjusted Life-Years	1990	Male	0.02% (0.01-0.03)	1.60% (0.57-2.96)	3.30% (1.55-5.42)	5.23% (2.68-8.16)	7.40% (4.40-10.64)	9.44% (5.63-13.70)	12.58% (7.44-18.00)	14.39% (8.62-20.54)	14.65% (8.68-21.17)	14.30% (8.30-21.13)	12.86% (7.24-19.22)	10.40% (5.44-16.36)	8.87% (4.30-14.64)	5.29% (2.13-9.79)	5.61% (2.35-10.17)	6.02% (2.51-11.03)	6.42% (2.66-11.64)
			Female	0.03% (0.01-0.04)	2.41% (1.08-3.96)	3.80% (2.19-5.49)	5.55% (3.45-7.72)	7.76% (5.51-10.27)	10.02% (7.05-13.22)	14.37% (10.22-18.74)	18.59% (12.93-23.97)	20.31% (13.98-26.53)	21.93% (14.86-29.02)	20.85% (14.14-28.48)	17.80% (11.34-24.94)	15.26% (9.49-21.95)	9.02% (4.51-14.55)	9.38% (4.79-15.14)	9.83% (5.01-15.76)	10.19% (5.17-16.47)
			Both	0.02% (0.01-0.04)	1.97% (0.82-3.34)	3.55% (1.87-5.34)	5.39% (3.10-7.91)	7.58% (4.95-10.44)	9.71% (6.36-13.35)	13.40% (8.77-18.30)	16.24% (10.65-21.78)	17.01% (10.98-23.22)	17.47% (11.32-24.30)	16.28% (10.20-23.12)	13.74% (8.16-20.17)	11.99% (6.88-18.09)	7.26% (3.47-12.29)	7.69% (3.76-12.82)	8.17% (4.06-13.67)	8.74% (4.38-14.50)
		2019	Male	0.07% (0.03-0.12)	3.41% (2.07-4.88)	4.93% (3.11-6.66)	6.80% (4.45-9.18)	9.60% (6.77-12.51)	12.93% (9.15-17.02)	17.48% (12.20-22.67)	21.00% (14.60-27.06)	22.34% (15.53-28.92)	22.33% (15.30-29.30)	20.73% (13.75-27.89)	17.26% (10.50-24.05)	14.54% (8.58-21.12)	9.27% (5.03-14.72)	9.62% (5.09-15.25)	10.13% (5.42-16.34)	10.62% (5.49-16.98)
			Female	0.07% (0.03-0.12)	3.38% (2.19-4.73)	4.32% (2.99-5.83)	5.78% (4.05-7.57)	8.02% (6.06-10.11)	10.64% (8.15-13.34)	15.03% (11.34-18.52)	20.70% (15.58-25.65)	23.92% (17.94-29.56)	26.47% (19.90-33.24)	26.32% (19.11-33.35)	23.44% (16.15-30.52)	20.62% (13.93-27.38)	13.47% (8.10-19.60)	13.88% (8.26-20.26)	14.33% (8.51-21.02)	14.72% (8.54-21.76)
			Both	0.07% (0.03-0.12)	3.39% (2.13-4.73)	4.63% (3.09-6.19)	6.30% (4.23-8.28)	8.83% (6.55-11.26)	11.84% (8.74-14.98)	16.35% (11.89-20.70)	20.86% (15.14-26.51)	23.02% (16.63-29.10)	24.11% (17.37-30.85)	23.22% (16.17-30.21)	20.08% (13.17-26.81)	17.50% (11.40-24.19)	11.40% (6.57-17.16)	11.81% (6.74-17.68)	12.19% (6.92-18.33)	12.75% (7.28-19.23)
High LDL Cholesterol	Deaths	1990	Male	0.00% (0.00-0.00)	0.00% (0.00-0.00)	4.97% (3.89-6.30)	9.07% (7.60-11.00)	14.09% (12.11-16.48)	18.76% (16.01-21.68)	23.11% (19.83-26.81)	24.09% (20.80-28.05)	22.82% (19.38-26.69)	19.11% (15.28-23.09)	13.85% (8.82-18.60)	10.72% (5.86-15.86)	11.60% (7.48-16.22)	13.58% (7.11-20.79)	13.66% (7.16-20.86)	13.93% (7.16-21.59)	14.54% (7.38-22.91)
			Female	0.00% (0.00-0.00)	0.00% (0.00-0.00)	5.59% (4.46-6.83)	8.60% (7.21-9.98)	10.70% (9.22-12.57)	13.92% (11.91-16.38)	18.64% (16.13-21.50)	20.12% (17.48-23.41)	19.58% (16.63-22.84)	19.23% (15.69-23.18)	14.53% (9.62-19.48)	11.91% (6.47-17.25)	13.07% (8.44-18.22)	15.41% (8.57-23.27)	15.56% (8.46-23.69)	15.41% (8.37-23.86)	15.24% (7.95-24.24)
			Both	0.00% (0.00-0.00)	0.00% (0.00-0.00)	5.22% (4.21-6.35)	8.88% (7.62-10.40)	12.69% (11.07-14.42)	16.87% (14.59-19.39)	21.35% (18.48-24.50)	22.55% (19.61-25.88)	21.61% (18.54-24.98)	19.16% (15.48-23.02)	14.12% (9.14-19.01)	11.24% (6.12-16.40)	12.30% (8.01-17.16)	14.54% (7.83-22.07)	14.70% (7.97-22.40)	14.76% (7.84-22.86)	14.97% (7.68-23.70)
		2019	Male	0.00% (0.00-0.00)	0.00% (0.00-0.00)	5.13% (3.88-6.71)	8.59% (7.07-10.67)	12.88% (10.73-16.04)	17.88% (15.18-21.63)	22.39% (19.28-26.56)	23.46% (19.92-27.50)	21.83% (18.44-25.70)	18.55% (14.82-22.44)	13.40% (8.68-18.09)	10.29% (5.54-15.26)	11.49% (7.44-15.84)	13.57% (7.40-20.54)	13.66% (7.28-20.97)	13.59% (7.07-21.09)	13.74% (6.96-21.46)
			Female	0.00% (0.00-0.00)	0.00% (0.00-0.00)	6.22% (4.85-7.61)	9.55% (7.94-11.32)	11.24% (9.30-13.08)	14.07% (11.85-16.24)	17.86% (15.34-20.53)	19.18% (16.58-22.05)	19.06% (16.31-22.11)	18.69% (15.31-22.25)	14.14% (9.45-18.65)	11.41% (6.23-16.78)	12.98% (8.36-17.79)	15.48% (8.36-23.30)	15.88% (8.60-24.23)	15.47% (8.29-23.51)	15.18% (7.72-24.07)
			Both	0.00% (0.00-0.00)	0.00% (0.00-0.00)	5.51% (4.38-6.88)	8.93% (7.49-10.71)	12.27% (10.44-14.54)	16.45% (14.11-19.07)	20.66% (17.93-23.85)	21.84% (18.76-25.19)	20.77% (17.76-24.19)	18.60% (15.04-22.27)	13.71% (9.12-18.18)	10.78% (5.83-15.87)	12.20% (7.89-16.83)	14.53% (7.77-21.92)	14.80% (7.88-22.59)	14.50% (7.54-22.34)	14.48% (7.30-22.63)
	Disability-Adjusted Life-Years	1990	Male	0.00% (0.00-0.00)	0.00% (0.00-0.00)	3.11% (2.42-3.96)	5.51% (4.44-6.88)	8.85% (7.29-10.65)	12.42% (10.39-14.87)	16.54% (13.85-19.38)	18.46% (15.61-21.42)	18.26% (15.47-21.40)	15.76% (12.71-19.14)	11.54% (7.43-15.62)	9.04% (4.93-13.32)	9.88% (6.43-13.87)	11.63% (6.13-17.82)	11.62% (6.12-17.89)	11.82% (6.01-18.29)	12.33% (6.28-19.21)
			Female	0.00% (0.00-0.00)	0.00% (0.00-0.00)	2.53% (1.92-3.26)	3.77% (2.99-4.71)	5.03% (4.05-6.24)	6.84% (5.49-8.36)	10.44% (8.55-12.62)	12.65% (10.56-15.09)	13.33% (11.14-15.72)	14.17% (11.45-17.14)	11.12% (7.22-15.09)	9.46% (5.14-13.78)	10.70% (6.89-15.08)	12.86% (7.17-19.79)	12.94% (7.09-19.73)	12.77% (6.99-19.81)	12.65% (6.59-20.12)
			Both	0.00% (0.00-0.00)	0.00% (0.00-0.00)	2.82% (2.21-3.55)	4.65% (3.76-5.77)	6.97% (5.80-8.41)	9.79% (8.11-11.75)	13.74% (11.46-16.17)	15.90% (13.50-18.50)	16.20% (13.69-18.85)	15.10% (12.17-18.22)	11.36% (7.35-15.36)	9.23% (5.03-13.55)	10.28% (6.67-14.33)	12.28% (6.64-18.83)	12.35% (6.68-19.02)	12.36% (6.57-19.14)	12.53% (6.43-19.71)
		2019	Male	0.00% (0.00-0.00)	0.00% (0.00-0.00)	2.78% (2.07-3.73)	4.43% (3.39-5.75)	6.72% (5.32-8.50)	10.03% (8.10-12.59)	13.89% (11.40-17.11)	15.99% (13.30-19.22)	15.80% (13.03-18.79)	14.08% (11.21-17.32)	10.40% (6.79-14.14)	8.16% (4.32-12.10)	9.33% (6.11-13.03)	11.06% (5.98-16.75)	11.26% (5.98-17.14)	11.27% (5.84-17.41)	11.44% (5.71-17.88)
			Female	0.00% (0.00-0.00)	0.00% (0.00-0.00)	2.02% (1.43-2.71)	3.01% (2.23-4.02)	3.83% (2.86-5.02)	5.46% (4.15-6.96)	8.26% (6.42-10.24)	10.25% (8.25-12.47)	11.39% (9.36-13.79)	12.32% (9.94-15.02)	9.93% (6.73-13.37)	8.41% (4.61-12.41)	10.08% (6.53-14.10)	12.37% (6.74-18.61)	13.03% (7.20-19.64)	12.55% (6.77-19.18)	12.25% (6.22-19.44)
			Both	0.00% (0.00-0.00)	0.00% (0.00-0.00)	2.41% (1.80-3.25)	3.74% (2.85-4.84)	5.31% (4.14-6.72)	7.86% (6.24-9.69)	11.29% (9.08-13.83)	13.44% (11.13-16.11)	13.88% (11.53-16.63)	13.32% (10.77-16.30)	10.19% (6.78-13.75)	8.27% (4.41-12.20)	9.69% (6.26-13.49)	11.72% (6.36-17.67)	12.17% (6.63-18.38)	11.90% (6.30-18.33)	11.86% (5.94-18.59)

Supplementary Table 4

			The NAME	Afghanistan	Algeria	Bahrain	Egypt	Iran	Iraq	Jordan	Kuwait	Lebanon	Libya
High Systolic Blood Pressure	Deaths	Cardiovascular Diseases	55.55% (48.94-62.02)	53.45% (45.41-60.42)	54.00% (45.60-62.65)	51.49% (42.43-60.30)	55.14% (46.42-62.73)	52.25% (45.93-59.20)	61.19% (54.22-67.90)	57.91% (50.64-64.61)	56.48% (49.63-63.47)	54.59% (46.12-62.67)	59.49% (52.18-66.46)
		Diabetes and Kidney Diseases	35.17% (31.33-38.88)	32.50% (25.51-41.06)	41.45% (35.73-47.50)	14.60% (12.48-16.98)	36.25% (27.92-42.74)	29.59% (26.25-33.26)	33.40% (28.84-39.01)	31.33% (27.31-35.08)	31.52% (27.18-35.53)	38.43% (30.49-46.65)	41.72% (34.04-48.97)
	DALYs	Cardiovascular Diseases	56.09% (51.01-60.89)	53.93% (46.66-60.35)	53.70% (46.62-60.30)	53.40% (46.38-60.25)	55.43% (48.33-61.63)	53.37% (48.36-58.33)	63.40% (57.70-68.72)	59.08% (52.96-64.94)	56.17% (50.34-62.11)	57.73% (51.05-63.83)	59.34% (53.52-64.78)
		Diabetes and Kidney Diseases	22.74% (19.01-26.17)	21.85% (17.55-27.75)	22.84% (18.40-27.92)	10.83% (9.01-12.86)	25.81% (19.42-31.46)	17.60% (14.92-20.34)	23.35% (19.68-27.39)	22.40% (18.85-25.82)	14.90% (11.67-18.36)	20.82% (15.68-25.82)	23.66% (18.05-29.35)
High Fasting Plasma Glucose	Deaths	Cardiovascular Diseases	27.36% (17.78-41.33)	29.57% (20.65-42.53)	29.93% (18.02-46.84)	30.07% (19.36-45.44)	26.37% (16.02-40.86)	26.22% (16.41-40.66)	30.40% (20.77-43.89)	25.49% (17.14-37.18)	27.94% (19.07-39.90)	31.61% (18.83-48.82)	31.12% (20.32-46.92)
		Diabetes and Kidney Diseases	64.89% (61.17-68.53)	65.39% (57.27-72.13)	58.27% (52.59-63.93)	86.35% (84.20-88.26)	64.55% (58.62-73.16)	68.76% (65.68-71.58)	70.26% (65.32-74.27)	70.40% (66.64-74.02)	69.70% (65.62-73.60)	60.67% (52.73-68.31)	60.33% (53.31-67.35)
		Neoplasms	5.08% (1.45-10.08)	3.14% (0.91-6.45)	5.52% (1.60-10.87)	12.46% (4.13-22.44)	3.94% (1.12-8.00)	4.23% (1.21-8.38)	6.69% (1.98-13.09)	6.70% (1.96-12.99)	8.71% (2.64-16.56)	7.54% (2.23-14.68)	7.57% (2.24-14.84)
		Neurological Disorders	8.87% (1.88-19.87)	9.97% (2.06-22.45)	10.72% (2.35-23.78)	16.21% (3.93-34.22)	7.28% (1.42-17.19)	8.91% (1.91-20.06)	10.58% (2.31-23.86)	10.27% (2.27-22.98)	14.29% (3.40-30.02)	11.04% (2.47-24.52)	12.28% (2.82-26.90)
		Respiratory Infections and Tuberculosis	1.64% (0.97-2.41)	4.17% (2.24-6.50)	0.98% (0.52-1.59)	1.88% (0.99-2.93)	0.43% (0.25-0.72)	0.91% (0.48-1.42)	3.92% (2.27-5.89)	0.46% (0.24-0.72)	0.66% (0.33-1.04)	0.67% (0.34-1.24)	1.49% (0.80-2.37)
	DALYs	Cardiovascular Diseases	25.18% (17.86-34.87)	28.16% (20.22-39.97)	27.61% (19.10-39.46)	29.59% (20.75-41.74)	23.97% (15.51-35.50)	23.76% (16.32-34.67)	29.01% (21.15-39.84)	24.74% (17.86-34.54)	26.84% (19.42-35.97)	29.08% (19.18-42.56)	28.50% (20.00-40.18)
		Diabetes and Kidney Diseases	72.84% (69.60-76.43)	71.82% (65.40-76.67)	71.52% (66.63-76.08)	88.25% (86.54-89.87)	71.42% (66.35-77.72)	76.80% (74.12-79.68)	75.78% (72.30-79.18)	75.19% (72.11-78.22)	82.02% (78.67-85.25)	75.21% (70.08-80.24)	74.04% (68.88-79.22)
		Neoplasms	4.26% (1.20-8.52)	2.59% (0.74-5.38)	4.50% (1.30-8.96)	10.64% (3.47-19.34)	3.39% (0.96-6.93)	3.54% (1.00-7.13)	5.56% (1.63-11.00)	5.64% (1.62-11.08)	7.38% (2.19-14.21)	6.31% (1.84-12.43)	6.35% (1.84-12.59)
		Neurological Disorders	3.54% (0.63-9.73)	4.15% (0.73-11.37)	4.33% (0.75-11.91)	6.88% (1.42-17.84)	2.95% (0.48-8.44)	3.42% (0.60-9.37)	4.29% (0.80-12.19)	4.11% (0.73-11.47)	5.46% (1.06-14.45)	4.31% (0.81-11.85)	4.96% (0.90-13.39)
		Respiratory Infections and Tuberculosis	1.18% (0.74-1.63)	2.73% (1.44-4.24)	0.84% (0.51-1.23)	1.87% (1.10-2.69)	0.39% (0.24-0.62)	0.74% (0.45-1.05)	2.72% (1.65-3.94)	0.33% (0.18-0.48)	0.69% (0.42-1.00)	0.75% (0.42-1.25)	1.34% (0.78-1.95)
		Sense Organ Diseases	2.05% (0.54-4.23)	3.71% (0.96-7.68)	2.53% (0.68-5.24)	3.85% (1.14-7.38)	1.89% (0.48-4.06)	1.80% (0.50-3.73)	2.63% (0.70-5.39)	1.39% (0.38-2.89)	2.79% (0.76-5.64)	2.43% (0.63-5.03)	3.55% (1.00-7.27)
High Body-Mass Index	Deaths	Cardiovascular Diseases	25.88% (17.44-34.99)	21.50% (13.83-29.97)	23.00% (14.95-32.40)	25.21% (16.52-34.16)	30.28% (20.19-40.28)	22.32% (14.94-30.73)	26.33% (17.11-35.75)	32.73% (22.12-42.69)	33.66% (22.93-43.60)	26.87% (17.37-36.93)	30.32% (20.59-40.08)
		Chronic Respiratory Diseases	7.32% (4.61-10.51)	8.72% (4.35-15.01)	9.06% (5.47-13.22)	5.04% (3.10-7.24)	11.58% (6.66-17.54)	6.12% (3.82-8.72)	11.47% (6.85-16.49)	5.96% (3.78-8.26)	4.89% (3.19-6.78)	8.12% (4.53-12.33)	10.63% (6.51-15.46)
		Diabetes and Kidney Diseases	46.25% (33.46-59.00)	38.23% (26.55-50.29)	41.61% (27.94-56.84)	51.53% (36.70-66.02)	50.58% (36.97-63.56)	43.02% (31.46-54.90)	49.63% (34.76-63.69)	52.41% (38.00-65.93)	51.43% (36.91-66.00)	45.23% (31.47-59.65)	49.60% (35.68-63.35)
		Digestive Diseases	2.28% (1.53-3.18)	1.87% (0.92-3.05)	2.66% (1.63-3.89)	3.22% (2.03-5.17)	1.27% (0.71-2.63)	3.23% (1.82-4.43)	1.81% (1.17-2.65)	4.71% (3.10-6.45)	3.20% (2.04-4.51)	2.83% (1.26-4.61)	2.58% (1.60-3.79)
		Neoplasms	6.24% (3.93-8.91)	4.08% (2.30-6.16)	6.08% (3.70-8.69)	7.28% (4.59-10.21)	10.22% (5.98-15.14)	5.17% (3.26-7.44)	5.65% (3.38-8.16)	8.19% (5.33-11.18)	9.18% (6.01-12.48)	5.79% (3.50-8.29)	7.03% (4.42-9.73)
		Neurological Disorders	12.95% (4.69-24.18)	7.54% (2.46-15.51)	12.91% (4.64-24.33)	15.24% (5.21-28.27)	12.86% (4.07-24.55)	12.12% (4.61-22.62)	14.10% (4.90-26.38)	16.75% (6.01-29.94)	18.78% (7.40-32.76)	14.65% (5.26-27.25)	15.20% (5.74-27.90)
	DALYs	Cardiovascular Diseases	32.13% (22.56-41.81)	26.58% (17.65-35.81)	30.56% (21.05-40.17)	32.26% (22.52-41.54)	36.29% (25.39-46.43)	27.88% (19.57-36.55)	33.11% (22.53-43.47)	40.16% (28.78-50.20)	42.08% (30.65-51.98)	33.41% (22.53-44.22)	36.48% (25.86-46.44)
		Chronic Respiratory Diseases	8.73% (5.72-12.15)	8.71% (4.72-14.26)	9.71% (6.28-13.48)	8.06% (5.37-10.92)	11.63% (7.20-16.58)	7.17% (4.67-9.95)	12.11% (7.68-16.90)	9.81% (6.49-13.47)	12.75% (8.70-17.58)	9.25% (5.75-13.05)	10.68% (7.01-14.81)
		Diabetes and Kidney Diseases	53.94% (41.96-64.94)	43.91% (32.37-55.02)	53.25% (40.75-64.50)	61.22% (46.56-72.73)	57.63% (44.11-68.83)	51.52% (40.50-61.84)	56.98% (43.17-68.96)	60.89% (47.41-71.47)	64.32% (51.88-74.22)	56.49% (42.60-67.98)	59.06% (46.58-70.12)
		Digestive Diseases	2.82% (1.89-3.97)	2.24% (1.27-3.53)	3.26% (2.07-4.76)	3.35% (2.15-4.90)	1.90% (1.24-2.79)	4.13% (2.64-6.03)	2.97% (1.86-4.33)	4.78% (3.24-6.53)	3.94% (2.62-5.44)	3.44% (2.10-5.12)	3.23% (2.08-4.64)
		Musculoskeletal Disorders	7.66% (4.83-10.82)	5.06% (2.81-7.83)	7.43% (4.56-10.61)	8.78% (5.65-12.10)	8.68% (5.50-12.10)	6.15% (3.83-8.89)	8.38% (5.05-12.02)	9.27% (6.04-12.78)	10.42% (6.93-14.07)	7.98% (4.85-11.60)	8.50% (5.34-11.85)
		Neoplasms	5.97% (3.78-8.42)	3.88% (2.19-5.83)	5.48% (3.39-7.83)	7.27% (4.59-10.07)	9.99% (5.87-14.76)	4.86% (3.05-6.97)	5.23% (3.18-7.50)	7.68% (5.03-10.38)	8.95% (5.92-12.01)	5.34% (3.20-7.74)	6.65% (4.20-9.19)
		Neurological Disorders	5.33% (1.60-11.92)	3.31% (0.90-7.85)	5.53% (1.62-12.59)	6.56% (1.99-14.63)	5.46% (1.60-12.37)	4.76% (1.42-10.68)	5.78% (1.68-13.12)	6.92% (2.08-15.20)	7.57% (2.35-16.37)	5.96% (1.70-13.58)	6.40% (1.92-14.29)
		Sense Organ Diseases	1.42% (0.73-2.30)	1.51% (0.70-2.62)	1.60% (0.79-2.62)	1.77% (0.93-2.80)	1.79% (0.92-2.88)	0.98% (0.50-1.63)	1.61% (0.80-2.62)	0.99% (0.52-1.56)	2.02% (1.10-3.09)	1.76% (0.90-2.87)	2.14% (1.14-3.42)
High LDL Cholesterol	Deaths	Cardiovascular Diseases	28.77% (21.87-36.33)	27.46% (21.80-34.38)	26.48% (18.46-35.15)	31.77% (23.17-41.20)	30.78% (23.46-39.10)	28.75% (21.31-36.84)	28.93% (21.87-36.76)	27.68% (20.72-36.16)	32.39% (25.57-39.93)	37.40% (28.80-46.56)	29.86% (22.64-37.92)
	DALYs	Cardiovascular Diseases	31.51% (26.05-37.29)	31.31% (26.05-37.57)	29.00% (22.72-35.96)	33.85% (27.15-40.89)	33.02% (27.15-39.62)	31.01% (25.38-37.25)	31.29% (25.69-37.41)	30.94% (25.08-37.26)	36.73% (31.48-42.64)	41.00% (34.73-47.68)	32.07% (26.27-38.53)

Supplementary Table 4

			Morocco	Oman	Palestine	Qatar	Saudi Arabia	Sudan	Syria	The UAE	Tunisia	Turkey	Yemen
High Systolic Blood Pressure	Deaths	Cardiovascular Diseases	61.47% (54.06-68.80)	53.65% (45.02-61.93)	49.99% (42.53-57.13)	51.71% (41.32-62.37)	52.47% (45.45-59.30)	63.74% (56.42-70.30)	50.36% (41.43-59.06)	56.43% (46.69-65.36)	50.44% (42.28-58.35)	54.29% (47.20-61.36)	51.76% (43.68-59.19)
		Diabetes and Kidney Diseases	42.18% (36.22-47.96)	16.70% (14.06-19.50)	20.03% (17.09-22.68)	19.12% (16.08-22.54)	48.19% (42.29-53.21)	44.67% (37.71-52.45)	39.59% (34.57-44.17)	29.62% (21.13-39.08)	38.39% (32.89-43.51)	32.84% (29.06-36.58)	37.53% (31.96-43.16)
	DALYs	Cardiovascular Diseases	62.65% (56.37-68.72)	55.75% (48.47-62.09)	51.35% (44.62-57.48)	53.64% (45.48-61.97)	53.91% (47.89-59.82)	64.14% (58.66-69.24)	51.39% (44.13-58.09)	57.78% (49.31-65.91)	52.43% (45.51-58.96)	54.77% (48.99-60.46)	52.26% (44.86-58.77)
		Diabetes and Kidney Diseases	27.32% (22.74-32.13)	13.53% (11.25-15.75)	15.64% (13.13-18.21)	13.47% (11.23-16.28)	30.64% (25.36-35.61)	26.92% (21.28-33.42)	23.31% (18.98-27.76)	21.23% (15.45-28.23)	20.25% (15.64-24.76)	21.64% (18.19-25.13)	23.96% (19.91-28.14)
High Fasting Plasma Glucose	Deaths	Cardiovascular Diseases	30.76% (19.85-46.09)	31.90% (19.60-48.64)	31.57% (20.83-46.34)	30.74% (18.32-47.26)	29.83% (21.33-41.16)	29.82% (19.75-44.65)	33.19% (20.37-50.75)	26.66% (17.42-38.93)	31.01% (19.51-47.39)	21.79% (13.52-34.27)	21.35% (13.48-33.42)
		Diabetes and Kidney Diseases	59.45% (53.43-65.11)	84.33% (81.75-86.94)	79.13% (76.43-81.68)	82.60% (79.27-85.18)	52.61% (47.31-57.80)	56.72% (48.82-63.64)	58.53% (53.97-63.50)	71.16% (62.12-78.77)	57.53% (52.43-63.37)	67.56% (63.97-70.92)	59.16% (53.49-64.51)
		Neoplasms	5.55% (1.54-11.31)	5.77% (1.70-11.25)	7.85% (2.28-15.20)	10.96% (3.63-19.84)	6.66% (1.99-12.95)	4.01% (1.14-8.10)	4.55% (1.31-9.08)	9.14% (2.82-18.00)	7.90% (2.20-15.56)	5.24% (1.38-10.73)	2.65% (0.73-5.52)
		Neurological Disorders	9.34% (1.96-21.14)	8.95% (1.90-21.40)	10.35% (2.22-23.81)	15.45% (3.38-33.93)	11.24% (2.46-25.67)	9.71% (2.02-22.12)	9.82% (2.10-21.92)	12.03% (2.50-27.95)	11.63% (2.65-25.54)	7.36% (1.43-17.74)	7.32% (1.54-16.60)
		Respiratory Infections and Tuberculosis	5.05% (2.82-8.61)	0.49% (0.23-0.84)	0.74% (0.38-1.18)	1.50% (0.77-2.30)	3.44% (1.89-5.26)	2.18% (1.13-3.62)	0.39% (0.21-0.60)	1.04% (0.42-1.83)	0.99% (0.53-1.61)	0.57% (0.31-0.89)	1.71% (0.92-2.75)
	DALYs	Cardiovascular Diseases	28.69% (20.09-41.65)	30.74% (20.70-43.74)	31.06% (21.91-43.96)	30.74% (20.46-43.99)	28.99% (21.37-39.19)	27.25% (19.31-38.95)	29.59% (19.77-42.19)	26.32% (18.35-36.19)	28.91% (20.11-40.99)	19.57% (13.32-28.26)	19.35% (12.59-28.86)
		Diabetes and Kidney Diseases	69.54% (65.35-73.91)	84.86% (82.79-86.99)	80.51% (78.17-82.77)	86.20% (83.71-88.12)	65.94% (61.35-70.74)	68.61% (62.12-73.95)	70.05% (65.88-74.48)	76.16% (69.74-81.37)	73.84% (68.90-78.81)	74.43% (71.20-77.59)	68.01% (63.60-72.34)
		Neoplasms	4.88% (1.33-10.12)	4.97% (1.44-9.83)	6.67% (1.92-13.03)	10.25% (3.39-18.55)	5.64% (1.66-11.04)	3.30% (0.94-6.81)	3.70% (1.04-7.44)	7.62% (2.35-15.07)	6.64% (1.79-13.27)	4.41% (1.15-9.20)	2.22% (0.61-4.69)
		Neurological Disorders	3.77% (0.68-10.70)	4.31% (0.78-11.63)	4.37% (0.80-12.10)	7.44% (1.50-19.18)	4.58% (0.86-12.57)	3.74% (0.62-10.57)	4.01% (0.70-11.27)	5.02% (0.97-13.43)	4.63% (0.82-12.64)	2.88% (0.52-8.15)	2.88% (0.48-8.29)
		Respiratory Infections and Tuberculosis	3.87% (2.31-6.45)	0.56% (0.30-0.88)	0.70% (0.40-1.03)	1.51% (0.89-2.12)	3.28% (2.03-4.61)	1.57% (0.80-2.52)	0.34% (0.21-0.50)	1.56% (0.73-2.44)	0.88% (0.52-1.34)	0.57% (0.34-0.83)	1.21% (0.70-1.91)
		Sense Organ Diseases	2.13% (0.55-4.50)	3.31% (0.83-6.81)	3.12% (0.80-6.39)	4.32% (1.33-8.27)	3.73% (1.00-7.57)	2.05% (0.56-4.23)	2.34% (0.61-4.83)	3.27% (0.93-6.50)	2.47% (0.66-5.09)	1.32% (0.33-2.79)	1.52% (0.39-3.24)
High Body-Mass Index	Deaths	Cardiovascular Diseases	23.47% (14.75-32.99)	25.53% (17.03-34.93)	20.42% (12.64-29.59)	28.14% (18.42-37.68)	31.90% (22.30-40.83)	22.71% (14.45-31.89)	22.81% (14.43-31.89)	35.89% (25.46-46.75)	23.28% (14.83-32.65)	26.80% (17.16-36.72)	13.89% (7.60-21.28)
		Chronic Respiratory Diseases	9.39% (5.52-13.99)	0.90% (0.55-1.34)	4.94% (2.62-7.96)	2.65% (1.74-3.62)	8.15% (5.13-11.55)	8.74% (4.61-14.08)	10.74% (6.21-15.85)	11.98% (6.72-20.61)	7.97% (4.66-11.81)	2.97% (1.74-4.56)	5.15% (2.44-8.86)
		Diabetes and Kidney Diseases	41.77% (27.93-55.66)	49.18% (35.64-62.57)	39.97% (27.08-54.02)	53.02% (37.37-68.44)	51.22% (36.62-65.60)	38.25% (25.13-51.45)	40.15% (27.00-53.65)	55.83% (42.87-68.55)	41.84% (27.59-55.87)	47.57% (33.03-61.66)	26.07% (15.28-37.61)
		Digestive Diseases	2.44% (1.42-3.63)	1.46% (0.81-2.18)	2.31% (1.11-3.92)	1.63% (0.94-2.75)	3.23% (2.15-4.56)	1.91% (0.95-3.31)	1.59% (0.84-2.37)	3.15% (1.29-5.08)	2.84% (1.69-4.18)	5.35% (3.48-7.35)	1.30% (0.58-2.26)
		Neoplasms	4.62% (2.71-6.82)	7.64% (4.77-10.76)	5.40% (3.19-8.05)	11.18% (7.34-15.10)	9.73% (6.40-13.07)	5.07% (2.90-7.64)	6.04% (3.55-8.90)	10.73% (6.73-15.02)	4.96% (2.99-7.23)	5.70% (3.48-8.01)	2.49% (1.24-4.08)
		Neurological Disorders	11.30% (3.77-21.69)	13.71% (4.73-26.22)	8.56% (2.38-18.12)	16.99% (4.99-32.31)	15.89% (5.45-29.16)	10.02% (3.44-19.84)	12.12% (3.95-22.87)	15.36% (4.75-28.59)	13.22% (4.77-24.98)	14.38% (4.93-27.63)	4.84% (1.37-11.10)
	DALYs	Cardiovascular Diseases	29.56% (19.21-39.83)	31.70% (21.39-41.21)	27.03% (17.72-37.09)	34.69% (24.37-44.03)	40.63% (29.57-50.36)	28.64% (19.08-38.87)	29.80% (19.82-39.91)	44.20% (33.36-54.19)	29.84% (19.63-40.12)	33.04% (22.19-43.22)	18.37% (10.59-26.58)
		Chronic Respiratory Diseases	9.53% (5.97-13.73)	6.70% (4.22-9.96)	6.65% (4.04-9.75)	8.49% (5.72-11.86)	9.36% (6.34-12.49)	9.40% (5.61-14.07)	10.19% (6.34-14.39)	14.68% (9.55-21.52)	9.07% (5.71-12.98)	6.29% (3.90-9.03)	5.69% (2.87-9.09)
		Diabetes and Kidney Diseases	49.97% (36.49-62.41)	58.29% (45.02-69.14)	49.56% (36.61-62.36)	64.47% (50.49-75.33)	59.89% (47.56-70.68)	46.35% (33.44-58.07)	49.78% (37.24-61.47)	63.67% (52.25-73.17)	52.94% (39.35-64.88)	55.60% (41.90-66.89)	33.09% (20.73-44.63)
		Digestive Diseases	3.10% (1.94-4.53)	2.22% (1.41-3.19)	2.99% (1.82-4.47)	1.98% (1.23-2.90)	3.63% (2.49-4.85)	2.39% (1.41-3.66)	2.44% (1.50-3.67)	3.53% (2.05-5.06)	2.72% (1.71-3.96)	4.94% (3.27-6.93)	1.74% (0.90-2.79)
		Musculoskeletal Disorders	6.85% (3.97-10.07)	8.86% (5.73-12.29)	7.19% (4.25-10.62)	11.30% (7.75-15.16)	9.85% (6.56-13.27)	6.34% (3.71-9.37)	7.84% (4.64-11.47)	10.64% (7.26-14.24)	7.10% (4.20-10.43)	8.37% (5.12-11.92)	3.47% (1.74-5.56)
		Neoplasms	3.99% (2.27-5.94)	7.71% (4.83-10.73)	5.37% (3.26-7.87)	11.02% (7.29-14.74)	9.29% (6.16-12.36)	4.75% (2.72-7.11)	5.72% (3.35-8.31)	10.90% (6.89-15.17)	4.51% (2.70-6.57)	5.40% (3.31-7.49)	2.44% (1.24-4.01)
		Neurological Disorders	4.82% (1.37-10.92)	6.62% (2.00-14.84)	3.83% (0.95-9.32)	8.33% (2.36-18.16)	6.72% (2.07-14.89)	4.07% (1.13-9.42)	5.26% (1.46-11.96)	6.56% (1.93-14.57)	5.52% (1.58-12.49)	5.77% (1.76-13.04)	2.12% (0.52-5.48)
		Sense Organ Diseases	1.40% (0.65-2.38)	2.69% (1.41-4.25)	1.54% (0.71-2.66)	2.08% (1.13-3.19)	2.84% (1.51-4.45)	0.96% (0.45-1.63)	1.54% (0.76-2.55)	2.03% (1.13-3.14)	1.43% (0.70-2.41)	1.30% (0.65-2.11)	0.57% (0.24-1.08)
High LDL Cholesterol	Deaths	Cardiovascular Diseases	29.42% (21.89-37.67)	33.69% (24.76-43.24)	28.27% (21.25-36.93)	30.23% (20.33-41.18)	30.99% (23.92-38.62)	25.22% (18.55-32.85)	34.08% (25.79-43.25)	31.60% (23.61-40.77)	29.62% (21.94-38.21)	24.50% (17.82-32.07)	27.77% (21.22-35.72)
	DALYs	Cardiovascular Diseases	32.33% (25.99-38.72)	35.30% (28.00-42.85)	31.68% (25.70-38.38)	31.35% (23.47-39.65)	34.84% (28.89-41.23)	27.96% (22.17-34.16)	37.01% (30.44-43.63)	34.54% (27.74-42.27)	32.28% (26.01-39.23)	26.60% (21.17-32.13)	31.01% (25.33-37.19)

Supplementary Table 5

		SEV 1990	SEV 2010	SEV 2019	ARC 1990-2019	ARC 1990-2010	ARC 2010-2019
High Systolic Blood Pressure	The NAME	26.16 (23.97-28.25)	28.21 (26.46-30.02)	28.89 (30.92-26.93)	0.34%	0.38%	0.27%
	Afghanistan	21.79 (16.90-26.87)	21.67 (17.00-27.12)	22.89 (28.21-18.05)	0.17%	-0.03%	0.61%
	Algeria	28.59 (23.54-34.69)	22.72 (19.45-26.21)	25.37 (30.61-20.59)	-0.41%	-1.14%	1.23%
	Bahrain	29.38 (23.98-35.83)	30.53 (24.78-36.62)	31.52 (37.69-26.20)	0.24%	0.19%	0.36%
	Egypt	20.29 (16.09-25.16)	29.94 (26.82-32.86)	30.15 (34.80-25.62)	1.37%	1.96%	0.08%
	Iran	21.51 (19.67-23.35)	22.93 (21.30-24.58)	25.20 (27.21-23.24)	0.55%	0.32%	1.05%
	Iraq	33.89 (27.57-40.65)	36.46 (32.87-40.15)	39.12 (43.16-35.34)	0.50%	0.37%	0.78%
	Jordan	25.09 (20.20-30.15)	24.79 (22.05-27.51)	27.64 (32.73-22.67)	0.34%	-0.06%	1.22%
	Kuwait	25.11 (20.76-30.14)	26.37 (24.34-28.25)	24.36 (27.78-21.19)	-0.10%	0.24%	-0.87%
	Lebanon	25.86 (20.59-31.55)	27.60 (25.26-29.88)	34.52 (39.52-29.54)	1.00%	0.33%	2.52%
	Libya	32.36 (27.74-37.54)	41.23 (37.86-44.66)	40.28 (46.72-34.10)	0.76%	1.22%	-0.26%
	Morocco	39.67 (33.80-46.13)	38.73 (33.08-44.93)	39.19 (46.33-32.56)	-0.04%	-0.12%	0.13%
	Oman	17.22 (13.57-21.24)	32.59 (28.94-36.47)	34.56 (40.69-28.74)	2.43%	3.24%	0.65%
	Palestine	21.13 (17.43-24.99)	18.94 (16.92-20.93)	21.50 (25.53-17.79)	0.06%	-0.54%	1.41%
	Qatar	27.02 (22.06-32.22)	28.00 (25.47-30.64)	29.06 (33.79-24.73)	0.25%	0.18%	0.42%
	Saudi Arabia	24.15 (19.67-29.16)	25.10 (22.43-27.74)	25.36 (29.29-21.84)	0.17%	0.19%	0.12%
	Sudan	33.45 (27.51-40.20)	37.91 (35.49-40.54)	39.84 (44.87-34.90)	0.60%	0.63%	0.55%
	Syria	25.66 (20.58-30.99)	27.45 (22.41-33.53)	27.74 (34.07-22.38)	0.27%	0.34%	0.12%
	The UAE	36.77 (33.78-40.00)	24.42 (21.69-27.20)	27.25 (32.35-22.85)	-1.03%	-2.03%	1.23%
	Tunisia	24.19 (19.07-29.65)	24.84 (20.82-29.29)	27.23 (32.80-21.87)	0.41%	0.13%	1.03%
	Turkey	27.12 (22.51-31.58)	28.35 (26.00-30.93)	26.12 (30.04-22.52)	-0.13%	0.22%	-0.91%
	Yemen	20.16 (15.98-25.29)	21.20 (18.51-24.07)	22.90 (28.27-18.29)	0.44%	0.25%	0.86%
High Fasting Plasma Glucose	The NAME	7.91 (6.97-8.92)	12.94 (11.71-14.17)	15.22 (16.79-13.66)	2.28%	2.49%	1.82%
	Afghanistan	9.58 (8.19-11.14)	16.75 (14.76-18.89)	19.45 (21.82-17.19)	2.47%	2.83%	1.68%
	Algeria	8.86 (7.70-10.15)	14.40 (12.56-16.26)	17.26 (19.47-15.27)	2.33%	2.46%	2.03%
	Bahrain	16.81 (14.99-18.52)	25.70 (23.78-27.68)	27.70 (29.95-25.66)	1.74%	2.14%	0.84%
	Egypt	5.15 (4.56-5.79)	9.79 (8.46-11.17)	13.34 (15.68-11.32)	3.34%	3.27%	3.50%
	Iran	7.43 (6.47-8.49)	12.10 (10.76-13.50)	14.55 (16.18-12.93)	2.34%	2.47%	2.07%
	Iraq	11.74 (10.22-13.40)	16.57 (14.81-18.50)	18.31 (20.16-16.48)	1.55%	1.74%	1.12%
	Jordan	11.65 (10.27-13.12)	15.96 (14.36-17.68)	16.53 (18.21-14.82)	1.21%	1.58%	0.39%
	Kuwait	15.17 (13.44-17.14)	19.92 (18.36-21.26)	21.31 (23.45-19.24)	1.18%	1.37%	0.75%
	Lebanon	9.62 (8.25-11.17)	14.98 (13.14-16.87)	17.42 (19.61-15.39)	2.07%	2.24%	1.69%
	Libya	11.09 (9.48-12.82)	19.08 (17.02-21.29)	21.14 (23.73-18.80)	2.25%	2.75%	1.15%
	Morocco	7.27 (6.30-8.38)	13.13 (11.35-15.17)	16.10 (18.26-14.02)	2.78%	3.00%	2.29%
	Oman	9.33 (8.15-10.73)	13.16 (11.81-14.48)	17.00 (18.87-15.14)	2.09%	1.73%	2.89%
	Palestine	10.37 (8.89-12.02)	15.83 (14.29-17.54)	18.75 (20.57-16.87)	2.07%	2.14%	1.90%
	Qatar	18.51 (16.62-20.43)	30.95 (28.13-33.77)	31.27 (33.72-28.89)	1.82%	2.60%	0.12%
	Saudi Arabia	11.93 (10.50-13.52)	17.29 (15.67-19.05)	20.01 (22.25-17.94)	1.80%	1.87%	1.64%
	Sudan	7.67 (6.57-8.89)	13.85 (11.97-15.83)	16.65 (18.72-14.53)	2.71%	3.00%	2.07%
	Syria	9.21 (8.01-10.59)	13.64 (11.92-15.60)	15.90 (17.86-13.88)	1.90%	1.98%	1.72%
	The UAE	15.83 (14.05-17.59)	22.19 (20.42-24.18)	22.76 (25.14-20.72)	1.26%	1.70%	0.29%
	Tunisia	9.53 (8.29-10.85)	15.45 (13.91-16.96)	17.94 (20.12-15.84)	2.21%	2.45%	1.67%
	Turkey	7.67 (6.81-8.62)	11.34 (10.19-12.62)	11.14 (12.60-9.81)	1.29%	1.97%	-0.20%
	Yemen	5.78 (5.03-6.57)	8.87 (7.72-10.08)	10.73 (12.85-8.88)	2.15%	2.16%	2.14%

Supplementary Table 5

		SEV 1990	SEV 2010	SEV 2019	ARC 1990-2019	ARC 1990-2010	ARC 2010-2019
High Body-Mass Index	The NAME	18.93 (14.29-24.89)	28.22 (22.27-35.57)	33.31 (41.45-26.43)	1.97%	2.02%	1.86%
	Afghanistan	8.05 (5.12-11.75)	8.76 (5.82-12.41)	12.76 (16.82-9.42)	1.60%	0.42%	4.27%
	Algeria	17.38 (12.79-23.50)	27.55 (21.51-35.24)	32.68 (41.12-25.58)	2.20%	2.33%	1.91%
	Bahrain	30.62 (23.00-40.19)	37.83 (28.40-47.58)	42.47 (51.77-32.62)	1.13%	1.06%	1.29%
	Egypt	23.83 (17.52-31.84)	36.12 (27.41-45.47)	42.12 (51.63-32.32)	1.98%	2.10%	1.72%
	Iran	15.20 (11.65-19.19)	25.88 (21.40-31.68)	30.57 (37.58-25.61)	2.44%	2.70%	1.87%
	Iraq	26.59 (17.19-35.85)	27.74 (19.40-36.39)	32.50 (42.38-24.13)	0.69%	0.21%	1.77%
	Jordan	25.34 (18.86-33.16)	37.89 (28.74-46.91)	42.74 (52.37-32.73)	1.82%	2.03%	1.35%
	Kuwait	37.23 (28.49-46.53)	49.23 (38.65-58.81)	53.49 (62.54-41.54)	1.26%	1.41%	0.93%
	Lebanon	21.51 (15.76-28.95)	29.94 (22.82-38.90)	35.33 (44.72-27.24)	1.73%	1.67%	1.86%
	Libya	26.61 (19.86-35.38)	39.40 (30.57-48.78)	39.04 (48.46-29.96)	1.33%	1.98%	-0.10%
	Morocco	16.15 (11.91-21.82)	24.20 (18.69-31.60)	29.55 (37.97-22.91)	2.11%	2.04%	2.24%
	Oman	13.63 (10.10-18.00)	36.26 (28.86-45.04)	42.32 (51.90-33.45)	3.98%	5.01%	1.73%
	Palestine	16.13 (10.73-23.35)	22.42 (16.47-30.34)	26.54 (35.16-20.04)	1.73%	1.66%	1.90%
	Qatar	34.89 (25.83-44.88)	49.52 (37.84-59.12)	56.52 (66.28-43.40)	1.68%	1.77%	1.48%
	Saudi Arabia	22.40 (17.16-29.31)	42.04 (32.65-51.29)	49.96 (59.14-38.97)	2.80%	3.20%	1.94%
	Sudan	9.06 (6.04-12.59)	17.51 (13.37-22.24)	24.65 (31.40-19.75)	3.51%	3.35%	3.87%
	Syria	19.76 (14.15-27.03)	29.28 (22.32-38.03)	30.66 (39.51-23.50)	1.53%	1.98%	0.51%
	The UAE	33.53 (25.86-42.46)	50.49 (39.56-59.07)	53.64 (61.83-41.79)	1.63%	2.07%	0.68%
	Tunisia	18.82 (14.18-25.22)	28.50 (22.12-36.89)	33.15 (42.45-25.97)	1.97%	2.09%	1.69%
	Turkey	21.84 (15.99-29.58)	30.38 (23.01-39.62)	36.70 (46.83-27.93)	1.81%	1.66%	2.12%
	Yemen	6.21 (3.81-9.32)	9.73 (6.76-12.95)	10.95 (14.13-7.78)	1.98%	2.27%	1.33%
High LDL Cholesterol	The NAME	33.13 (30.08-36.36)	35.13 (32.14-38.42)	34.96 (38.17-31.97)	0.19%	0.29%	-0.05%
	Afghanistan	31.02 (27.77-34.38)	31.90 (28.64-35.36)	32.90 (36.47-29.63)	0.20%	0.14%	0.34%
	Algeria	28.57 (25.29-32.15)	30.11 (26.78-33.59)	31.15 (34.51-27.78)	0.30%	0.26%	0.38%
	Bahrain	47.05 (43.87-50.20)	43.62 (40.54-46.93)	44.13 (47.26-40.73)	-0.22%	-0.38%	0.13%
	Egypt	30.05 (26.86-33.52)	31.90 (28.56-35.38)	33.59 (37.04-30.39)	0.39%	0.30%	0.58%
	Iran	39.23 (36.28-42.31)	37.84 (34.93-40.99)	37.74 (40.89-34.80)	-0.13%	-0.18%	-0.03%
	Iraq	31.98 (28.71-35.40)	34.57 (31.37-38.19)	35.96 (39.50-32.75)	0.40%	0.39%	0.44%
	Jordan	38.42 (35.25-41.86)	42.45 (39.45-45.55)	43.70 (47.00-40.54)	0.45%	0.50%	0.32%
	Kuwait	41.10 (37.90-44.45)	44.10 (41.09-47.13)	45.33 (48.62-42.31)	0.34%	0.35%	0.31%
	Lebanon	41.32 (38.10-44.64)	44.49 (41.38-47.72)	47.14 (50.30-43.95)	0.46%	0.37%	0.65%
	Libya	28.56 (25.28-32.05)	33.99 (30.84-37.41)	35.69 (38.99-32.59)	0.77%	0.87%	0.54%
	Morocco	34.52 (31.30-37.98)	35.83 (32.57-39.39)	37.02 (40.53-33.85)	0.24%	0.19%	0.36%
	Oman	36.14 (32.99-39.53)	43.66 (40.80-46.70)	46.01 (49.32-42.78)	0.84%	0.95%	0.58%
	Palestine	38.18 (34.98-41.64)	38.04 (34.93-41.42)	39.59 (42.89-36.35)	0.13%	-0.02%	0.44%
	Qatar	32.91 (29.75-36.27)	33.99 (30.79-37.50)	33.33 (36.70-30.13)	0.04%	0.16%	-0.22%
	Saudi Arabia	24.02 (20.89-27.41)	33.46 (30.32-36.79)	35.33 (38.68-32.12)	1.34%	1.67%	0.61%
	Sudan	22.75 (19.44-26.14)	25.04 (21.83-28.53)	27.13 (30.62-23.91)	0.61%	0.48%	0.90%
	Syria	35.22 (31.93-38.66)	37.89 (34.60-41.22)	38.25 (41.63-34.98)	0.28%	0.37%	0.10%
	The UAE	40.81 (37.52-44.23)	48.95 (45.81-52.22)	49.84 (53.13-46.67)	0.69%	0.91%	0.20%
	Tunisia	34.56 (31.29-37.87)	36.95 (33.71-40.33)	37.85 (41.23-34.55)	0.31%	0.34%	0.27%
	Turkey	36.06 (32.86-39.35)	37.11 (34.18-40.31)	30.86 (34.22-27.83)	-0.54%	0.14%	-2.03%
	Yemen	30.34 (27.10-33.73)	33.43 (30.27-36.78)	33.87 (37.30-30.67)	0.38%	0.49%	0.14%

Supplementary Table 6

Risk factor	Location	DALY Rate 1990	DALY Rate 2010	DALY Rate 2019	ARC 1990-2019	ARC 1990-2010	ARC 2010-2019
High Systolic Blood Pressure	The NAME	5,887.94 (5,221.21-6,582.29)	4,808.89 (4,276.12-5,361.24)	4,401.89 (3,785.93-5,042.84)	-1.00%	-1.01%	-0.98%
	Afghanistan	9,186.51 (7,100.08-11,432.12)	8,061.58 (6,163.15-10,331.32)	7,400.80 (5,554.91-9,271.56)	-0.74%	-0.65%	-0.95%
	Algeria	6,958.67 (5,663.23-8,314.93)	4,294.65 (3,600.02-5,035.84)	3,927.64 (3,132.49-4,789.98)	-1.95%	-2.38%	-0.99%
	Bahrain	6,431.56 (5,370.19-7,560.64)	3,330.95 (2,820.71-3,894.79)	2,622.29 (2,110.86-3,205.01)	-3.05%	-3.24%	-2.62%
	Egypt	6,314.85 (5,392.76-7,203.58)	7,037.59 (6,071.49-7,975.89)	6,576.09 (4,975.86-8,308.12)	0.14%	0.54%	-0.75%
	Iran	4,800.22 (4,200.47-5,423.47)	3,299.30 (2,949.68-3,645.97)	2,973.39 (2,652.25-3,280.15)	-1.64%	-1.86%	-1.15%
	Iraq	7,130.70 (5,973.39-8,436.63)	6,545.79 (5,241.14-7,970.70)	6,025.44 (4,796.31-7,308.90)	-0.58%	-0.43%	-0.92%
	Jordan	5,417.26 (4,592.26-6,301.38)	3,557.55 (3,095.11-4,014.80)	3,228.75 (2,707.60-3,804.52)	-1.77%	-2.08%	-1.07%
	Kuwait	3,748.24 (3,277.46-4,207.51)	2,885.46 (2,584.24-3,167.06)	2,379.70 (1,967.15-2,861.56)	-1.55%	-1.30%	-2.12%
	Lebanon	5,295.50 (4,364.89-6,438.28)	4,020.15 (3,207.53-4,642.88)	3,904.36 (2,956.72-4,605.25)	-1.05%	-1.37%	-0.32%
	Libya	4,212.26 (3,440.24-5,106.86)	3,880.79 (3,483.16-4,355.29)	4,061.98 (3,223.62-5,040.70)	-0.13%	-0.41%	0.51%
	Morocco	6,962.00 (6,017.25-7,928.41)	6,083.74 (4,889.60-7,443.90)	5,955.25 (4,659.10-7,044.06)	-0.54%	-0.67%	-0.24%
	Oman	5,912.22 (4,456.83-7,656.70)	6,675.34 (5,958.43-7,436.20)	4,921.40 (4,161.25-5,715.35)	-0.63%	0.61%	-3.33%
	Palestine	5,499.91 (4,290.15-6,812.75)	4,263.02 (3,686.56-4,754.87)	3,963.97 (3,318.58-4,648.75)	-1.12%	-1.27%	-0.80%
	Qatar	5,707.93 (4,698.82-6,810.11)	4,180.25 (3,460.81-4,940.96)	3,215.68 (2,498.00-4,061.58)	-1.96%	-1.55%	-2.87%
	Saudi Arabia	4,907.21 (3,876.33-6,131.36)	5,449.85 (4,772.42-6,157.17)	4,430.78 (3,521.40-5,282.85)	-0.35%	0.53%	-2.27%
	Sudan	8,206.84 (6,668.87-9,926.50)	6,925.49 (5,292.95-8,658.11)	6,465.28 (5,167.47-8,161.93)	-0.82%	-0.85%	-0.76%
	Syria	6,561.11 (5,182.54-8,043.41)	5,050.78 (4,005.42-6,143.09)	5,126.62 (3,761.21-6,668.83)	-0.85%	-1.30%	0.17%
	The UAE	7,550.86 (6,332.89-9,072.38)	5,990.06 (4,943.52-7,005.66)	4,415.11 (3,446.46-5,592.66)	-1.83%	-1.15%	-3.33%
	Tunisia	4,013.30 (3,322.98-4,753.19)	3,591.73 (2,696.00-4,555.20)	3,312.73 (2,472.38-4,317.64)	-0.66%	-0.55%	-0.89%
	Turkey	4,667.00 (3,957.08-5,360.97)	2,964.07 (2,581.02-3,404.69)	2,503.26 (2,014.27-3,054.89)	-2.13%	-2.24%	-1.86%
	Yemen	6,804.89 (5,235.46-8,710.56)	5,682.44 (4,434.46-7,127.37)	5,737.53 (4,412.14-7,387.13)	-0.59%	-0.90%	0.11%
High Fasting Plasma Glucose	The NAME	2,624.17 (2,154.43-3,264.22)	3,180.34 (2,564.96-3,943.35)	3,332.73 (2,650.14-4,117.87)	0.83%	0.97%	0.52%
	Afghanistan	4,314.30 (3,209.10-5,668.91)	5,862.93 (4,450.70-7,687.30)	5,920.63 (4,418.61-7,702.53)	1.10%	1.55%	0.11%
	Algeria	2,798.12 (2,115.15-3,800.29)	3,129.04 (2,381.11-4,116.83)	3,206.25 (2,476.21-4,144.79)	0.47%	0.56%	0.27%
	Bahrain	6,261.77 (4,891.49-7,982.59)	5,794.30 (4,973.47-6,720.59)	5,121.64 (4,202.36-6,178.17)	-0.69%	-0.39%	-1.36%
	Egypt	2,311.51 (1,894.29-2,922.03)	3,600.40 (2,835.41-4,550.78)	4,369.04 (3,084.35-5,760.58)	2.22%	2.24%	2.17%
	Iran	1,988.06 (1,601.44-2,516.03)	2,275.79 (1,826.58-2,847.25)	2,511.20 (2,017.38-3,108.71)	0.81%	0.68%	1.10%
	Iraq	4,212.40 (3,364.83-5,303.99)	4,763.79 (3,644.07-6,151.66)	4,737.92 (3,650.18-5,900.26)	0.41%	0.62%	-0.06%
	Jordan	3,973.00 (3,269.12-4,986.94)	3,276.10 (2,739.91-3,919.23)	2,960.09 (2,379.65-3,643.72)	-1.01%	-0.96%	-1.12%
	Kuwait	3,043.66 (2,430.85-3,770.02)	2,764.69 (2,243.27-3,357.74)	2,556.53 (2,005.34-3,226.50)	-0.60%	-0.48%	-0.87%
	Lebanon	2,928.58 (2,190.04-3,918.11)	3,257.65 (2,400.26-4,220.40)	3,311.63 (2,448.91-4,266.77)	0.42%	0.53%	0.18%
	Libya	2,532.90 (1,913.83-3,400.88)	3,159.15 (2,487.46-3,961.21)	3,462.02 (2,627.86-4,581.71)	1.08%	1.11%	1.02%
	Morocco	2,244.02 (1,773.67-2,896.05)	3,547.02 (2,639.70-4,772.42)	4,049.56 (3,046.61-5,338.12)	2.06%	2.32%	1.48%
	Oman	3,857.71 (2,897.54-5,160.15)	5,289.86 (4,236.28-6,690.63)	4,620.40 (3,618.47-5,820.13)	0.62%	1.59%	-1.49%
	Palestine	4,163.64 (3,091.55-5,454.76)	4,373.08 (3,568.26-5,337.65)	4,667.61 (3,783.93-5,803.84)	0.39%	0.25%	0.73%
	Qatar	6,355.16 (5,097.32-7,994.86)	6,566.25 (5,449.27-7,841.13)	5,263.04 (4,206.33-6,505.17)	-0.65%	0.16%	-2.43%
	Saudi Arabia	3,388.18 (2,589.98-4,421.44)	4,118.76 (3,351.30-5,023.03)	3,765.48 (2,970.80-4,712.30)	0.36%	0.98%	-0.99%
	Sudan	2,619.53 (1,949.72-3,536.12)	3,593.21 (2,589.89-4,909.34)	3,837.30 (2,905.38-5,098.16)	1.33%	1.59%	0.73%
	Syria	3,110.57 (2,379.98-4,199.84)	3,667.34 (2,686.28-4,982.10)	4,015.89 (2,858.59-5,556.49)	0.88%	0.83%	1.01%
	The UAE	5,606.20 (4,484.39-7,171.96)	5,833.24 (4,735.98-7,151.64)	4,416.55 (3,464.78-5,546.02)	-0.82%	0.20%	-3.04%
	Tunisia	2,311.07 (1,760.94-3,060.18)	2,976.16 (2,166.45-3,870.70)	3,023.13 (2,197.82-3,972.13)	0.93%	1.27%	0.17%
	Turkey	2,583.67 (2,153.35-3,051.14)	2,433.29 (1,969.20-3,026.56)	2,074.26 (1,625.08-2,697.53)	-0.75%	-0.30%	-1.76%
	Yemen	2,071.79 (1,569.68-2,749.24)	2,688.65 (1,989.42-3,726.52)	3,045.57 (2,152.01-4,267.22)	1.34%	1.31%	1.39%

Supplementary Table 6

		DALY Rate 1990	DALY Rate 2010	DALY Rate 2019	ARC 1990-2019	ARC 1990-2010	ARC 2010-2019
High Body-Mass Index	The NAME	3,488.84 (2,262.50-4,798.46)	3,639.48 (2,547.89-4,768.69)	3,777.18 (2,692.63-4,943.28)	0.27%	0.21%	0.41%
	Afghanistan	3,923.72 (1,946.79-6,345.43)	4,240.20 (2,347.27-6,524.12)	5,098.61 (3,230.86-7,244.71)	0.91%	0.39%	2.07%
	Algeria	3,481.36 (2,158.88-5,095.66)	3,254.17 (2,219.07-4,450.55)	3,339.17 (2,312.75-4,478.52)	-0.14%	-0.34%	0.29%
	Bahrain	5,453.51 (3,688.71-7,355.30)	4,661.13 (3,275.85-6,018.30)	4,297.40 (2,997.09-5,612.61)	-0.82%	-0.78%	-0.90%
	Egypt	4,648.23 (2,981.32-6,435.55)	5,650.72 (3,858.81-7,324.49)	5,929.58 (3,973.10-8,107.81)	0.84%	0.98%	0.54%
	Iran	2,419.58 (1,497.57-3,414.66)	2,430.19 (1,699.18-3,194.78)	2,580.92 (1,845.13-3,337.35)	0.22%	0.02%	0.67%
	Iraq	5,512.65 (3,548.13-7,546.62)	4,987.60 (3,269.05-6,981.13)	4,793.11 (3,232.66-6,505.33)	-0.48%	-0.50%	-0.44%
	Jordan	4,595.67 (3,077.04-6,088.89)	3,870.19 (2,750.14-4,863.03)	3,701.63 (2,637.00-4,819.42)	-0.74%	-0.86%	-0.49%
	Kuwait	3,581.48 (2,514.78-4,602.40)	3,313.05 (2,415.14-4,163.38)	3,156.08 (2,248.53-4,046.22)	-0.44%	-0.39%	-0.54%
	Lebanon	3,499.35 (2,176.73-5,023.44)	3,273.75 (2,151.56-4,456.37)	3,488.60 (2,344.55-4,641.18)	-0.01%	-0.33%	0.71%
	Libya	3,132.16 (2,088.79-4,318.24)	3,675.79 (2,617.68-4,683.38)	3,920.96 (2,681.32-5,191.76)	0.78%	0.80%	0.72%
	Morocco	2,978.64 (1,810.24-4,339.53)	3,562.54 (2,235.65-5,061.28)	3,930.33 (2,543.03-5,491.46)	0.96%	0.90%	1.10%
	Oman	3,177.47 (1,788.46-4,868.13)	5,345.37 (3,706.28-6,909.80)	4,401.68 (3,087.83-5,712.61)	1.13%	2.63%	-2.14%
	Palestine	3,419.11 (1,948.04-5,287.67)	3,262.10 (2,078.87-4,511.74)	3,647.36 (2,444.55-4,973.12)	0.22%	-0.23%	1.25%
	Qatar	5,514.94 (3,644.22-7,352.24)	5,946.26 (4,265.26-7,576.69)	4,904.93 (3,498.19-6,432.22)	-0.40%	0.38%	-2.12%
	Saudi Arabia	3,486.02 (2,198.78-4,998.60)	5,101.59 (3,600.76-6,468.37)	4,771.54 (3,389.16-6,141.76)	1.09%	1.92%	-0.74%
	Sudan	2,760.80 (1,444.99-4,319.65)	3,454.64 (2,113.19-4,993.56)	3,862.43 (2,483.09-5,500.04)	1.16%	1.13%	1.25%
	Syria	4,102.09 (2,441.39-6,045.96)	3,768.26 (2,438.65-5,274.67)	3,994.50 (2,515.44-5,694.32)	-0.09%	-0.42%	0.65%
	The UAE	5,877.80 (4,059.13-7,909.40)	7,043.48 (5,199.40-8,937.18)	5,732.92 (4,111.71-7,383.33)	-0.09%	0.91%	-2.26%
	Tunisia	2,450.44 (1,547.96-3,540.63)	2,824.99 (1,822.48-4,050.59)	2,916.79 (1,907.41-4,177.20)	0.60%	0.71%	0.36%
	Turkey	3,470.25 (2,224.29-4,783.75)	2,754.51 (1,847.67-3,675.65)	2,662.62 (1,803.98-3,599.41)	-0.91%	-1.15%	-0.38%
	Yemen	1,839.85 (730.39-3,329.78)	2,317.76 (1,232.69-3,625.23)	2,595.64 (1,422.71-4,079.92)	1.19%	1.16%	1.27%
High LDL Cholesterol	The NAME	3,201.08 (2,628.85-3,822.83)	2,433.09 (1,987.76-2,917.64)	2,235.44 (1,767.81-2,767.81)	-1.23%	-1.36%	-0.94%
	Afghanistan	4,947.80 (3,651.43-6,468.07)	4,347.00 (3,238.59-5,780.43)	3,927.38 (2,902.51-5,165.97)	-0.79%	-0.65%	-1.12%
	Algeria	3,265.44 (2,429.31-4,293.63)	2,094.39 (1,557.58-2,727.91)	1,913.98 (1,362.60-2,580.77)	-1.83%	-2.20%	-1.00%
	Bahrain	4,582.30 (3,714.99-5,486.79)	1,827.82 (1,436.33-2,262.59)	1,388.97 (1,030.97-1,802.72)	-4.03%	-4.49%	-3.00%
	Egypt	3,826.30 (3,103.43-4,725.73)	3,700.47 (2,967.13-4,467.89)	3,560.60 (2,583.81-4,814.10)	-0.25%	-0.17%	-0.43%
	Iran	3,106.58 (2,528.43-3,789.11)	1,769.16 (1,432.16-2,147.46)	1,574.46 (1,268.65-1,922.44)	-2.32%	-2.78%	-1.29%
	Iraq	3,372.76 (2,653.41-4,220.17)	2,991.01 (2,198.60-3,899.24)	2,668.82 (1,966.51-3,511.53)	-0.80%	-0.60%	-1.26%
	Jordan	2,664.63 (2,109.52-3,337.76)	1,588.95 (1,297.43-1,975.75)	1,438.72 (1,109.08-1,845.95)	-2.10%	-2.55%	-1.10%
	Kuwait	2,292.02 (1,934.68-2,684.93)	1,656.53 (1,410.96-1,951.23)	1,401.78 (1,112.85-1,775.58)	-1.68%	-1.61%	-1.84%
	Lebanon	3,727.72 (2,991.30-4,545.03)	2,628.52 (1,983.27-3,210.97)	2,537.48 (1,828.55-3,159.21)	-1.32%	-1.73%	-0.39%
	Libya	1,878.22 (1,414.51-2,511.50)	1,786.46 (1,449.16-2,308.17)	1,947.06 (1,443.96-2,622.98)	0.12%	-0.25%	0.96%
	Morocco	3,213.52 (2,516.15-3,936.70)	2,820.38 (2,068.42-3,725.70)	2,806.21 (2,010.30-3,595.89)	-0.47%	-0.65%	-0.06%
	Oman	4,655.64 (3,433.03-6,016.60)	3,964.21 (3,256.96-4,818.33)	2,927.65 (2,253.54-3,705.08)	-1.59%	-0.80%	-3.31%
	Palestine	3,178.25 (2,375.10-4,138.45)	2,231.43 (1,817.03-2,735.27)	2,179.07 (1,696.39-2,806.35)	-1.29%	-1.75%	-0.26%
	Qatar	3,263.80 (2,441.17-4,198.31)	2,042.97 (1,497.85-2,694.03)	1,579.98 (1,069.87-2,162.70)	-2.47%	-2.32%	-2.82%
	Saudi Arabia	2,244.31 (1,588.10-2,973.36)	2,885.44 (2,362.76-3,463.67)	2,406.26 (1,815.69-3,037.56)	0.24%	1.26%	-2.00%
	Sudan	3,419.05 (2,474.54-4,565.90)	2,756.83 (1,870.78-3,754.85)	2,633.10 (1,788.48-3,623.92)	-0.90%	-1.07%	-0.51%
	Syria	4,243.11 (3,268.50-5,375.89)	3,393.32 (2,640.05-4,273.04)	3,423.14 (2,446.97-4,651.23)	-0.74%	-1.11%	0.10%
	The UAE	3,227.60 (2,426.83-4,261.65)	2,937.20 (2,227.38-3,844.29)	2,249.97 (1,645.74-3,008.72)	-1.24%	-0.47%	-2.92%
	Tunisia	2,225.12 (1,719.34-2,815.84)	2,006.74 (1,390.93-2,710.77)	1,846.97 (1,270.37-2,541.34)	-0.64%	-0.52%	-0.92%
	Turkey	2,572.63 (2,097.50-3,106.08)	1,338.64 (1,053.22-1,667.77)	1,060.78 (782.26-1,418.21)	-3.01%	-3.21%	-2.55%
	Yemen	3,857.75 (2,723.23-5,118.72)	3,117.18 (2,218.97-4,166.19)	3,219.57 (2,322.08-4,394.93)	-0.62%	-1.06%	0.36%

Supplementary Table 7

		High SBP		High FPG		High BMI		High LDL	
		Death	DALY	Death	DALY	Death	DALY	Death	DALY
Low SDI	Afghanistan	1.19	1.16	1.25	1.31	1.65	1.67	1.06	1.01
	Yemen	0.98	0.93	0.96	0.94	1.23	1.22	0.80	0.72
	Sudan	0.99	0.92	0.91	0.89	1.07	1.05	0.84	0.76
	Morocco	0.98	0.95	0.89	0.92	1.13	1.14	0.89	0.84
	Palestine	0.78	0.74	0.80	0.79	1.06	0.98	0.71	0.63
Low-middle SDI	Syria	1.09	0.86	1.07	0.91	1.14	0.99	1.01	0.77
	Algeria	1.28	1.08	1.19	1.07	1.40	1.23	1.20	0.96
	Egypt	1.35	1.10	1.43	1.19	1.35	1.13	1.19	0.92
	The NAME	0.97	0.88	0.96	0.93	1.08	1.01	0.86	0.75
Middle SDI	Iran	0.95	0.81	1.04	0.97	1.09	0.97	0.88	0.69
	Iraq	0.73	0.70	0.76	0.78	0.80	0.81	0.71	0.65
	Tunisia	0.83	0.77	0.72	0.71	0.95	0.90	0.66	0.59
	Lebanon	0.58	0.52	0.60	0.62	0.73	0.71	0.56	0.47
	Libya	0.99	0.96	0.94	0.95	1.13	1.09	0.89	0.84
High-middle SDI	Jordan	1.09	0.92	0.99	0.87	1.09	0.94	0.87	0.67
	Turkey	0.90	0.77	0.87	0.84	1.00	0.93	0.77	0.60
	Bahrain	1.10	1.02	1.01	0.97	1.20	1.10	0.99	0.85
	Oman	0.99	0.99	0.82	0.86	1.07	1.07	0.86	0.84
High SDI	Saudi Arabia	0.79	0.76	0.87	0.86	1.02	1.01	0.79	0.73
	Qatar	1.62	1.44	1.51	1.33	1.66	1.40	1.57	1.38
	Kuwait	0.65	0.56	0.63	0.66	0.74	0.73	0.42	0.33
	The UAE	0.86	0.79	0.86	0.85	0.86	0.86	0.79	0.66
