# Supplementary material for: The burden of metabolic risk factors in North Africa and the Middle East, 1990–2019: findings from the Global Burden of Disease Study
Source: eClinicalMedicine. 2023 Jun 2;60:102022. doi: 10.1016/j.eclinm.2023.102022 (PMC10242634; doi:10.1016/j.eclinm.2023.102022)
Supplement: Affiliations [file mmc3.docx]

# GBD 2019 NAME Metabolic Risk Factors Collaborators

Mohammad-Reza Malekpour, Mohsen Abbasi-Kangevari, Seyyed-Hadi Ghamari, Javad Khanali, Mahsa Heidari-Foroozan, Sahar Saeedi Moghaddam, Mohammadreza Azangou-Khyavy, Sahba Rezazadeh-Khadem, Negar Rezaei, Parnian Shobeiri, Zahra Esfahani, Nazila Rezaei, Amirali Aali, Sherief Abd-Elsalam, Meriem Abdoun, Abdorrahim Absalan, Eman Abu-Gharbieh, Niveen ME Abu-Rmeileh, Ahmed Abu-Zaid, Ali Ahmadi, Sepideh Ahmadi, Ayman Ahmed, Tarik Ahmed Rashid, Marjan Ajami, Mostafa Akbarzadeh-Khiavi, Hanadi Al Hamad, Tariq A Alalwan, Khalid F Alhabib, Yousef Alimohamadi, Vahid Alipour, Syed Mohamed Aljunid, Mahmoud A Alomari, Saleh A Alqahatni, Rajaa M Al-Raddadi, Javad Javad Aminian Dehkordi, Mehrdad Amir-Behghadami, Sohrab Amiri, Davood Anvari, Jalal Arabloo, Judie Arulappan, Ashokan Arumugam, Zahra Aryan, Mohammad Athar, Seyyed Shamsadin Athari, Abolfazl Avan, Sina Azadnajafabad, Samad Azari, Hosein Azizi, Nayereh Baghcheghi, Nader Bagheri, Sara Bagherieh, Ovidiu Constantin Baltatu, Akshaya Srikanth Bhagavathula, Vijayalakshmi S Bhojaraja, Souad Bouaoud, Muhammad Hammad Butt, Luciana Aparecida Campos, Abdulaal Chitheer, Reza Darvishi Cheshmeh Soltani, Aso Mohammad Darwesh, Shirin Djalalinia, Milad Dodangeh, Maysaa El Sayed Zaki, Iffat Elbarazi, Muhammed Elhadi, Waseem El-Huneidi, Rana Ezzeddini, Mohammad Fareed, Hossein Farrokhpour, Ali Fatehizadeh, Yaseen Galali, Amir Ghaderi, Mansour Ghafourifard, Mohammad Ghasemi Nour, Ahmad Ghashghaee, Maryam Gholamalizadeh, Pouya Goleij, Mohamad Golitaleb, Parham Habibzadeh, Nima Hafezi-Nejad, Rabih Halwani, Hamidreza Hasani, Maryam Hashemian, Amr Hassan, Soheil Hassanipour, Hadi Hassankhani, Kamal Hezam, Reza Homayounfar, Seyed Kianoosh Hosseini, Kaveh Hosseini, Mehdi Hosseinzadeh, Soodabeh Hoveidamanesh, Jalil Jaafari, Haitham Jahrami, Elham Jamshidi, Tahereh Javaheri, Sathish Kumar Jayapal, Ali Kabir, Amirali Karimi, Neda Kaydi, Mohammad Keykhaei, Yousef Saleh Khader, Morteza Abdullatif Khafaie, Moien AB Khan, Kashif Ullah Khan, Yusra H Khan, Moawiah Mohammad Khatatbeh, Farzad Kompani, Hamid Reza Koohestani, Mohammed Kuddus, Savita Lasrado, Sang-woong Lee, Soleiman Mahjoub, Ata Mahmoodpoor, Elham Mahmoudi, Elaheh Malakan Rad, Narges Malih, Ahmad Azam Malik, Tauqeer Hussain Mallhi, Yosef Manla, Borhan Mansouri, Mohammad Ali Mansournia, Parham Mardi, Abdoljalal Marjani, Sahar Masoudi, Entezar Mehrabi Nasab, Ritesh G Menezes, Vildan Mevsim, Yousef Mohammad, Mokhtar Mohammadi, Esmaeil Mohammadi, Noushin Mohammadifard, Arif Mohammed, Sara Momtazmanesh, Fateme Montazeri, Maryam Moradi, Maziar Moradi-Lakeh, Negar Morovatdar, Christopher J L Murray, Zuhair S Natto, Seyed Aria Nejadghaderi, Ali Nowroozi, Morteza Oladnabi, Ahmed Omar Bali, Emad Omer, Hamidreza Pazoki Toroudi, Raffaele Pezzani, Ashkan Pourabhari Langroudi, Sima Rafiei, Mehran Rahimi, Vafa Rahimi-Movaghar, Shayan Rahmani, Amir Masoud Rahmani, Vahid Rahmanian, Chythra R Rao, Sina Rashedi, Mohammad-Mahdi Rashidi, Reza Rawassizadeh, Elrashdy Moustafa Mohamed Redwan, Malihe Rezaee, Maryam Rezaei, Seyed Mohammad Riahi, Gholamreza Roshandel, Aly M A Saad, Maha Mohamed Saber-Ayad, Siamak Sabour, Leila Sabzmakan, Basema Saddik, Erfan Sadeghi, Saeid Sadeghian, Amirhossein Sahebkar, Morteza Saki, Saina Salahi, Sarvenaz Salahi, Amir Salek Farrokhi, Marwa Rashad Salem, Hamideh Salimzadeh, Abdallah M Samy, Nizal Sarrafzadegan, Brijesh Sathian, Melika Shafeghat, Syed Mahboob Shah, Jaffer Shah, Ataollah Shahbandi, Fariba Shahraki-Sanavi, Mehran Shams-Beyranvand, Mohd Shanawaz, Kiomars Sharafi, Javad Sharifi-Rad, Jeevan K Shetty, Zahra Shokri Varniab, Seyed Afshin Shorofi, Soraya Siabani, Mohammad Sadegh Soltani-Zangbar, Seidamir Pasha Tabaeian, Seyed-Amir Tabatabaeizadeh, Mohammad Tabish, Majid Taheri, Yasaman Taheri Abkenar, Moslem Taheri Soodejani, Amir Taherkhani, Arash Tehrani-Banihashemi, Mohamad-Hani Temsah, Bereket M Tigabu, Alireza Vakilian, Siavash Vaziri, Bay Vo, Fereshteh Yazdanpanah, Arzu Yigit, Vahit Yiğit, Mazyar Zahir, Burhan Abdullah Zaman, Maryam Zamanian, Moein Zangiabadian, Iman Zare, Zahra Zareshahrabadi, Ali H Mokdad, Mohsen Naghavi, Bagher Larijani, and Farshad Farzadfar.

# Affiliations

Non-Communicable Diseases Research Center, Endocrinology and Metabolism Population Sciences Institute (M Malekpour MD, M Abbasi-Kangevari MD, S Ghamari MD, J Khanali MD, M Heidari-Foroozan BMedSc, S Saeedi Moghaddam MSc, M Azangou-Khyavy MD, S Rezazadeh-Khadem MD, N Rezaei PhD, Z Esfahani MSc, N Rezaei MD, Z Aryan MD, S Azadnajafabad MD, H Farrokhpour MD, M Keykhaei MD, S Momtazmanesh MD, F Montazeri MD, S Nejadghaderi MD, A Pourabhari Langroudi MD, S Rahmani MD, M Rashidi MD, S Rashedi MD, Z Shokri Varniab MD, Prof F Farzadfar DSc), Endocrinology and Metabolism Research Center, Endocrinology and Metabolism Clinical Sciences Institute (N Rezaei PhD, Prof B Larijani FACE, Prof F Farzadfar DSc), School of Medicine (P Shobeiri MD, H Farrokhpour MD, N Hafezi-Nejad MD, A Karimi MD, E Mohammadi MD, S Momtazmanesh MD, A Nowroozi BMedSc, M Shafeghat MD, A Shahbandi MD), Department of Epidemiology and Biostatistics (H Azizi PhD, M Mansournia PhD), Digestive Diseases Research Institute (M Hashemian PhD, S Masoudi MSc, H Salimzadeh PhD), Tehran Heart Center (K Hosseini MD, E Mehrabi Nasab MD, M Rezaee MD), Department of Cardiology (K Hosseini MD, E Mahmoudi MD, S Rashedi MD), Students’ Scientific Research Center (SSRC) (M Keykhaei MD), Children's Medical Center (F Kompani MD), Department of Pediatric Cardiology (Prof E Malakan Rad MD), Sina Trauma and Surgery Research Center (Prof V Rahimi-Movaghar MD), Department of Pediatric Allergy and Immunology (F Yazdanpanah MD) Tehran University of Medical Sciences, Tehran, Iran;

Social Determinants of Health Research Center (S Ghamari MD, J Khanali MD, M Azangou-Khyavy MD, M Rashidi MD), Department of Medicine (M Heidari-Foroozan BMedSc), Department of Epidemiology (A Ahmadi PhD, S Sabour PhD), School of Advanced Technologies in Medicine (S Ahmadi PhD), Cancer Research Center (M Gholamalizadeh PhD), National Nutrition and Food Technology Research Institute (R Homayounfar PhD), Functional Neurosurgery Research Center (E Jamshidi PharmD), School of Medicine (F Montazeri MD, S Nejadghaderi MD, S Rahmani MD, M Zangiabadian MD), Pharmacology Department (M Rezaee MD), Medical Ethics and Law Research Center (M Taheri PhD), Urology and Nephrology Research Center (M Zahir MD), , Shahid Beheshti University of Medical Sciences, Tehran, Iran;

Faculty of Medicine (A Aali MD), Department of Public Health (A Avan MD), E-Learning Center (M Ghasemi Nour MD), Clinical Research Development Unit (N Morovatdar MD), Applied Biomedical Research Center (A Sahebkar PhD), Biotechnology Research Center (A Sahebkar PhD), Mashhad University of Medical Sciences, Mashhad, Iran;

Tropical Medicine Department (S Abd-Elsalam PhD), Tanta University, Tanta, Egypt;

Department of Medicine (Prof M Abdoun BMedSc), University of Setif Algeria, Sétif, Algeria;

Department of Medical Laboratory Sciences (A Absalan PhD), Khomein University of Medical Sciences, Khomein, Iran;

Department of Research and Development (A Absalan PhD), Satras Biotechnology Company, Tehran, Iran;

Clinical Sciences Department (E Abu-Gharbieh PhD, Prof R Halwani PhD), Department of Physiotherapy (A Arumugam PhD), Department of Basic Medical Sciences (W El-Huneidi PhD), College of Medicine (Prof R Halwani PhD), Department of Clinical Sciences (M M Saber-Ayad MD), Sharjah Institute for Medical Research (B Saddik PhD), University of Sharjah, Sharjah, United Arab Emirates;

Institute of Community and Public Health (Prof N M Abu-Rmeileh PhD), Birzeit University, Ramallah, Palestine;

Department of Surgery (A Abu-Zaid MD), Alfaisal University, Riyadh, Saudi Arabia;

College of Graduate Health Sciences (A Abu-Zaid MD), University of Tennessee, Memphis, TN, USA;

Department of Epidemiology and Biostatistics (A Ahmadi PhD), Basic Health Sciences Institute (N Bagheri PhD), Shahrekord University of Medical Sciences, Shahrekord, Iran;

Institute of Endemic Diseases (A Ahmed MSc), University of Khartoum, Khartoum, Sudan; Swiss Tropical and Public Health Institute (A Ahmed MSc), University of Basel, Basel, Switzerland;

Department of Computer Science and Engineering (T Ahmed Rashid PhD), University of Kurdistan Hewler, Erbil, Iraq;

Department of Food and Nutrition Policy and Planning Research (M Ajami PhD), National Institute of Nutrition, Tehran, Iran;

National Nutrition and Food Technology Research Institute (M Ajami PhD), Shahid Beheshti University of Medical Sciences, Iran;

Liver and Gastrointestinal Diseases Research Center (M Akbarzadeh-Khiavi PhD), Road Traffic Injury Research Center (M Amir-Behghadami MSc), Research Center of Psychiatry and Behavioral Sciences (H Azizi PhD), Department of Medical Surgical Nursing (M Ghafourifard PhD), School of Nursing and Midwifery (H Hassankhani PhD), Department of Anesthesiology and Critical Care (Prof A Mahmoodpoor MD), Cardiovascular Research Center (M Rahimi MD), Department of Immunology (M Soltani-Zangbar MSc), Department of Pediatric Allergy and Immunology (F Yazdanpanah MD), Tabriz University of Medical Sciences, Tabriz, Iran;

Geriatric and Long Term Care Department (H Al Hamad MD, B Sathian PhD), Rumailah Hospital (H Al Hamad MD), Hamad Medical Corporation, Doha, Qatar;

Department of Biology (T A Alalwan PhD), University of Bahrain, Sakhir, Bahrain;

Department of Cardiac Sciences (Prof K F Alhabib MD), Internal Medicine Department (Y Mohammad MD), Pediatric Intensive Care Unit (M Temsah MD), King Saud University, Riyadh, Saudi Arabia;

Health Research Center (Y Alimohamadi PhD), Quran and Hadith Research Center (S Amiri PhD), Baqiyatallah University of Medical Sciences, Tehran, Iran;

Health Management and Economics Research Center (V Alipour PhD, J Arabloo PhD), Department of Health Economics (V Alipour PhD), Hospital Management Research Center (S Azari PhD), School of Medicine (M Dodangeh MD), Minimally Invasive Surgery Research Center (A Kabir MD, S Salahi MD), Preventive Medicine and Public Health Research Center (M Moradi-Lakeh MD, A Tehrani-Banihashemi PhD), Department of Physiology (H Pazoki Toroudi PhD), Physiology Research Center (H Pazoki Toroudi PhD), Department of Internal Medicine (S Tabaeian MD), Trauma and Injury Research Center (M Taheri PhD), Department of Community and Family Medicine (A Tehrani-Banihashemi PhD), Iran University of Medical Sciences, Tehran, Iran (M Moradi MD);

Department of Health Policy and Management (Prof S M Aljunid PhD), Kuwait University, Kuwait, Kuwait;

International Centre for Casemix and Clinical Coding (Prof S M Aljunid PhD), National University of Malaysia, Bandar Tun Razak, Malaysia;

Department of Physical Education (Prof M A Alomari PhD), Qatar University, Doha, Qatar;

Department of Rehabilitation Sciences and Physical Therapy (Prof M A Alomari PhD), Department of Public Health (Prof Y S Khader PhD), Jordan University of Science and Technology, Irbid, Jordan;

Department of Medicine (S A Alqahatni MD), King Faisal Specialist Hospital & Research Center, Riyadh, Saudi Arabia;

Department of Medicine (S A Alqahatni MD), Department of Radiology and Radiological Science (N Hafezi-Nejad MD), Johns Hopkins University, Baltimore, MD, USA;

Department of Community Medicine (R M Al-Raddadi PhD), Rabigh Faculty of Medicine (A A Malik PhD), Department of Dental Public Health (Z S Natto DrPH), King Abdulaziz University, Jeddah, Saudi Arabia;

Department of Applied Science and Technology (J J Aminian Dehkordi PhD), University of California Berkeley, Berkeley, CA, USA;

Chamical Engineering Department (J J Aminian Dehkordi PhD), Department of Clinical Biochemistry (R Ezzeddini PhD), Tarbiat Modares University, Tehran, Iran;

Department of Health Service Management (M Amir-Behghadami MSc), Iranian Center of Excellence in Health Management, Tabriz, Iran;

Department of Parasitology (D Anvari PhD), Department of Medical-Surgical Nursing (S Shorofi PhD), Mazandaran University of Medical Sciences, Sari, Iran;

Department of Parasitology (D Anvari PhD), Iranshahr University of Medical Sciences, Iranshahr, Iran;

Department of Maternal and Child Health (J Arulappan DSc), Sultan Qaboos University, Muscat, Oman;

Community Medicine and Rehabilitation (A Arumugam PhD), Umeå University, Umea, Sweden;

Brigham and Women's Hospital (Z Aryan MD), Department of Health Policy and Oral Epidemiology (Z S Natto DrPH), Harvard University, Boston, MA, USA;

Department of Medical Genetics (M Athar PhD), Science and Technology Unit (M Athar PhD), Umm Al-Qura University, Makkah, Saudi Arabia;

Department of Immunology (S Athari PhD), Zanjan University of Medical Sciences, Zanjan, Iran;

Department of Nursing (N Baghcheghi PhD), Saveh University of Medical Sciences, saveh, Iran;

School of Medicine (S Bagherieh BSc), Department of Environmental Health Engineering (A Fatehizadeh PhD), Isfahan Cardiovascular Research Institute (N Mohammadifard PhD, Prof N Sarrafzadegan MD), Isfahan University of Medical Sciences, Isfahan, Iran;

Department of Pharmacology & Therapeutics (Prof O C Baltatu PhD), Khalifa University, Abu Dhabi, United Arab Emirates;

Center of Innovation, Technology and Education (CITE) (Prof O C Baltatu PhD, Prof L A Campos PhD), Anhembi Morumbi University, Sao Jose dos Campos, Brazil;

Department of Health, Human Performance, and Recreation (A S Bhagavathula PhD), University of Arkansas, Fayetteville, AR, USA;

Department of Anatomy (V S Bhojaraja MD), Department of Biochemistry (J K Shetty MD), Royal College of Surgeons in Ireland Medical University of Bahrain, Busaiteen, Bahrain;

Department of Medicine (Prof S Bouaoud MD), Ferhat Abbas University of Setif, Setif, Algeria;

Epidemiology and Preventive Medicine (Prof S Bouaoud MD), University Hospital Saadna Abdenour, Setif, Algeria;

Faculty of Pharmacy (M Butt MS), University of Central Punjab, Lahore, Pakistan;

College of Health Sciences (Prof L A Campos PhD), Abu Dhabi University, Abu Dhabi, United Arab Emirates;

Iraq Field Epidemiology Training Program (I-FETP) (A Chitheer MD), Ministry of Health, Baghdad, Iraq;

Department of Environmental Health (R Darvishi Cheshmeh Soltani PhD), Department of Nursing (M Golitaleb PhD), Department of Epidemiology (M Zamanian PhD), Arak University of Medical Sciences, Arak, Iran;

Department of Information Technology (A M Darwesh PhD), Department of Computer Science (M Hosseinzadeh PhD), Diplomacy and Public Relations Department (A Omar Bali PhD), University of Human Development, Sulaymaniyah, Iraq;

Development of Research and Technology Center (S Djalalinia PhD), Ministry of Health and Medical Education, Tehran, Iran;

Clinical Pathology Department (Prof M El Sayed Zaki PhD), Mansoura University, Mansoura, Egypt;

Institute of Public Health (I Elbarazi DrPH, Prof S M Shah PhD), Family Medicine Department (M A Khan MSc), United Arab Emirates University, Al Ain, United Arab Emirates;

Faculty of Medicine (M Elhadi MD), University of Tripoli, Tripoli, Libya;

College of Medicine (M Fareed PhD), Imam Mohammad Ibn Saud Islamic University, Riyadh, Saudi Arabia;

Food Technology Department (Y Galali ResM), Salahaddin University-Erbil, Erbil, Iraq;

Department of Nutrition and Dietetics (Y Galali ResM), Cihan University-Erbil, Erbil, Iraq;

Department of Addiction Studies (A Ghaderi PhD), Kashan University of Medical Sciences, Kashan, Iran;

School of Public Health (A Ghashghaee BSc), Social Determinants of Health Research Center (S Rafiei PhD), Qazvin University of Medical Sciences, Qazvin, Iran;

Department of Genetics (P Goleij MSc), Sana Institute of Higher Education, Sari, Iran;

Research Center for Health Sciences, Institute of Health (P Habibzadeh MD), Department of Medical Mycology and Parasitology (Z Zareshahrabadi PhD), Shiraz University of Medical Sciences, Shiraz, Iran;

Department of Ophthalmology (H Hasani MD), Iran University of Medical Sciences, Karaj, Iran;

Biology Department (M Hashemian PhD), Utica University, Utica, NY, USA;

Department of Neurology (A Hassan MD), Cairo University, Cairo, Egypt;

Gastrointestinal and Liver Diseases Research Center (S Hassanipour PhD), Caspian Digestive Disease Research Center (S Hassanipour PhD), Department of Environmental Health Engineering (J Jaafari PhD), Guilan University of Medical Sciences, Rasht, Iran;

Independent Consultant, Tabriz, Iran (H Hassankhani PhD);

Department of Applied Microbiology (K Hezam PhD), Taiz University, Taiz, Yemen;

Department of Microbiology (K Hezam PhD), Nankai University, Tianjin, China;

Non-Communicable Diseases Research Center (R Homayounfar PhD), Fasa University of Medical Sciences, Fasa, Iran;

Department of Interventional Cardiology (S Hosseini MD), Hamedan University of Medical Sciences, Hamadan, Iran;

Institute of Research and Development (M Hosseinzadeh PhD), Duy Tan University, Da Nang, Viet Nam;

Burn Research Center (S Hoveidamanesh MD), Shahid Motahari Hospital, Tehran, Iran;

College of Medicine and Medical Sciences (H Jahrami PhD), Arabian Gulf University, Manama, Bahrain; Ministry of Health (H Jahrami PhD), Ministry of Health, Manama, Bahrain;

Division of Pulmonary Medicine (E Jamshidi PharmD), Lausanne University Hospital (CHUV), Lausanne, Switzerland;

Health Informatic Lab (T Javaheri PhD), Department of Computer Science (R Rawassizadeh PhD), Boston University, Boston, MA, USA;

Centre of Studies and Research (S Jayapal PhD), Ministry of Health, Muscat, Oman;

Environmental Health Department (N Kaydi PhD), Social Determinants of Health Research Center (M A Khafaie PhD), Department of Pediatric Neurology (S Sadeghian MD), Department of Microbiology (M Saki PhD), Ahvaz Jundishapur University of Medical Sciences, Ahvaz, Iran;

Primary Care Department (M A Khan MSc), NHS North West London, London, UK;

Department of Clinical Pharmacy (K Khan PhD), Department of Biochemistry (Prof M Kuddus PhD), University of Hail, Hail, Saudi Arabia;

Clinical Pharmacy Department (Y H Khan PhD), Department of Clinical Pharmacy (T Mallhi PhD), Jouf University, Sakaka, Saudi Arabia;

Department of Basic Medical Sciences (M M Khatatbeh PhD), Yarmouk University, Irbid, Jordan;

Social Determinants of Health Research Center (H Koohestani PhD), Saveh University of Medical Sciences, Saveh, Iran;

Department of Otorhinolaryngology (S Lasrado MS), Father Muller Medical College, Mangalore, India;

Pattern Recognition and Machine Learning Lab (Prof S Lee PhD), Gachon University, Seongnam, South Korea;

Cellular and Molecular Biology Research Center (Prof S Mahjoub PhD), Department of Clinical Biochemistry (Prof S Mahjoub PhD), Babol University of Medical Sciences, Babol, Iran;

Research Group on Global Health and Human Development (N Malih MD), University of the Balearic Islands (UIB), Palma, Spain;

University Institute of Public Health (A A Malik PhD), The University of Lahore, Lahore, Pakistan;

Heart and Vascular Institute (Y Manla MD), Cleveland Clinic Abu Dhabi, Abu Dhabi, United Arab Emirates;

Substance Abuse Prevention Research Center (B Mansouri PhD), Research Center for Environmental Determinants of Health (K Sharafi PhD), Department of Health Education and Health Promotion (S Siabani PhD), Department of Infectious Disease (Prof S Vaziri MD), Kermanshah University of Medical Sciences, Kermanshah, Iran;

Non-Communicable Diseases Research Center (P Mardi MD, L Sabzmakan PhD), School of Medicine (M Shams-Beyranvand MSc), Alborz University of Medical Sciences, Karaj, Iran;

Department of Biochemistry (A Marjani PhD), Gorgan Congenital Malformations Research Center (M Oladnabi PhD), Golestan Research Center of Gastroenterology and Hepatology (G Roshandel PhD), Golestan University of Medical Sciences, Gorgan, Iran;

Forensic Medicine Division (Prof R G Menezes MD), Imam Abdulrahman Bin Faisal University, Dammam, Saudi Arabia;

Department of Family Medicine (Prof V Mevsim PhD), Dokuz Eylul University, Izmir, Türkiye;

Department of Research and Development (Prof V Mevsim PhD), Vinnova Health Innovation CO., Izmir, Türkiye;

Department of Information Technology (M Mohammadi PhD), Lebanese French University, Erbil, Iraq;

Department of Biology (A Mohammed PhD), University of Jeddah, Jeddah, Saudi Arabia;

Institute for Health Metrics and Evaluation (Prof C J L Murray DPhil, Prof A H Mokdad PhD, Prof M Naghavi PhD), Department of Health Metrics Sciences, School of Medicine (Prof C J L Murray DPhil, Prof A H Mokdad PhD, Prof M Naghavi PhD), University of Washington, Seattle, WA, USA;

Mass Communication Department (E Omer PhD), Ajman University, Dubai, United Arab Emirates;

Department of Medicine (R Pezzani PhD), University of Padova, Padova, Italy;

Associazione Italiana Ricerca Oncologica di Base (AIROB), Padova, Italy (R Pezzani PhD);

Future Technology Research Center (A Rahmani PhD), National Yunlin University of Science and Technology, Yunlin, Taiwan;

Department of Community Medicine (V Rahmanian PhD), Jahrom University of Medical Sciences, Jahrom, Iran;

Department of Community Medicine (C R Rao MD), Manipal Academy of Higher Education, Manipal, India;

Department Biological Sciences (Prof E M M Redwan PhD), King Abdulaziz University, Jeddah, Egypt;

Department of Protein Research (Prof E M M Redwan PhD), Research and Academic Institution, Alexandria, Egypt;

Medical Toxicology and Drug Abuse Research Center (M Rezaei MD), Cardiovascular Diseases Research Center (S Riahi PhD), Birjand University of Medical Sciences, Birjand, Iran;

Cardiovascular Department (Prof A M A Saad MD), Zagazig University, Egypt, Zagazig, Egypt;

Department of Medical Pharmacology (M M Saber-Ayad MD), Public Health and Community Medicine Department (M R Salem MD), Cairo University, Giza, Egypt;

Research Consultation Center (RCC) (E Sadeghi PhD), Shiraz University of Medical Sciences, Iran;

Medical Laboratory (S Salahi BMedSc), Azad University of Medical Sciences, Tehran, Iran;

Advanced Therapy Medicinal Products Department (S Salahi MD), Royan Institution, Tehran, Iran;

Department of Immunology (A Salek Farrokhi PhD), Semnan University of Medical Sciences and Health Services, Semnan, Iran;

Department of Entomology (A M Samy PhD), Ain Shams University, Cairo, Egypt;

School of Population and Public Health (Prof N Sarrafzadegan MD), University of British Columbia, Vancouver, BC, Canada;

Faculty of Health and Social Sciences (B Sathian PhD), Bournemouth University, Bournemouth, UK;

Department of Family Medicine (Prof S M Shah PhD), Aga Khan University, Karachi, Pakistan;

Medical Research Center (J Shah BS), Kateb University, Kabul, Afghanistan;

Health Promotion Research Center (F Shahraki-Sanavi PhD), Zahedan University of Medical Sciences, Zahedan, Iran;

Department of Health Education and Promotion (M Shanawaz MD), Jazan University, Jazan, Saudi Arabia;

Facultad de Medicina (Faculty of Medicine) (J Sharifi-Rad PhD), Universidad del Azuay (University of Azuay), Cuenca, Ecuador;

Department of Nursing and Health Sciences (S Shorofi PhD), Flinders University, Adelaide, SA, Australia;

School of Health (S Siabani PhD), University of Technology Sydney, Sydney, NSW, Australia;

Department of Nutrition Sciences (S Tabatabaeizadeh PhD), Varastegan Institute for Medical Sciences, Mashhad, Iran;

Department of Pharmacology (M Tabish MPharm), Shaqra University, Shaqra, Saudi Arabia;

Living Systems Institute (Y Taheri Abkenar PharmD), University of Exeter, Exeter, UK;

Department of Biostatistics and Epidemiology (M Taheri Soodejani PhD), Shahid Sadoughi University of Medical Sciences, Yazd, Iran;

Research Center for Molecular Medicine (A Taherkhani PhD), Hamadan University of Medical Sciences, Hamadan, Iran;

Department of Pharmacy (B M Tigabu PhD), Komar University of Science and Technology, Sulaymaniyah, Iraq;

Department of Neurology (A Vakilian MD), Non-Communicable Diseases Research Center (A Vakilian MD), Rafsanjan University of Medical Sciences, Rafsanjan, Iran;

Faculty of Information Technology (B Vo PhD), Ho Chi Minh City University of Technology (HUTECH), Ho Chi Minh City, Viet Nam;

Department of Health Management (A Yigit PhD, V Yiğit PhD), Süleyman Demirel Üniversitesi (Süleyman Demirel University), Isparta, Türkiye;

Department of Pharmacology (B A Zaman MSc), University of Duhok, Duhok, Iraq;

Research and Development Department (I Zare BSc), Sina Medical Biochemistry Technologies, Shiraz, Iran.
