# Supplementary material for: The burden of metabolic risk factors in North Africa and the Middle East, 1990–2019: findings from the Global Burden of Disease Study
Source: eClinicalMedicine. 2023 Jun 2;60:102022. doi: 10.1016/j.eclinm.2023.102022 (PMC10242634; doi:10.1016/j.eclinm.2023.102022)
Supplement: Contributions [file mmc4.docx]

## Authors’ Contributions

## Providing data or critical feedback on data sources

Abdorrahim Absalan, Niveen ME Abu-Rmeileh, Ali Ahmadi, Sepideh Ahmadi, Ayman Ahmed, Tarik Ahmed Rashid, Mostafa Akbarzadeh-Khiavi, Hanadi Al Hamad, Khalid F Alhabib, Syed Mohamed Aljunid, Davood Anvari, Jalal Arabloo, Judie Arulappan, Zahra Aryan, Seyyed Shamsadin Athari, Samad Azari, Sara Bagherieh, Ovidiu Constantin Baltatu, Akshaya Srikanth Bhagavathula, Souad Bouaoud, Muhammad Hammad Butt, Abdulaal Chitheer, Aso Mohammad Darwesh, Maysaa El Sayed Zaki, Waseem El-Huneidi, Mohammad Fareed, Hossein Farrokhpour, Farshad Farzadfar, Ali Fatehizadeh, Yaseen Galali, Amir Ghaderi, Mansour Ghafourifard, Ahmad Ghashghaee, Pouya Goleij, Nima Hafezi-Nejad, Rabih Halwani, Maryam Hashemian, Soheil Hassanipour, Hadi Hassankhani, Reza Homayounfar, Kaveh Hosseini, Mehdi Hosseinzadeh, Jalil Jaafari, Haitham Jahrami, Tahereh Javaheri, Sathish Kumar Jayapal, Neda Kaydi, Yousef Saleh Khader, Morteza Abdullatif Khafaie, Moien AB Khan, Savita Lasrado, Sang-woong Lee, Ata Mahmoodpoor, Tauqeer Hussain Mallhi, Borhan Mansouri, Mohammad Ali Mansournia, Abdoljalal Marjani, Entezar Mehrabi Nasab, Ritesh G Menezes, Mokhtar Mohammadi, Noushin Mohammadifard, Ali H Mokdad, Sara Momtazmanesh, Maryam Moradi, Maziar Moradi-Lakeh, Christopher J L Murray, Mohsen Naghavi, Zuhair S Natto, Ahmed Omar Bali, Emad Omer, Sima Rafiei, Mehran Rahimi, Vafa Rahimi-Movaghar, Amir Masoud Rahmani, Shayan Rahmani, Chythra R Rao, Reza Rawassizadeh, Maryam Rezaei, Seyed Mohammad Riahi, Gholamreza Roshandel, Aly M A Saad, Siamak Sabour, Basema Saddik, Sahar Saeedi Moghaddam, Morteza Saki, Marwa Rashad Salem, Abdallah M Samy, Brijesh Sathian, Jaffer Shah, Mohd Shanawaz, Javad Sharifi-Rad, Parnian Shobeiri, Soraya Siabani, Mohammad Sadegh Soltani-Zangbar, Mohammad Tabish, Yasaman Taheri Abkenar, Amir Taherkhani, Mohamad-Hani Temsah, Bay Vo, Burhan Abdullah Zaman, Iman Zare, and Zahra Zareshahrabadi.

## Developing methods or computational machinery

Ali Ahmadi, Tarik Ahmed Rashid, Davood Anvari, Zahra Aryan, Souad Bouaoud, Aso Mohammad Darwesh, Milad Dodangeh, Farshad Farzadfar, Ali Fatehizadeh, Amir Ghaderi, Mehdi Hosseinzadeh, Tahereh Javaheri, Sang-woong Lee, Borhan Mansouri, Mokhtar Mohammadi, Ali H Mokdad, Christopher J L Murray, Mohsen Naghavi, Emad Omer, Amir Masoud Rahmani, Reza Rawassizadeh, Maryam Rezaei, Seyed Mohammad Riahi, Sahar Saeedi Moghaddam, Abdallah M Samy, Javad Sharifi-Rad, Mohammad Tabish, Yasaman Taheri Abkenar, and Bay Vo.

## Providing critical feedback on methods or results

Amirali Aali, Mohsen Abbasi-Kangevari, Sherief Abd-Elsalam, Meriem Abdoun, Eman Abu-Gharbieh, Niveen ME Abu-Rmeileh, Ahmed Abu-Zaid, Ali Ahmadi, Ayman Ahmed, Tarik Ahmed Rashid, Hanadi Al Hamad, Yousef Alimohamadi, Vahid Alipour, Syed Mohamed Aljunid, Mahmoud A. Alomari, Saleh A. Alqahatni, Rajaa M Al-Raddadi, Javad Aminian Dehkordi, Mehrdad Amir-Behghadami, Sohrab Amiri, Davood Anvari, Jalal Arabloo, Judie Arulappan, Ashokan Arumugam, Zahra Aryan, Mohammad Athar, Seyyed Shamsadin Athari, Abolfazl Avan, Sina Azadnajafabad, Mohammadreza Azangou-Khyavy, Samad Azari, Hosein Azizi, Nayereh Baghcheghi, Nader Bagheri, Sara Bagherieh, Ovidiu Constantin Baltatu, Akshaya Srikanth Bhagavathula, Vijayalakshmi S Bhojaraja, Souad Bouaoud, Muhammad Hammad Butt, Luciana Aparecida Campos, Abdulaal Chitheer, Reza Darvishi Cheshmeh Soltani, Aso Mohammad Darwesh, Shirin Djalalinia, Milad Dodangeh, Maysaa El Sayed Zaki, Muhammed Elhadi, Waseem El-Huneidi, Zahra Esfahani, Rana Ezzeddini, Mohammad Fareed, Hossein Farrokhpour, Farshad Farzadfar, Ali Fatehizadeh, Yaseen Galali, Amir Ghaderi, Mansour Ghafourifard, Seyyed-Hadi Ghamari, Mohammad Ghasemi Nour, Ahmad Ghashghaee, Maryam Gholamalizadeh, Mohamad Golitaleb, Parham Habibzadeh, Nima Hafezi-Nejad, Rabih Halwani, Hamidreza Hasani, Maryam Hashemian, Soheil Hassanipour, Hadi Hassankhani, Mahsa Heidari-Foroozan, Kamal Hezam, Reza Homayounfar, Seyed Kianoosh Hosseini, Mehdi Hosseinzadeh, Soodabeh Hoveidamanesh, Jalil Jaafari, Haitham Jahrami, Elham Jamshidi, Tahereh Javaheri, Sathish Kumar Jayapal, Ali Kabir, Amirali Karimi, Neda Kaydi, Mohammad Keykhaei, Yousef Saleh Khader, Morteza Abdullatif Khafaie, Kashif Ullah Khan, Moien AB Khan, Yusra H Khan, Javad Khanali, Moawiah Mohammad Khatatbeh, Farzad Kompani, Hamid Reza Koohestani, Mohammed Kuddus, Bagher Larijani, Savita Lasrado, Sang-woong Lee, Ata Mahmoodpoor, Elham Mahmoudi, Elaheh Malakan Rad, Mohammad-Reza Malekpour, Narges Malih, Ahmad Azam Malik, Tauqeer Hussain Mallhi, Yosef Manla, Borhan Mansouri, Mohammad Ali Mansournia, Parham Mardi, Abdoljalal Marjani, Sahar Masoudi, Entezar Mehrabi Nasab, Ritesh G Menezes, Vildan Mevsim, Yousef Mohammad, Esmaeil Mohammadi, Mokhtar Mohammadi, Arif Mohammed, Ali H Mokdad, Sara Momtazmanesh, Fateme Montazeri, Maryam Moradi, Maziar Moradi-Lakeh, Negar Morovatdar, Christopher J L Murray, Mohsen Naghavi, Zuhair S Natto, Seyed Aria Nejadghaderi, Ali Nowroozi, Ahmed Omar Bali, Emad Omer, Raffaele Pezzani, Mehran Rahimi, Vafa Rahimi-Movaghar, Amir Masoud Rahmani, Shayan Rahmani, Vahid Rahmanian, Chythra R Rao, Sina Rashedi, Mohammad-Mahdi Rashidi, Reza Rawassizadeh, Malihe Rezaee, Maryam Rezaei, Nazila Rezaei, Negar Rezaei, Sahba Rezazadeh-Khadem, Seyed Mohammad Riahi, Gholamreza Roshandel, Aly M A Saad, Maha Mohamed Saber-Ayad, Siamak Sabour, Leila Sabzmakan, Basema Saddik, Erfan Sadeghi, Saeid Sadeghian, Sahar Saeedi Moghaddam, Amir Salek Farrokhi, Marwa Rashad Salem, Hamideh Salimzadeh, Abdallah M Samy, Nizal Sarrafzadegan, Brijesh Sathian, Jaffer Shah, Syed Mahboob Shah, Ataollah Shahbandi, Mehran Shams-Beyranvand, Mohd Shanawaz, Kiomars Sharafi, Javad Sharifi-Rad, Jeevan K Shetty, Parnian Shobeiri, Zahra Shokri Varniab, Seyed Afshin Shorofi, Soraya Siabani, Mohammad Sadegh Soltani-Zangbar, Seidamir Pasha Tabaeian, Seyed-Amir Tabatabaeizadeh, Mohammad Tabish, Majid Taheri, Yasaman Taheri Abkenar, Moslem Taheri Soodejani, Arash Tehrani-Banihashemi, Mohamad-Hani Temsah, Bereket M Tigabu, Alireza Vakilian, Siavash Vaziri, Bay Vo, Fereshteh Yazdanpanah, Vahit YiÄŸit, Arzu Yigit, Mazyar Zahir, Burhan Abdullah Zaman, Maryam Zamanian, and Moein Zangiabadian.

## Drafting the work or revising is critically for important intellectual content

Amirali Aali, Mohsen Abbasi-Kangevari, Sherief Abd-Elsalam, Eman Abu-Gharbieh, Ahmed Abu-Zaid, Ali Ahmadi, Sepideh Ahmadi, Ayman Ahmed, Marjan Ajami, Mostafa Akbarzadeh-Khiavi, Tariq A. Alalwan, Mahmoud A. Alomari, Saleh A. Alqahatni, Mehrdad Amir-Behghadami, Sohrab Amiri, Jalal Arabloo, Judie Arulappan, Ashokan Arumugam, Seyyed Shamsadin Athari, Abolfazl Avan, Sina Azadnajafabad, Mohammadreza Azangou-Khyavy, Nader Bagheri, Sara Bagherieh, Ovidiu Constantin Baltatu, Akshaya Srikanth Bhagavathula, Vijayalakshmi S Bhojaraja, Souad Bouaoud, Muhammad Hammad Butt, Luciana Aparecida Campos, Milad Dodangeh, Maysaa El Sayed Zaki, Iffat Elbarazi, Muhammed Elhadi, Zahra Esfahani, Rana Ezzeddini, Farshad Farzadfar, Ali Fatehizadeh, Yaseen Galali, Amir Ghaderi, Mansour Ghafourifard, Seyyed-Hadi Ghamari, Mohammad Ghasemi Nour, Mohamad Golitaleb, Parham Habibzadeh, Nima Hafezi-Nejad, Rabih Halwani, Amr Hassan, Mahsa Heidari-Foroozan, Kamal Hezam, Reza Homayounfar, Kaveh Hosseini, Seyed Kianoosh Hosseini, Haitham Jahrami, Sathish Kumar Jayapal, Ali Kabir, Kashif Ullah Khan, Moien AB Khan, Yusra H Khan, Javad Khanali, Moawiah Mohammad Khatatbeh, Farzad Kompani, Mohammed Kuddus, Bagher Larijani, Savita Lasrado, Soleiman Mahjoub, Ata Mahmoodpoor, Elham Mahmoudi, Elaheh Malakan Rad, Mohammad-Reza Malekpour, Narges Malih, Ahmad Azam Malik, Tauqeer Hussain Mallhi, Borhan Mansouri, Parham Mardi, Entezar Mehrabi Nasab, Ritesh G Menezes, Vildan Mevsim, Yousef Mohammad, Esmaeil Mohammadi, Arif Mohammed, Ali H Mokdad, Sara Momtazmanesh, Fateme Montazeri, Maryam Moradi, Maziar Moradi-Lakeh, Christopher J L Murray, Mohsen Naghavi, Zuhair S Natto, Seyed Aria Nejadghaderi, Ali Nowroozi, Morteza Oladnabi, Hamidreza Pazoki Toroudi, Ashkan Pourabhari Langroudi, Mehran Rahimi, Vafa Rahimi-Movaghar, Shayan Rahmani, Chythra R Rao, Sina Rashedi, Elrashdy Moustafa Mohamed Redwan, Malihe Rezaee, Maryam Rezaei, Nazila Rezaei, Negar Rezaei, Sahba Rezazadeh-Khadem, Seyed Mohammad Riahi, Aly M A Saad, Maha Mohamed Saber-Ayad, Siamak Sabour, Leila Sabzmakan, Basema Saddik, Sahar Saeedi Moghaddam, Amirhossein Sahebkar, Saina Salahi, Sarvenaz Salahi, Amir Salek Farrokhi, Marwa Rashad Salem, Hamideh Salimzadeh, Abdallah M Samy, Melika Shafeghat, Syed Mahboob Shah, Fariba Shahraki-Sanavi, Mehran Shams-Beyranvand, Mohd Shanawaz, Javad Sharifi-Rad, Jeevan K Shetty, Parnian Shobeiri, Seyed Afshin Shorofi, Seidamir Pasha Tabaeian, Seyed-Amir Tabatabaeizadeh, Mohammad Tabish, Majid Taheri, Yasaman Taheri Abkenar, Alireza Vakilian, Vahit YiÄŸit, Arzu Yigit, Mazyar Zahir, Burhan Abdullah Zaman, Maryam Zamanian, and Iman Zare.

## Management of the publication or estimation process

Mohsen Abbasi-Kangevari, Farshad Farzadfar, Mohammad-Reza Malekpour, Ali H Mokdad, Christopher J L Murray, Mohsen Naghavi, and Sahar Saeedi Moghaddam.
