# Supplementary material for: The burden of metabolic risk factors in North Africa and the Middle East, 1990–2019: findings from the Global Burden of Disease Study
Source: eClinicalMedicine. 2023 Jun 2;60:102022. doi: 10.1016/j.eclinm.2023.102022 (PMC10242634; doi:10.1016/j.eclinm.2023.102022)
Supplement: PubMed_Table [file mmc5.docx]

# GBD 2019 NAME Metabolic Risk Factors Collaborators

| **First Name** | **Last Name** |
| --- | --- |
| Mohammad-Reza | Malekpour |
| Mohsen | Abbasi-Kangevari |
| Seyyed-Hadi | Ghamari |
| Javad | Khanali |
| Mahsa | Heidari-Foroozan |
| Sahar | Saeedi Moghaddam* |
| Mohammadreza | Azangou-Khyavy |
| Sahba | Rezazadeh-Khadem |
| Negar | Rezaei |
| Parnian | Shobeiri |
| Zahra | Esfahani |
| Nazila | Rezaei |
| Amirali | Aali |
| Sherief | Abd-Elsalam |
| Meriem | Abdoun |
| Abdorrahim | Absalan |
| Eman | Abu-Gharbieh |
| Niveen ME | Abu-Rmeileh |
| Ahmed | Abu-Zaid |
| Ali | Ahmadi |
| Sepideh | Ahmadi |
| Ayman | Ahmed |
| Tarik | Ahmed Rashid |
| Marjan | Ajami |
| Mostafa | Akbarzadeh-Khiavi |
| Hanadi | Al Hamad |
| Tariq A | Alalwan |
| Khalid F | Alhabib |
| Yousef | Alimohamadi |
| Vahid | Alipour |
| Syed Mohamed | Aljunid |
| Mahmoud A | Alomari |
| Saleh A | Alqahatni |
| Rajaa M | Al-Raddadi |
| Javad Javad | Aminian Dehkordi |
| Mehrdad | Amir-Behghadami |
| Sohrab | Amiri |
| Davood | Anvari |
| Jalal | Arabloo |
| Judie | Arulappan |
| Ashokan | Arumugam |
| Zahra | Aryan |
| Mohammad | Athar |
| Seyyed Shamsadin | Athari |
| Abolfazl | Avan |
| Sina | Azadnajafabad |
| Samad | Azari |
| Hosein | Azizi |
| Nayereh | Baghcheghi |
| Nader | Bagheri |
| Sara | Bagherieh |
| Ovidiu Constantin | Baltatu |
| Akshaya Srikanth | Bhagavathula |
| Vijayalakshmi S | Bhojaraja |
| Souad | Bouaoud |
| Muhammad Hammad | Butt |
| Luciana Aparecida | Campos |
| Abdulaal | Chitheer |
| Reza | Darvishi Cheshmeh Soltani |
| Aso Mohammad | Darwesh |
| Shirin | Djalalinia |
| Milad | Dodangeh |
| Maysaa | El Sayed Zaki |
| Iffat | Elbarazi |
| Muhammed | Elhadi |
| Waseem | El-Huneidi |
| Rana | Ezzeddini |
| Mohammad | Fareed |
| Hossein | Farrokhpour |
| Ali | Fatehizadeh |
| Yaseen | Galali |
| Amir | Ghaderi |
| Mansour | Ghafourifard |
| Mohammad | Ghasemi Nour |
| Ahmad | Ghashghaee |
| Maryam | Gholamalizadeh |
| Pouya | Goleij |
| Mohamad | Golitaleb |
| Parham | Habibzadeh |
| Nima | Hafezi-Nejad |
| Rabih | Halwani |
| Hamidreza | Hasani |
| Maryam | Hashemian |
| Amr | Hassan |
| Soheil | Hassanipour |
| Hadi | Hassankhani |
| Kamal | Hezam |
| Reza | Homayounfar |
| Seyed Kianoosh | Hosseini |
| Kaveh | Hosseini |
| Mehdi | Hosseinzadeh |
| Soodabeh | Hoveidamanesh |
| Jalil | Jaafari |
| Haitham | Jahrami |
| Elham | Jamshidi |
| Tahereh | Javaheri |
| Sathish Kumar | Jayapal |
| Ali | Kabir |
| Amirali | Karimi |
| Neda | Kaydi |
| Mohammad | Keykhaei |
| Yousef Saleh | Khader |
| Morteza Abdullatif | Khafaie |
| Moien AB | Khan |
| Kashif Ullah | Khan |
| Yusra H | Khan |
| Moawiah Mohammad | Khatatbeh |
| Farzad | Kompani |
| Hamid Reza | Koohestani |
| Mohammed | Kuddus |
| Savita | Lasrado |
| Sang-woong | Lee |
| Soleiman | Mahjoub |
| Ata | Mahmoodpoor |
| Elham | Mahmoudi |
| Elaheh | Malakan Rad |
| Narges | Malih |
| Ahmad Azam | Malik |
| Tauqeer Hussain | Mallhi |
| Yosef | Manla |
| Borhan | Mansouri |
| Mohammad Ali | Mansournia |
| Parham | Mardi |
| Abdoljalal | Marjani |
| Sahar | Masoudi |
| Entezar | Mehrabi Nasab |
| Ritesh G | Menezes |
| Vildan | Mevsim |
| Yousef | Mohammad |
| Mokhtar | Mohammadi |
| Esmaeil | Mohammadi |
| Noushin | Mohammadifard |
| Arif | Mohammed |
| Sara | Momtazmanesh |
| Fateme | Montazeri |
| Maryam | Moradi |
| Maziar | Moradi-Lakeh |
| Negar | Morovatdar |
| Christopher J L | Murray |
| Zuhair S | Natto |
| Seyed Aria | Nejadghaderi |
| Ali | Nowroozi |
| Morteza | Oladnabi |
| Ahmed | Omar Bali |
| Emad | Omer |
| Hamidreza | Pazoki Toroudi |
| Raffaele | Pezzani |
| Ashkan | Pourabhari Langroudi |
| Sima | Rafiei |
| Mehran | Rahimi |
| Vafa | Rahimi-Movaghar |
| Shayan | Rahmani |
| Amir Masoud | Rahmani |
| Vahid | Rahmanian |
| Chythra R | Rao |
| Sina | Rashedi |
| Mohammad-Mahdi | Rashidi |
| Reza | Rawassizadeh |
| Elrashdy Moustafa Mohamed | Redwan |
| Malihe | Rezaee |
| Maryam | Rezaei |
| Seyed Mohammad | Riahi |
| Gholamreza | Roshandel |
| Aly M A | Saad |
| Maha Mohamed | Saber-Ayad |
| Siamak | Sabour |
| Leila | Sabzmakan |
| Basema | Saddik |
| Erfan | Sadeghi |
| Saeid | Sadeghian |
| Amirhossein | Sahebkar |
| Morteza | Saki |
| Saina | Salahi |
| Sarvenaz | Salahi |
| Amir | Salek Farrokhi |
| Marwa Rashad | Salem |
| Hamideh | Salimzadeh |
| Abdallah M | Samy |
| Nizal | Sarrafzadegan |
| Brijesh | Sathian |
| Melika | Shafeghat |
| Syed Mahboob | Shah |
| Jaffer | Shah |
| Ataollah | Shahbandi |
| Fariba | Shahraki-Sanavi |
| Mehran | Shams-Beyranvand |
| Mohd | Shanawaz |
| Kiomars | Sharafi |
| Javad | Sharifi-Rad |
| Jeevan K | Shetty |
| Zahra | Shokri Varniab |
| Seyed Afshin | Shorofi |
| Soraya | Siabani |
| Mohammad Sadegh | Soltani-Zangbar |
| Seidamir Pasha | Tabaeian |
| Seyed-Amir | Tabatabaeizadeh |
| Mohammad | Tabish |
| Majid | Taheri |
| Yasaman | Taheri Abkenar |
| Moslem | Taheri Soodejani |
| Amir | Taherkhani |
| Arash | Tehrani-Banihashemi |
| Mohamad-Hani | Temsah |
| Bereket M | Tigabu |
| Alireza | Vakilian |
| Siavash | Vaziri |
| Bay | Vo |
| Fereshteh | Yazdanpanah |
| Arzu | Yigit |
| Vahit | Yiğit |
| Mazyar | Zahir |
| Burhan Abdullah | Zaman |
| Maryam | Zamanian |
| Moein | Zangiabadian |
| Iman | Zare |
| Zahra | Zareshahrabadi |
| Ali H | Mokdad |
| Mohsen | Naghavi |
| Bagher | Larijani |
| Farshad | Farzadfar† |

* Lead author

† Senior Author
