# Supplementary material for: The burden of metabolic risk factors in North Africa and the Middle East, 1990–2019: findings from the Global Burden of Disease Study
Source: eClinicalMedicine. 2023 Jun 2;60:102022. doi: 10.1016/j.eclinm.2023.102022 (PMC10242634; doi:10.1016/j.eclinm.2023.102022)
Supplement: Caption for Supplementary material [file mmc6.docx]

**Supplementary Table 1.** Detailed citation, location, and time span of input data sources.

**Supplementary Table 2.** Age-standardized death and DALY rates attributable to high-SBP, high-FPG, high-BMI, and high-LDL in 1990, 2010, and 2019 by location.

**Supplementary Table 3.** The percentage of deaths and DALYs of the North Africa and the Middle East region attributable to high-SBP, high-FPG, high-BMI, and high-LDL in 1990 and 2019 by gender and age group.

**Supplementary Table 4.** The percentage of age-standardized deaths and DALYs of the Level 2 causes attributable to high-SBP, high-FPG, high-BMI, and high-LDL in 2019 by location.

**Supplementary Table 5.** Age-standardized SEVs in 1990, 2010, and 2019, and ARC for 1990-2019, 1990-2010, and 2010-2019 periods by location.

**Supplementary Table 6.** Age-standardized DALY rate in 1990, 2010, and 2019, and ARC for 1990-2019, 1990-2010, and 2010-2019 periods by location.

**Supplementary Table 7.** GDR of age-standardized death and DALY rates attributed to high-SBP, high-FPG, high-BMI, and high-LDL in 2019 by location sorted by SDI.
